# Supplementary material for: DNAJC9 integrates heat shock molecular chaperones into the histone chaperone network
Source: Mol Cell. 2021 Jun 17;81(12):2533–2548.e9. doi: 10.1016/j.molcel.2021.03.041 (PMC8221569; doi:10.1016/j.molcel.2021.03.041)
Supplement: Document S1. Figures S1–S6 [file mmc1.pdf]

Molecular Cell, Volume *81*

Supplemental information

DNAJC9 integrates heat shock molecular chaperones into the histone chaperone network

Colin M. Hammond, Hongyu Bao, Ivo A. Hendriks, Massimo Carraro, Alberto García-Nieto, Yanhong Liu, Nazaret Reverón-Gómez, Christos Spanos, Liu Chen, Juri Rappsilber, Michael L. Nielsen, Dinshaw J. Patel, Hongda Huang, and Anja Groth

Figure S1

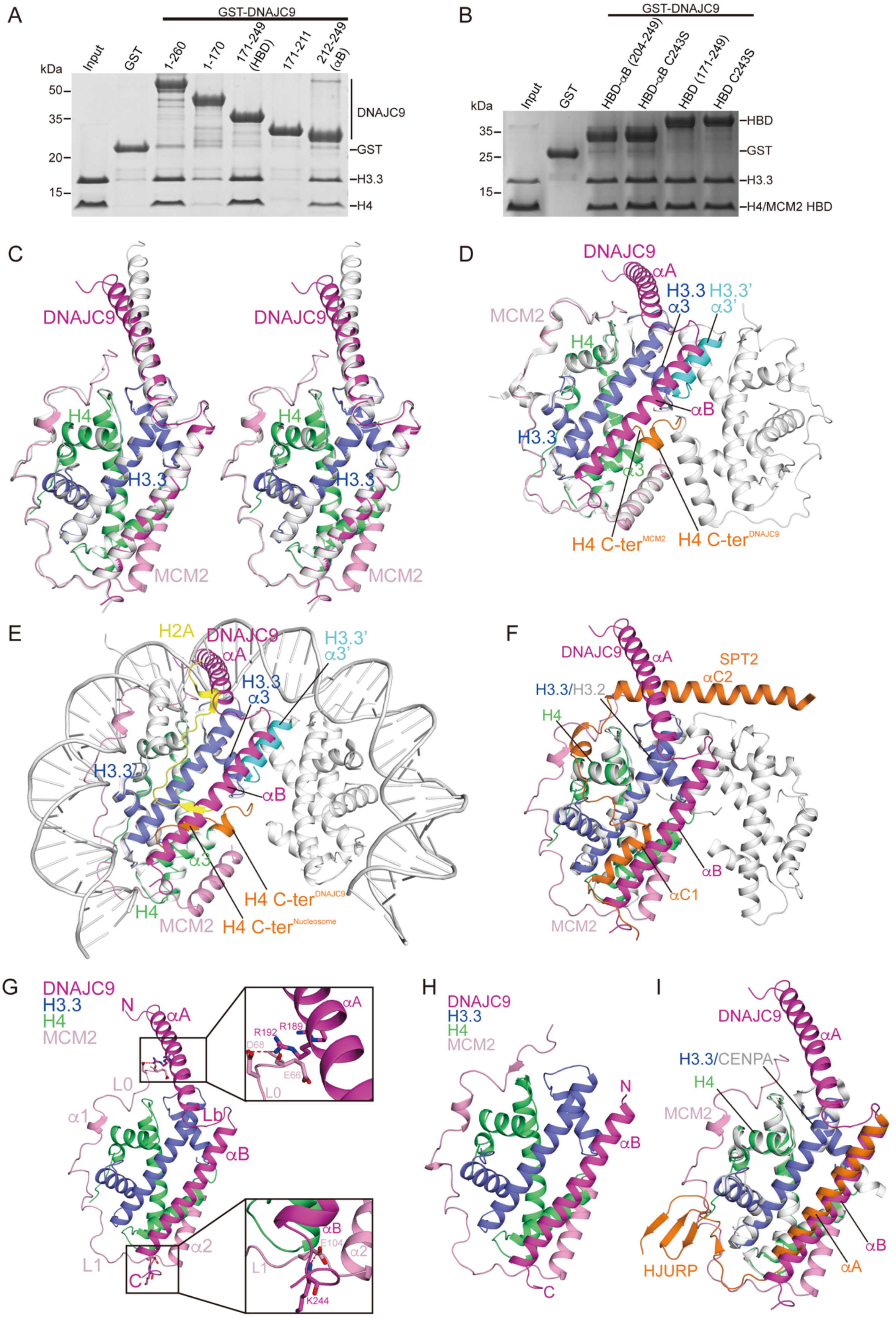

A
GST-DNAJC9
Input
GST
1-260
1-170
171-249 (HBD)
171-211
212-249 (αB)
kDa
50
35
20
15
DNAJC9
GST
H3.3
H4
B
GST-DNAJC9
Input
GST
HBD-αB (204-249)
HBD-αB C243S
HBD (171-249)
HBD C243S
kDa
35
25
15
HBD
GST
H3.3
H4/MCM2 HBD
C
DNAJC9
H4
H3.3
MCM2
DNAJC9
H4
H3.3
MCM2
D
DNAJC9
αA
MCM2
H3.3
α3
H3.3'
α3'
H4
αB
H3.3
α3
H4 C-ter MCM2
H4 C-ter DNAJC9
E
H2A
DNAJC9
αA
H3.3'
α3'
H3.3
α3
H3.3
αB
α3
H4
MCM2
H4 C-ter DNAJC9
H4 C-ter Nucleosome
F
DNAJC9
αA
SPT2
αC2
H3.3/H3.2
H4
αB
αC1
MCM2
G
DNAJC9
H3.3
H4
MCM2
N
αA
R192
R189
D68
E66
L0
L0
α1
Lb
αB
α2
L1
C
αB
E104
α2
L1
K244
H
DNAJC9
H3.3
H4
MCM2
N
αB
C
I
DNAJC9
αA
H3.3/CENPA
H4
MCM2
αB
HJURP
αA

Figure S1. Biochemical and Structural Characterisation of the DNAJC9 HBD – Related to Figure 2 and Table 1

(A) Truncation mapping of DNAJC9 histone binding domain (HBD) using GST-pulldown. The pulldowns were between GST-DNAJC9 fragments and histone H3.3–H4, similar to Figure 2B but without the MCM2 HBD.

(B) Pulldowns showing that C243S mutation on DNAJC9 HBD or αB did not influence histone binding.

(C) Stereo view showing structural comparison of the two copies of the DNAJC9 HBD–H3.3–H4–MCM2 HBD quaternary complexes in the crystallographic asymmetric unit. One complex is color-coded DNAJC9 HBD (magenta), H3.3 (blue), H4 (green) and MCM2 HBD (pink), while the other is coloured silver. The color-coded complex is the form presented in Figure 2C. The two complex structures superimposed well with a small root-mean-square deviation (rmsd) of 0.47 Å.

(D) Superimposition of the DNAJC9 HBD–H3.3–H4–MCM2 HBD complex (colored in C) with the MCM2 HBD–H3.3–H4 complex (silver; PDB 5BNV (Huang et al., 2015)). The αB helix of DNAJC9 HBD sterically occludes the H3.3' α3' helix (cyan) to block H3.3–H4 tetramerisation seen in the MCM2 HBD–H3.3–H4 complex. The H4 C-terminus ('C-ter'; orange) adopts a helical conformation upon DNAJC9 HBD binding, while it is partially disordered in the MCM2 HBD–H3.3–H4 complex.

(**E**) Superimposition of the DNAJC9 HBD–H3.3–H4–MCM2 HBD complex (colored in C) with the H3.3–H4 tetramer from a partial representation of the nucleosome (silver; PDB 3AV2 (Tachiwana et al., 2011)). The αB helix of DNAJC9 HBD sterically occludes the H3.3' α3' helix (cyan) to block H3–H4 tetramerisation observed in the nucleosome. The H4 C-terminus ('C-ter'; orange) adopts a helical conformation upon DNAJC9 HBD binding, while it forms a β-strand with the H2A docking domain (yellow) in the nucleosome.

(**F**) Superimposition of the DNAJC9 HBD–H3.3–H4–MCM2 HBD complex (colored in C) and the SPT2–H3.2–H4 complex (H3.2–H4 tetramer, silver; SPT2, orange; PDB 5BS7 (Chen et al., 2015)), showing that observed binding modes of DNAJC9 HBD and SPT2 HBD with histone H3–H4 are mutually incompatible.

(G) DNAJC9 HBD contacts MCM2 HBD in two points with interacting residues highlighted (see magnified inserts; colored in C).

(H) Overall structure of the DNAJC9 αB–H3.3–H4–MCM2 HBD quaternary complex in cartoon representation (DNAJC9 αB, magenta; H3.3, blue; H4, green; MCM2 HBD, pink). This structure is almost identical to its counterpart containing the DNAJC9 HBD (rmsd 0.38 Å, Figure 2C). Data collection and refinement statistics presented in Table 1.

(I) Superimposition of the DNAJC9 HBD–H3.3–H4–MCM2 HBD complex (colored in C) and the HJURP–CENPA–H4 complex (CENPA–H4 dimer, silver; HJURP, orange; PDB 3R45 (Hu et al., 2011)), revealing that the αB helix of DNAJC9 HBD has a similar structural configuration to the αA helix of HJURP.

Figure S2

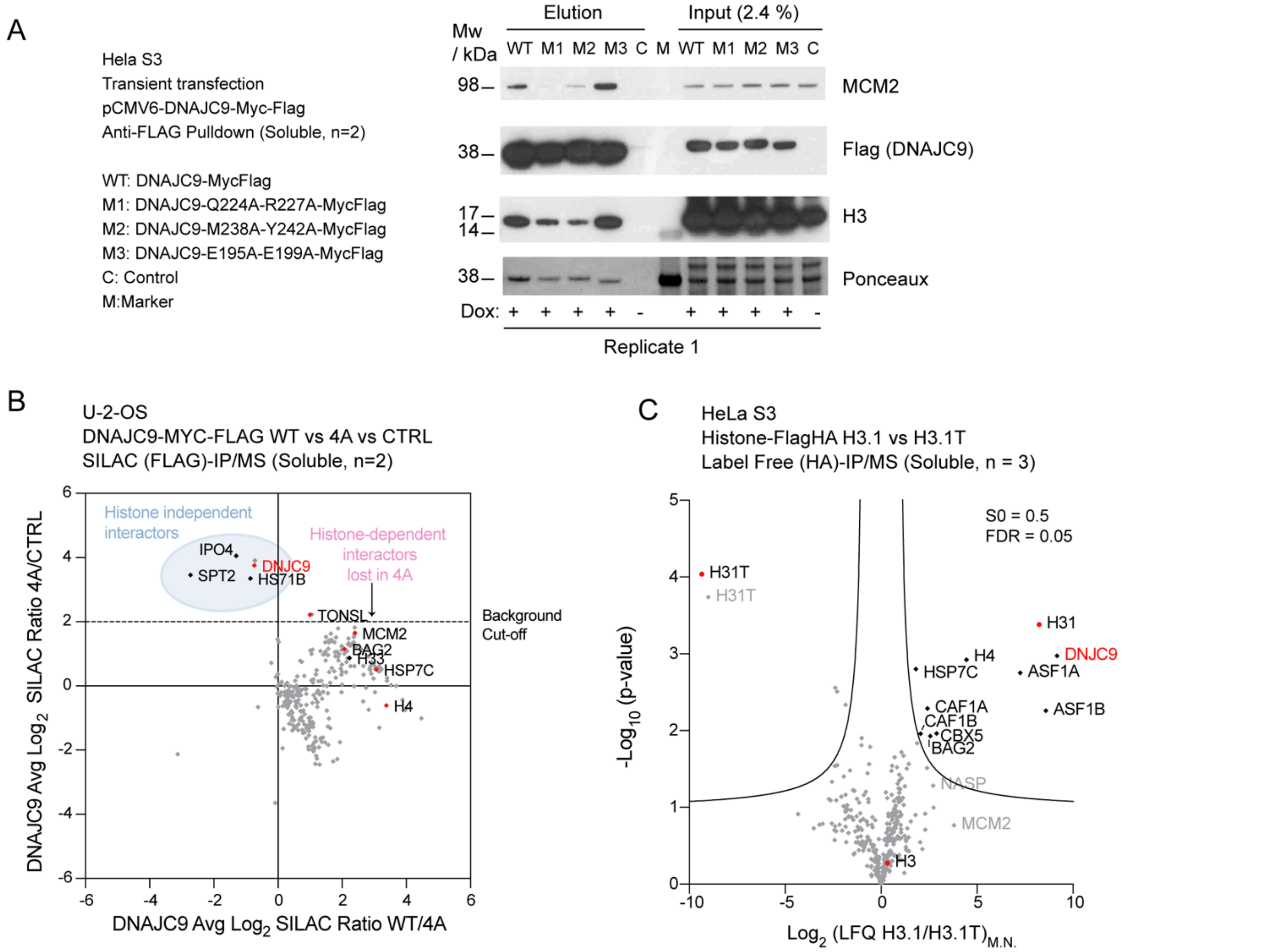

Figure S2. Analysis of DNAJC9 Histone Binding Mutants and H3.1T Interactome - Related to Figure 4

(A) Pull-downs from soluble extracts of DNAJC9 WT and mutant proteins expressed by transient transfection and analysed by Western blotting (representative of n=2 biological replicates).

(B) DNAJC9 WT, 4A mutant and control purifications subjected to triple SILAC based mass spectrometry analysis. Data from the same experiment is shown with WT/C on the x-axis in Figure 4A. This plot highlights a histone-independent interaction between DNAJC9 and SPT2. Ratios averaged from n=2 biological replicates.

(C) Analysis of the H3.1T interactome. Histone purifications from soluble extracts subjected to label-free mass spectrometry analysis (n=3 biological replicates, s0 = 0.5, FDR = 0.05). Volcano plots represent differences in median normalised LFQ intensities ($LFQ_{M.N.}$) with missing values imputed for factors observed 3 times in either condition. Diamonds, protein level LFQ quantification; Red dots, median ratios and p values from peptide level LFQ intensities for peptides specific to either H3.1 (H31) or H3.1T (H31T), or shared H3 peptides (H3).

(B and C) Proteins referred to by human UniProt protein identification code. See also Table S1.

Figure S3

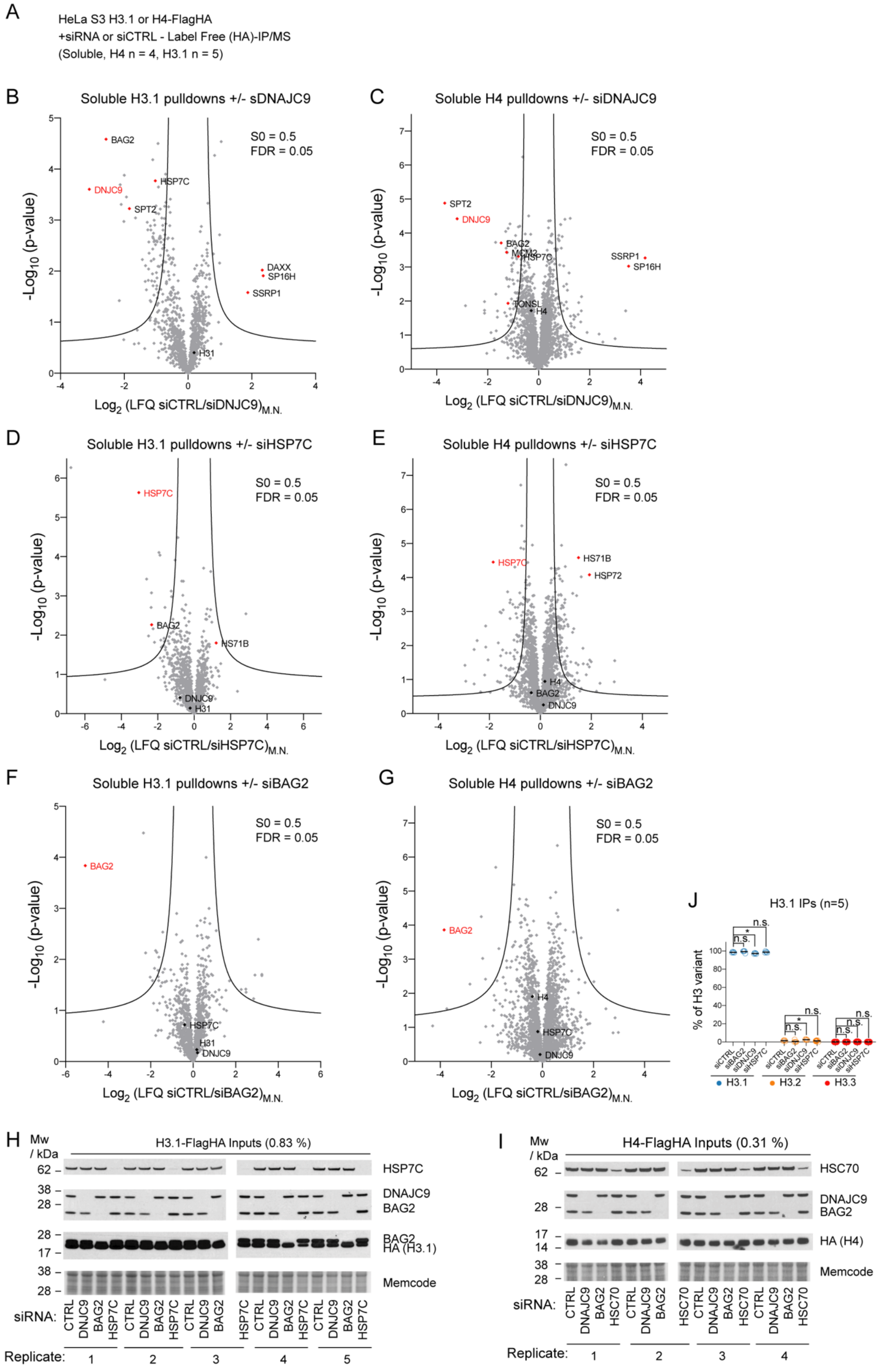
A
HeLa S3 H3.1 or H4-FlagHA
+siRNA or siCTRL - Label Free (HA)-IP/MS
(Soluble, H4 n = 4, H3.1 n = 5)
B
Soluble H3.1 pulldowns +/- sDNAJC9
S0 = 0.5
FDR = 0.05
BAG2
DNJC9
HSP7C
SPT2
DAXX
SP16H
SSRP1
H31
-Log10 (p-value)
Log2 (LFQ siCTRL/siDNJC9)M.N.
C
Soluble H4 pulldowns +/- siDNAJC9
SPT2
DNJC9
BAG2
MCM2
HSP7C
SSRP1
SP16H
TONSL
H4
Log2 (LFQ siCTRL/siDNJC9)M.N.
D
Soluble H3.1 pulldowns +/- siHSP7C
HSP7C
BAG2
HS71B
DNJC9
H31
Log2 (LFQ siCTRL/siHSP7C)M.N.
E
Soluble H4 pulldowns +/- siHSP7C
HSP7C
HS71B
HSP72
H4
BAG2
DNJC9
Log2 (LFQ siCTRL/siHSP7C)M.N.
F
Soluble H3.1 pulldowns +/- siBAG2
BAG2
HSP7C
H31
DNJC9
Log2 (LFQ siCTRL/siBAG2)M.N.
G
Soluble H4 pulldowns +/- siBAG2
BAG2
H4
HSP7C
DNJC9
Log2 (LFQ siCTRL/siBAG2)M.N.
J
H3.1 IPs (n=5)
% of H3 variant
n.s.
*
siCTRL
siBAG2
siDNJC9
siHSP7C
H3.1
H3.2
H3.3
H
Mw / kDa
H3.1-FlagHA Inputs (0.83 %)
HSP7C
DNAJC9
BAG2
HA (H3.1)
Memcode
siRNA:
Replicate:
I
H4-FlagHA Inputs (0.31 %)
HSC70
DNAJC9
BAG2
HA (H4)
Memcode

Figure S3. Functional Proteomic Analysis of Histone H3.1 and H4 Complexes Using siRNA Depletion of DNAJC9 and the Heat Shock Machinery – Related to Figure 4

(A-G) (A) Histone H3.1 and H4 purifications from soluble extracts siRNA depleted of DNAJC9 (**B-C**), BAG2 (**D-E**) or HSC7C (**F**-**G)** compared to control siRNA depletions and subjected to label-free mass spectrometry analysis (H3.1 n=5 and H4 n=4 biological replicates, s0 = 0.5, FDR = 0.05). Volcano plots represent differences in median-normalised LFQ intensities ($LFQ_{M.N.}$) with missing values imputed for factors observed in all replicates of either condition for each volcano plot comparison.

(**H and I**)**,** Western blot controls for siRNA depletion in extracts used for (**H**) H3.1 and (**I)** H4 pulldowns.

(**J**) Quantitation of the variant specific peptides showing a small but significant increase in H3.2 (P = 0.039) but not H3.3 peptides (P = 0.969) in H3.1 pulldowns depleted for DNAJC9 with no significant variant peptide gains in H3.1 pulldowns depleted for HSP7C (H3.2 P = 0.969, H3.3 P >0.999) and BAG2 (H3.2 P = 0.215, H3.3 P >0.999). P values represent unpaired t-tests Holm-Šídák corrected, alpha = 0.05, assuming sampling from populations with constant standard deviation. See also Table S2.

(**B-G**) Proteins referred to by human UniProt protein identification code. See also Table S1.

Figure S4

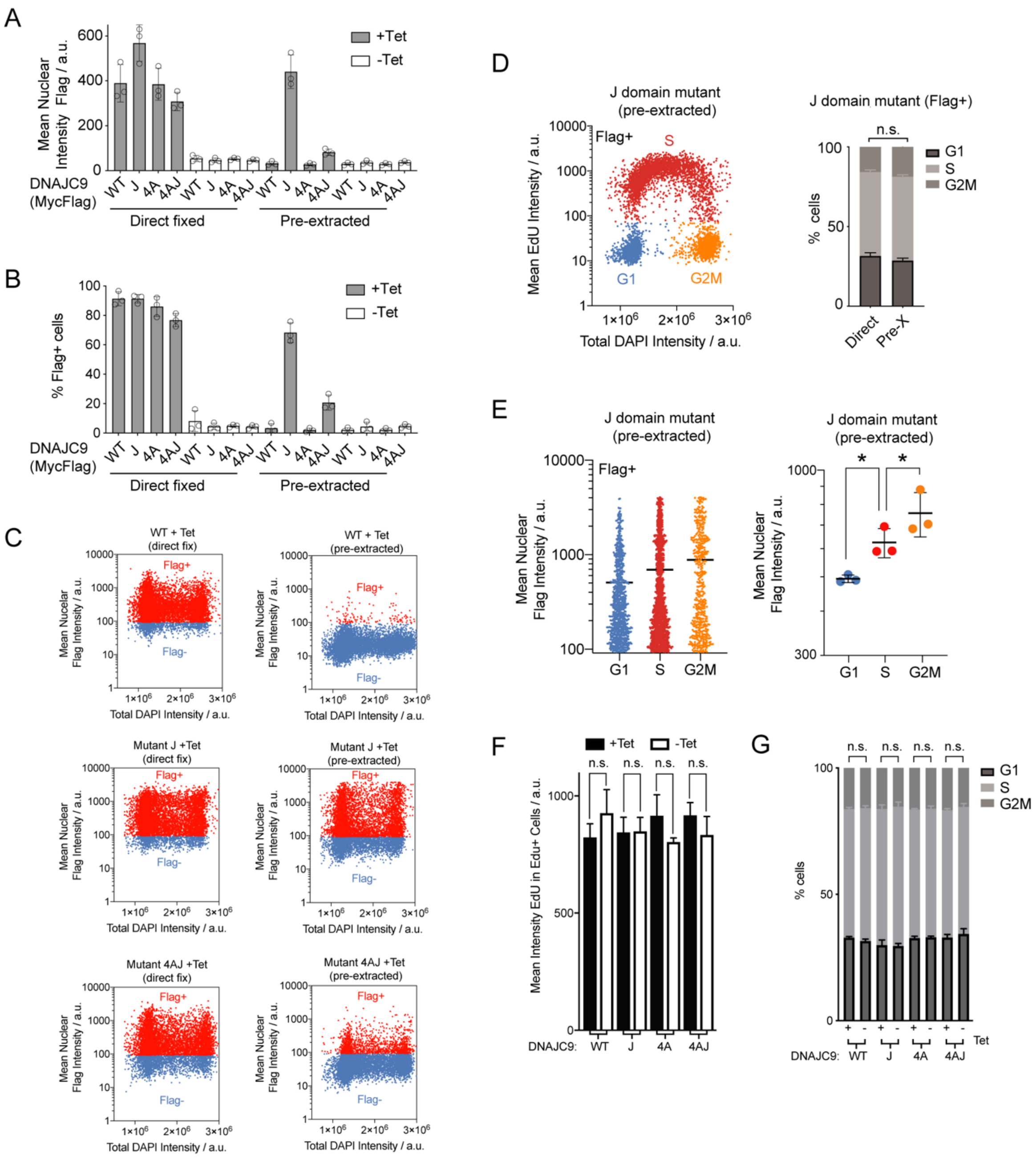
A
Mean Nuclear Intensity Flag / a.u.
+Tet
-Tet
DNAJC9 (MycFlag)
WT J 4A 4AJ WT J 4A 4AJ WT J 4A 4AJ WT J 4A 4AJ
Direct fixed
Pre-extracted
B
% Flag+ cells
C
WT + Tet (direct fix)
WT + Tet (pre-extracted)
Mutant J +Tet (direct fix)
Mutant J +Tet (pre-extracted)
Mutant 4AJ +Tet (direct fix)
Mutant 4AJ +Tet (pre-extracted)
Flag+
Flag-
Mean Nucelar Flag Intensity / a.u.
Total DAPI Intensity / a.u.
D
J domain mutant (pre-extracted)
Mean EdU Intensity / a.u.
Flag+
G1
S
G2M
J domain mutant (Flag+)
n.s.
% cells
Direct
Pre-X
E
J domain mutant (pre-extracted)
Mean Nuclear Flag Intensity / a.u.
F
+Tet
-Tet
n.s.
Mean Intensity EdU in Edu+ Cells / a.u.
DNAJC9: WT J 4A 4AJ
G
% cells
Tet
DNAJC9: WT J 4A 4AJ

Figure S4. The DNAJC9 J Mutant is Trapped on Chromatin in a Histone Dependent Manner Throughout the Cell Cycle – Related to Figure 5

(A-G) High-content microscopy of DNAJC9-MYC-Flag WT and mutants induced by tetracycline (+Tet) in direct fixed (total) or pre-extracted (chromatin-bound) U-2-OS cells pulsed with EdU (n=3 biological replicates). See also Table S2.

(A) Mean flag intensity in nuclei defined by DAPI (error bars represent mean ± s.d).

(B) Percentage of Flag positive cells (error bars represent mean ± s.d).

(C) Gating strategy for identification of Flag positive cells in **(B)**

(D) Left, Representative cell cycle gating showing G1, S and G2/M populations of pre-extracted Flag positive DNAJC9 J mutant cells. Right, Mean cell cycle populations ± s.d. of Flag positive DNAJC9 J mutant cells comparing direct or pre-extracted cells (for G1, S and G2M comparisons, adjusted P = 0.092, >0.999, 0.092, unpaired t-tests Holm-Šídák corrected, alpha = 0.05, assuming sampling from populations with constant standard deviation).

(E) Mean nuclear Flag intensities in cell cycle gated populations for pre-extracted DNAJC9 J mutant cells (Flag positive) (Left; representative example, means indicated) and experimental means shown (Right; from left, P = 0.0404 and 0.0456, paired two-sided t-tests, error bars represent mean ± s.d.).

(F) Mean EdU intensities ± s.d. in EdU positive cells comparing intensities with and without the expression (+/- Tet) of DNAJC9 WT and mutants (from left, adjusted P = 0.7127, 0.9872, 0.6763, 0.7736, unpaired t-tests Holm-Šídák corrected, alpha = 0.05, assuming sampling from populations with constant standard deviation).

(G) Mean cell cycle populations ± s.d. in direct fixed cells, comparing population percentages with and without expression (+/- Tet) of DNAJC9 WT and mutants (from left for G1, adjusted P = 0.9470, 0.9998, 0.9998 and 0.9439; from left for S, adjusted P = 0.7540, 0.9608, >0.9999 and 0.9998; from left for G2M, adjusted P = 0.9998, 0.9962, 0.9998 and 0.9594, unpaired t-tests Holm-Šídák corrected, alpha = 0.05, assuming sampling from populations with constant standard deviation)

Figure S5

A

MS Analysis Workflow

(1) Raw data (3224 proteins)

(2) Filtering reverse hits, only identified by site and contaminants (3076 proteins)

(3) Filtering for 6/6 observations in WT or J or 4A (1654 proteins)

(4) Filtering for enrichment over beads in any condition (464 proteins combined) - panel B

(5) Bait normalisation - panel C

(6) WT vs 4A and WT vs J volcano plots - no imputed values - panels D & E

(7) For factors not identified in 6/6 control samples WT/J and WT/4A ratios were imputed, filtered and normalised similarly (177 proteins)

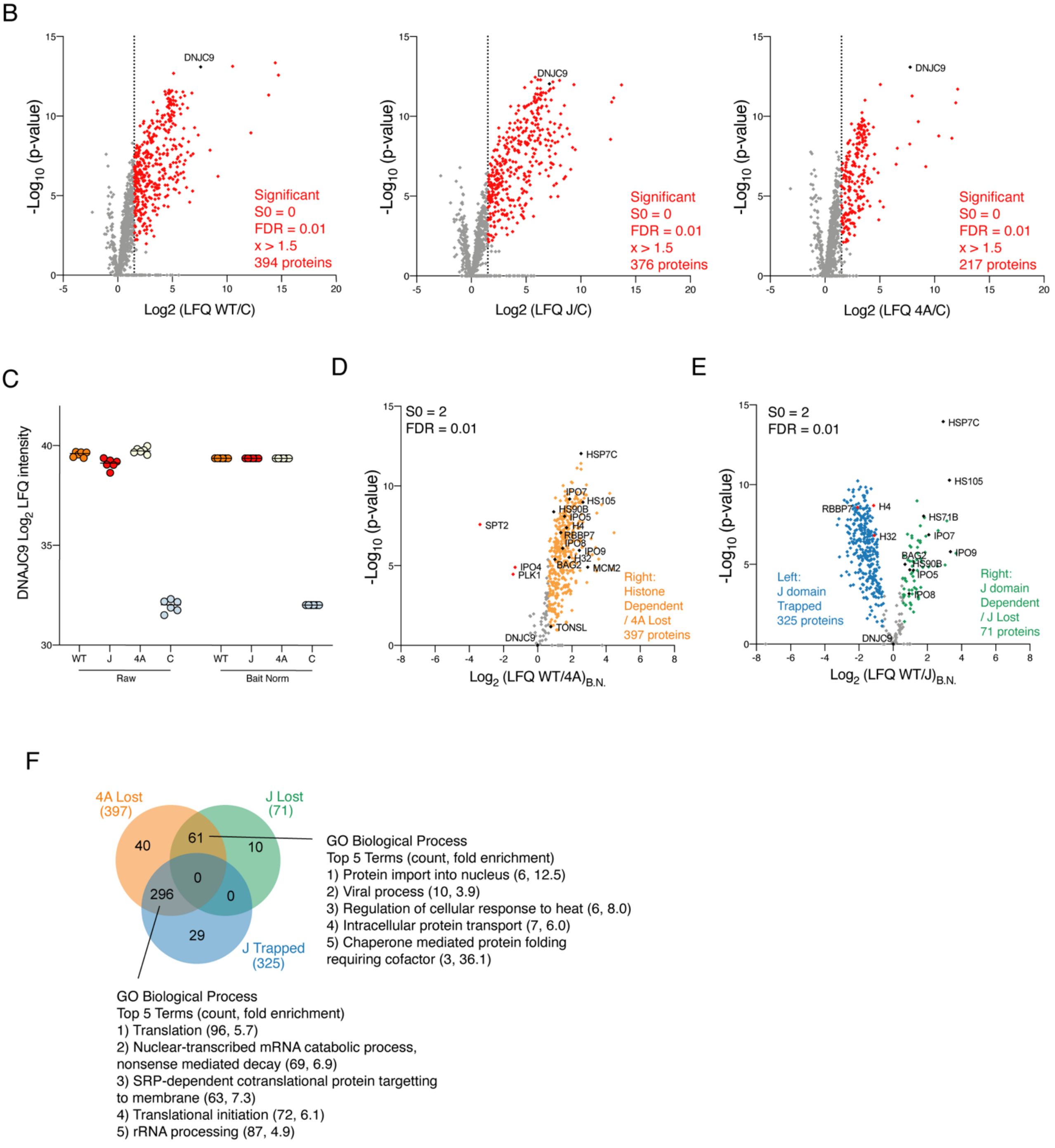

Figure S5. Mass Spectrometry Analysis of DNAJC9 WT, J and 4A vs Control Purifications from Soluble Extracts – Related to Figure 6

(A) Overview of data analysis workflow. Data from n=6 biological replicates.

(B) Volcano plots of showing enrichment of factors associated with soluble DNAJC9 WT and mutants over beads. Factors highlighted in red are significant (s0 = 0, FDR = 0.01) and have a Log2 fold change of >1.5 in their ratio of LFQ intensity (DNAJC9/control). LFQ intensities of factors that met these filtering criteria for WT, J or 4A purifications were subsequently bait normalised.

(C) DNAJC9 LFQ intensity in purifications before and after bait normalization. See also Table S2.

(D and E) Volcano plots of showing enrichment of factors with DNAJC9 WT compared to mutants 4A **(D)** and J **(E)**, ratios represent bait normalized LFQ intensities. Colours were used to highlight factors that show statistical differences in DNAJC9 WT and mutant comparisons (s0 = 2, FDR = 0.01), with the exception of DNAJC9 proteins labelled show significant differences.

(F) Venn diagrams (InteractiVenn (Heberle et al., 2015)) colour coded as in **(D-E)**, showing the overlap of factors classified as statistically changing between WT vs mutant. The top 5 GO biological process direct terms from indicated regions of Venn diagram from DAVID (Huang da et al., 2009a, b) version 6.8 analysis with the number of GO term associated proteins and fold change indicated compared to all factors identified (after filtering reverse hits, only identified by site and contaminants - step 2 in **(A)**); EASE = 0.01. See also Table S2.

(B, D and E) Proteins referred to by human UniProt protein identification code. See also Table S1.

Figure S6

A

MS Analysis Workflow

(1) Raw data (2693 proteins)

(2) Filtering reverse hits, only identified by site and contaminants (2562 proteins)

(3) Filtering for 6/6 observations in WT or J or 4A (1613 proteins)

(4) Filtering for enrichment over beads in any condition - panel B (422 proteins combined)

(5) Bait normalisation - panel C

(6) WT vs 4A and WT vs J volcano plots - no imputed values - panels D & E

(7) For factors not identified in 6/6 control samples WT/J and WT/4A ratios were imputed, filtered and normalised similarly (26 extra proteins) - panel G

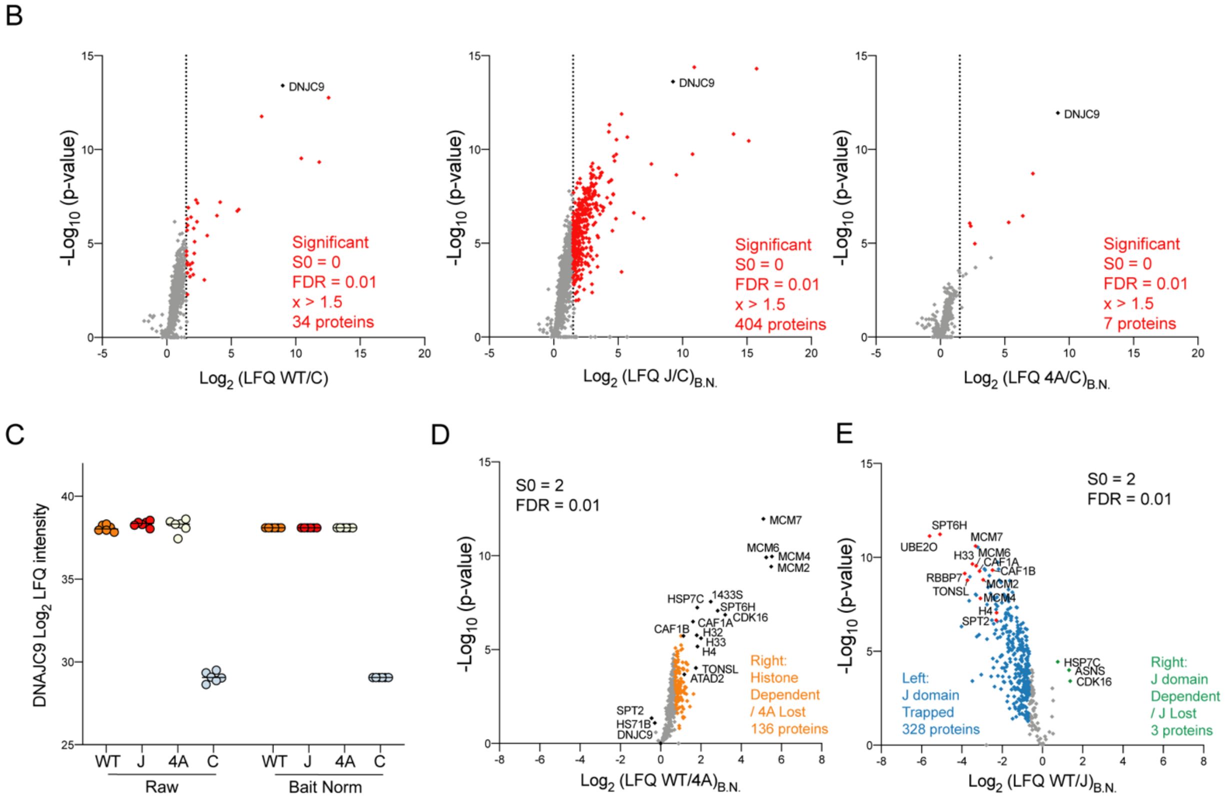

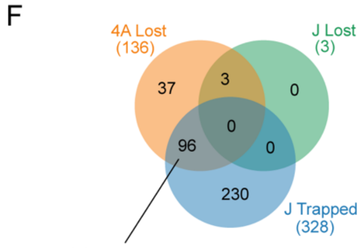

GO Biological Process
Top 10 Terms (count, fold enrichment)
1) rRNA processing (28, 4.3)
2) Maturation of SSU-rRNA from tricistronic rRNA transcript (11, 9.6)
3) Gene silencing by RNA (12, 6.1)
4) DNA replication dependent nucleosome assembly (6, 19.1)
5) DNA replication independent nucleosome assembly (5, 21.2)
6) DNA Replication (11, 5)
7) Negative regulation of gene expression, epigenetic (6, 11.7)
8) Chromatin assembly (4, 20.3)
9) Nucleosome assembly (7, 5.7)
10) Maturation of LSU-rRNA from tricistronic rRNA transcript (4, 11.3)

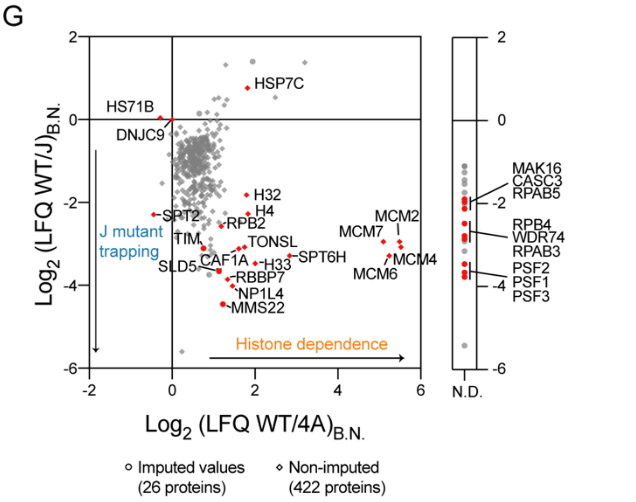

Figure S6. Mass Spectrometry Analysis of DNAJC9 WT, J and 4A vs Control Purifications from Chromatin Extracts – Related to Figure 6

(A) Overview of data analysis workflow. Data from n=6 biological replicates.

(B) Volcano plots of showing enrichment of factors associated with chromatin bound DNAJC9 WT and mutants over beads. Factors highlighted in red are significant (s0 = 0, FDR = 0.01) and have a Log2 fold change of >1.5 in their ratio of LFQ intensity (DNAJC9/control). LFQ intensities of factors that met these filtering criteria for WT, J or 4A purifications were subsequently bait normalised.

(C) DNAJC9 LFQ intensity in purifications before and after bait normalization. See also Table S2.

(D and E) Volcano plots of showing enrichment of factors with DNAJC9 WT compared to mutants 4A **(D)** and J **(E)**, ratios represent bait normalized LFQ intensities. Colours were used to highlight factors that show statistical differences in DNAJC9 WT and mutant comparisons (s0 = 2, FDR = 0.01), with the exception of DNAJC9 proteins labelled show significant differences.

(F) Venn diagrams (InteractiVenn (Heberle et al., 2015)) colour coded as in **(D-E)**, showing the overlap of factors classified as statistically changing between WT vs mutant. The top 5 GO biological process direct terms from indicated regions of Venn diagram from DAVID (Huang da et al., 2009a, b) version 6.8 analysis with the number of GO term associated proteins and fold change indicated compared to all factors identified (after filtering reverse hits, only identified by site and contaminants - step 2 in **(A)**); EASE = 0.01. See also Table S2.

(G) Scatter plot of DNAJC9 WT vs 4A and WT vs J ratios; all factors that met the statistical filtering without imputation (diamonds) and additionally factors imputed (circles) that were not identified in 6/6 control samples but otherwise met filtering requirements set out in (**A**).

(B, D, E and G) Proteins referred to by human UniProt protein identification code. See also Table S1.
