# Supplementary material for: DNAJC9 integrates heat shock molecular chaperones into the histone chaperone network
Source: Mol Cell. 2021 Jun 17;81(12):2533–2548.e9. doi: 10.1016/j.molcel.2021.03.041 (PMC8221569; doi:10.1016/j.molcel.2021.03.041)
Supplement: Document S2. Article plus supplemental information [file mmc4.pdf]

Molecular Cell

Article

DNAJC9 integrates heat shock molecular chaperones into the histone chaperone network

Graphical abstract

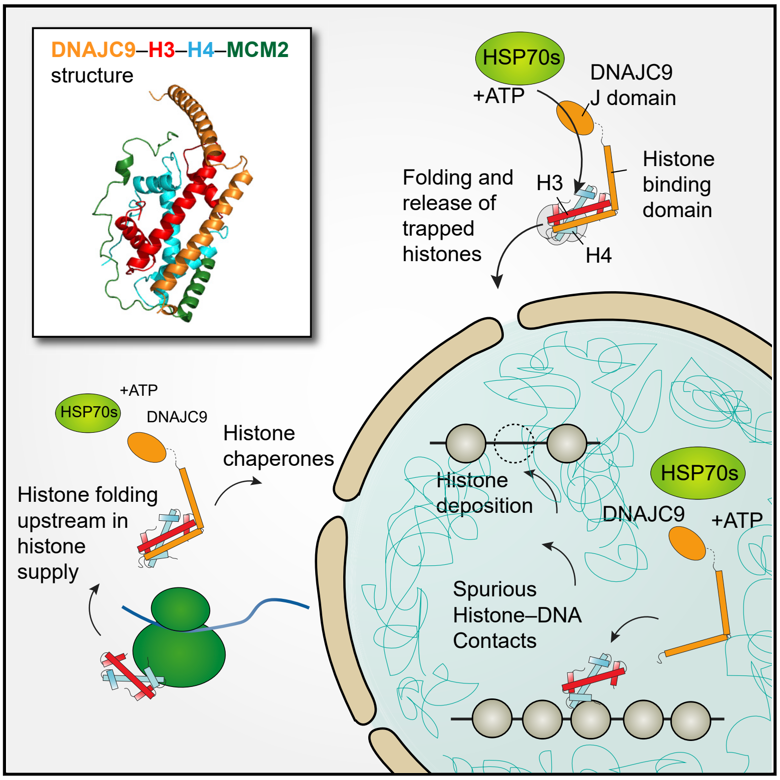

Authors

Colin M. Hammond, Hongyu Bao, Ivo A. Hendriks, ..., Dinshaw J. Patel, Hongda Huang, Anja Groth

Correspondence

pateld@mskcc.org (D.J.P.),
huanghd@sustech.edu.cn (H.H.),
anja.groth@cpr.ku.dk (A.G.)

In brief

Hammond et al. identified and characterized a histone chaperone functionality in the heat shock co-chaperone DNAJC9. DNAJC9 directs molecular chaperones to histone substrates, facilitating their supply to and transactions within chromatin. This study highlights that histone chaperones and molecular chaperones combine to fully protect histone proteins on route to chromatin.

Highlights

- DNAJC9 is a dual histone chaperone and heat shock co-chaperone
- Crystal structure reveals the binding mode of DNAJC9 with H3-H4 dimers
- DNAJC9 recruits HSP70 enzymes to guard histone structural integrity
- ATP-dependent protein folding is integral to histone chaperone biology

Hammond et al., 2021, Molecular Cell *81*, 2533–2548
June 17, 2021 © 2021 The Authors. Published by Elsevier Inc.
https://doi.org/10.1016/j.molcel.2021.03.041

CellPress

Molecular Cell

CellPress
OPEN ACCESS

Article

DNAJC9 integrates heat shock molecular chaperones into the histone chaperone network

Colin M. Hammond,[1,2,8] Hongyu Bao,[3,8] Ivo A. Hendriks,[1] Massimo Carraro,[1,2] Alberto García-Nieto,[2,7] Yanhong Liu,[3] Nazaret Reverón-Gómez,[1,2] Christos Spanos,[4] Liu Chen,[3] Juri Rappsilber,[4,5] Michael L. Nielsen,[1] Dinshaw J. Patel,[6,*] Hongda Huang,[3,*] and Anja Groth[1,2,9,*]

[1]Novo Nordisk Foundation Center for Protein Research (CPR), University of Copenhagen, Copenhagen, Denmark
[2]Biotech Research and Innovation Centre (BRIC), University of Copenhagen, Copenhagen, Denmark
[3]Key Laboratory of Molecular Design for Plant Cell Factory of Guangdong Higher Education Institutes, Department of Biology, School of Life Sciences, Southern University of Science and Technology, Shenzhen 518055, China
[4]Wellcome Centre for Cell Biology, University of Edinburgh, Edinburgh, UK
[5]Bioanalytics, Institute of Biotechnology, Technische Universität Berlin, Berlin, Germany
[6]Structural Biology Program, Memorial Sloan Kettering Cancer Center, New York, NY 10065, USA
[7]Present address: Division of Cell Biology, Netherlands Cancer Institute, Plesmanlaan 121, 1066 CX Amsterdam, the Netherlands
[8]These authors contributed equally
[9]Lead contact
*Correspondence: pateld@mskcc.org (D.J.P.), huanghd@sustech.edu.cn (H.H.), anja.groth@cpr.ku.dk (A.G.)
https://doi.org/10.1016/j.molcel.2021.03.041

SUMMARY

From biosynthesis to assembly into nucleosomes, histones are handed through a cascade of histone chaperones, which shield histones from non-specific interactions. Whether mechanisms exist to safeguard the histone fold during histone chaperone handover events or to release trapped intermediates is unclear. Using structure-guided and functional proteomics, we identify and characterize a histone chaperone function of DNAJC9, a heat shock co-chaperone that promotes HSP70-mediated catalysis. We elucidate the structure of DNAJC9, in a histone H3-H4 co-chaperone complex with MCM2, revealing how this dual histone and heat shock co-chaperone binds histone substrates. We show that DNAJC9 recruits HSP70-type enzymes via its J domain to fold histone H3-H4 substrates: upstream in the histone supply chain, during replication- and transcription-coupled nucleosome assembly, and to clean up spurious interactions. With its dual functionality, DNAJC9 integrates ATP-resourced protein folding into the histone supply pathway to resolve aberrant intermediates throughout the dynamic lives of histones.

INTRODUCTION

In its simplest form, chromatin consists of an array of repeating subunits called nucleosomes, each of which contains a complex of eight interwoven histone proteins that wrap 146 bp of DNA in a left-handed superhelix (Luger et al., 1997). The positioning and composition of nucleosomes governs access to DNA, thereby affecting all aspects of genome function, including cellular gene expression programs, silencing of repetitive elements, and DNA repair (Allis and Jenuwein, 2016; Lai and Pugh, 2017; Nicetto and Zaret, 2019; Yadav et al., 2018). Given the high affinity of histones for both DNA and RNA, navigating the cellular landscape from synthesis on the ribosome to a specific site in the genome is a major challenge in histone supply. Throughout this process, histone chaperones shield histones from spurious interactions by binding to the exact same surfaces of histones required for nucleosome assembly (Hammond et al., 2017; Mattiroli et al., 2015; Pardal et al., 2019). In this manner, histone chaperones buffer the interactions of histones with DNA, ensuring the formation of proper nucleosomal contacts during the assembly process (Andrews et al., 2010). Histone chaperone functionality is also physically integrated within and tethered to ATP-driven enzymes, including DNA and RNA polymerases, helicases, and remodeling enzymes (Hammond et al., 2017). For example, histone chaperone activity is provided by SPT2, SPT6, HIRA, and FACT during transcription and CAF-1, MCM2, and FACT during DNA replication, while DAXX integrates with the nucleosome remodeling enzyme ATRX (Hammond et al., 2017). Histone chaperones thereby provide pathways for histone recycling, *de novo* deposition, and exchange, which are central to the maintenance and plasticity of chromatin (Gurard-Levin et al., 2014; Hammond et al., 2017).

An emerging theme in histone chaperone biology is that histone chaperone capabilities are often distributed across several proteins so that no one protein forms a complete shield around their histone cargo. Rather histone chaperones tend to collaborate in histone co-chaperone complexes in which two or more histone chaperones associate in an interaction bridged by a single histone fold-dimer (Hammond et al., 2017). This modular framework provides opportunities to fine-tune the function of each histone chaperone complex, through the removal or replacement of histone co-chaperone partners. This flexibility

© 2021 The Authors. Published by Elsevier Inc.
This is an open access article under the CC BY license (http://creativecommons.org/licenses/by/4.0/).

allows the histone chaperone network to efficiently integrate histone supply with a large range of chromatin processes. Another defining feature of histone chaperone activity is the ATP-independent mode of action (Elsässer and D'Arcy, 2013), which contrasts to the heat shock molecular chaperones that use ATP to remodel proteins and protein complexes more generally (Clerico et al., 2019; Genest et al., 2019; Rosenzweig et al., 2019). Heat shock chaperones have been implicated in the initial folding of histones (Alvarez et al., 2011; Campos et al., 2010) and in their degradation (Cook et al., 2011). Beyond these processes, histone supply is otherwise understood to be independent of heat shock-mediated protein folding and orchestrated through "histone handover" events between histone chaperones (Hammond et al., 2017; Pardal et al., 2019).

Histones H3 and H4 are thought to engage the histone chaperone supply network shortly after biosynthesis by first engaging the histone chaperone NASP (Campos et al., 2010). NASP stimulates the ATPase activity of HSP90 (Alekseev et al., 2005) and can bind H3 monomers and H3-H4 dimers (Bowman et al., 2017). These attributes could allow NASP to play an active role in the co-folding of H3-H4 *in vivo*; alternatively, they could reflect a HSP90-dependent holdase function (Genest et al., 2019), allowing NASP to protect newly synthesized H3 from degradation (Cook et al., 2011). Once folded, histone H3-H4 dimers associate with ASF1, a central node in the histone chaperone supply network (Hammond et al., 2017). ASF1 has two isoforms, ASF1A and ASF1B, that share conserved histone binding modes (English et al., 2006; Huang et al., 2015; Natsume et al., 2007). The isoforms are differentially regulated (Corpet et al., 2011) but function in a partly redundant manner (Groth et al., 2005, 2007). ASF1 forms histone-dependent interactions with several histone chaperones during supply, including MCM2, TONSL, NASP, Importin-4 (IPO4), RbAp46/48 (RBBP7/RBBP4), and Vps75 in yeast (Campos et al., 2010, 2015; Groth et al., 2007; Hammond et al., 2016; Jasencakova et al., 2010; Saredi et al., 2016). ASF1 also directly associates with the deposition chaperones CAF-1 and HIRA (Daganzo et al., 2003; Mello et al., 2002; Tyler et al., 2001) through a similar interaction motif (Malay et al., 2008; Tang et al., 2006). Additional binding constraints allow ASF1A and ASF1B to specify a preference for HIRA and CAF-1, respectively (Tagami et al., 2004; Tang et al., 2006; Zhang et al., 2005). This culminates in the deposition of H3.1/2-H4 by CAF-1 and H3.3-H4 by HIRA at sites of DNA replication and transcription, respectively (Tagami et al., 2004).

MCM2 is an integral part of the CMG helicase (CDC4, MCM2–7, and GINS) that unwinds DNA prior to its replication, and contains a histone H3-H4 binding domain (Foltman et al., 2013; Groth et al., 2007; Huang et al., 2015; Richet et al., 2015; Wang et al., 2015) that promotes the balanced inheritance of parental histones to both nascent DNA strands (Gan et al., 2018; Petryk et al., 2018). Intriguingly, MCM2 also acts as a chaperone for newly synthesized histones prior to their deposition onto DNA. In this capacity, MCM2 forms co-chaperone partnerships with ASF1 and TONSL (Groth et al., 2007; Huang et al., 2015; Jasencakova et al., 2010; Saredi et al., 2016). ASF1 binds histone H3-H4 dimers by blocking their tetramerization interface (English et al., 2006; Natsume et al., 2007), and TONSL binds the H4 tail unmethylated at lysine 20 (Saredi et al., 2016), a mark of newly synthesized histone H4 (Alabert et al., 2015; Saredi et al., 2016). TONSL possesses both histone chaperone and histone reader activities (Campos et al., 2015; Saredi et al., 2016). Meanwhile, MCM2 chaperones the DNA and H2A-H2B binding surfaces of histone H3-H4 (Huang et al., 2015; Richet et al., 2015; Wang et al., 2015) and when combined with co-chaperones ASF1 and TONSL is the perfect example of histone chaperones collaborating to protect their cargo from spurious interactions. We hypothesized that additional histone co-chaperone partners of MCM2 and TONSL may exist and devised a proteomic screen to identify their histone-dependent interactors. We identified DNAJC9 as a co-chaperone of MCM2 and TONSL that can substitute for ASF1. Our structure-function characterization reveals that DNAJC9 has both heat shock co-chaperone and histone chaperone functionalities, integrating ATP-dependent protein remodeling enzymes into the histone chaperone network to safeguard histone H3-H4 dimer integrity.

RESULTS

The heat shock co-chaperone DNAJC9 functions as a histone chaperone

To identify potentially uncharacterized histone chaperones, we profiled the histone-dependent interactions of the histone chaperones MCM2 and TONSL by comparing the interactomes of their wild-type (WT) and histone binding mutant (HBM) forms in SILAC (stable isotope labeling with amino acids in cell culture) label swap co-immunoprecipitation experiments (Figures 1A–1C). For this purpose, we used the MCM2 Y81A Y90A and TONSL N571A mutants, which disrupt histone binding, as demonstrated previously (Huang et al., 2015; Saredi et al., 2016). Mass spectrometry analysis of these pull-downs confirmed the histone co-chaperone relationships between MCM2 and TONSL (Figure 1C) (Saredi et al., 2016), MCM2 and ASF1 (Groth et al., 2007), and between MCM2 and the FACT complex (SPT16/SP16H and SSRP1) (Foltman et al., 2013). TONSL also formed a histone co-chaperone complex with FACT (Figure 1C), demonstrating the histone dependence of this previously reported interaction (O'Connell et al., 2010). MCM3 and MCM5 showed a histone-dependent interaction with MCM2, suggesting that the histone-binding domain (HBD) of MCM2 stabilizes non-chromatin-bound MCM2–7 hexamers, which are otherwise salt labile (Fujita et al., 1997; Schulte et al., 1996). In addition, we identified DNAJC9 as a putative histone chaperone that formed co-chaperone interactions with both MCM2 and TONSL similar to ASF1A/B (Huang et al., 2015; Saredi et al., 2016) and FACT (Foltman et al., 2013) (Figures 1B and 1C).

DNAJC9 contains a J domain that acts as heat shock co-chaperone by binding and stimulating the ATP hydrolysis of HSP70-type enzymes (Han et al., 2007), similar to other HSP40/DnaJ family members (Mayer and Gierasch, 2019). The catalytic activities of HSP70-type enzymes, including HSP70 and HSC70, are henceforth referred to as "HSP70 catalysis." Although DNAJC9 was previously identified in soluble histone H3.1 purifications (Campos et al., 2010, 2015; Lambert et al., 2015), it remained unclear whether DNAJC9 binds histones directly or shows histone variant specificity. We found that DNAJC9 binds directly to H3.3-H4 and forms a co-chaperone complex with MCM2 using recombinant proteins (Figure 1D). Histone H3 variant pull-downs in cell extracts

CellPress
OPEN ACCESS

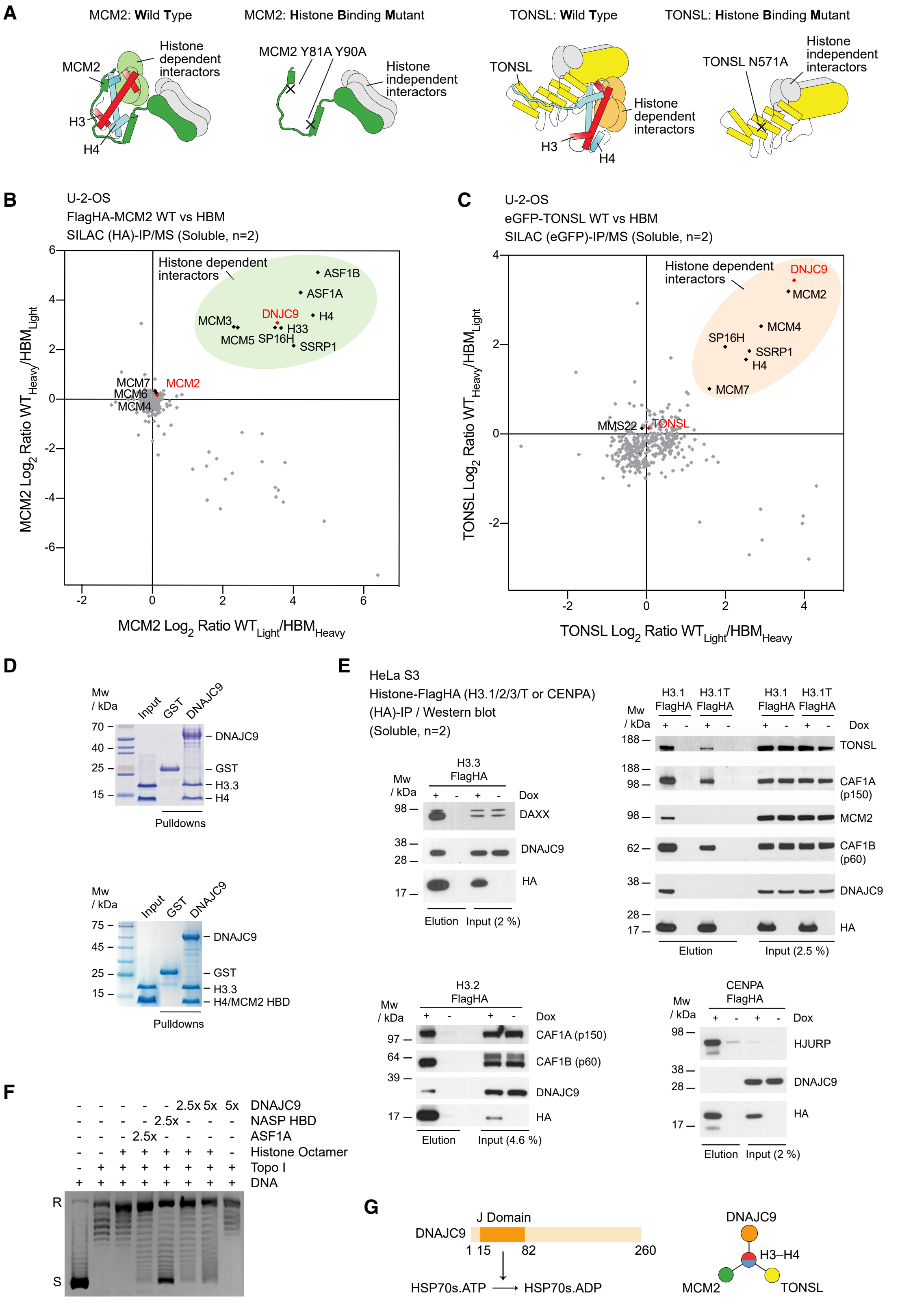

(legend on next page)

showed DNAJC9 associates with H3.1/2/3 but not centromeric CENPA or the testis-specific H3.1T variant (Figure 1E). Together, we conclude that DNAJC9 binds somatic non-centromeric histone H3 variants directly in a manner compatible with the co-association of histones with MCM2 and/or TONSL. We found that DNAJC9 could assemble histones onto relaxed circular plasmid DNA with efficiency comparable with ASF1A but weaker than NASP (Figure 1F). Together these results implicate DNAJC9 as a bona fide histone chaperone. Thus, DNAJC9 encodes the dual functionality of a histone chaperone and heat shock co-chaperone with the potential to direct the protein folding activities of HSP70 molecular chaperones (Clerico et al., 2019; Mayer and Gierasch, 2019; Rosenzweig et al., 2019) to histones H3 and H4 (Figure 1G).

Molecular basis of histone H3-H4 dimer recognition by DNAJC9

As DNAJC9 binds directly to histones H3 and H4 and forms a co-chaperone complex with MCM2 (Figure 1D), we set out to explore the molecular basis for recognition of histones H3 and H4 by DNAJC9. Our truncation analysis identified a HBD (amino acids 171–249) located in the C-terminal part of DNAJC9 (Figures 2A and 2B; Figure S1A). To prevent disulfide cross-linking during purification Cys243 of DNAJC9 was mutated to serine, which did not influence histone binding (Figure S1B). We obtained crystals of DNAJC9 HBD C243S in complex with histones H3.3 (57–135) and H4 as well as MCM2 HBD (61–130) and solved the structure at 2.50 Å resolution by molecular replacement using our previous structure of the MCM2 HBD-H3.3-H4 complex (Huang et al., 2015) (Figure 2C; X-ray statistics in Table 1). Two copies of DNAJC9 HBD-H3.3-H4-MCM2 HBD quaternary complex are found in an asymmetric unit, which are almost identical with a small root-mean-square deviation (RMSD) of 0.47 Å (Figure S1C). The structure shows that the HBDs of DNAJC9 and MCM2 co-chaperone an H3.3-H4 dimer (Figures 2C and 2D). MCM2 HBD wraps around the lateral DNA-binding interface of the H3.3-H4 dimer, as shown previously (Huang et al., 2015; Richet et al., 2015; Wang et al., 2015), while DNAJC9 HBD recognizes a relatively hydrophobic surface located mainly along the H3.3 α2 helix. DNAJC9 HBD adopts two α helices labeled αA and αB, each about 30 residues long, which are connected by a loop Lb. The αA helix of DNAJC9 HBD interacts with H4 L1 and H3.3 L2 loops, while the αB helix forms an antiparallel coiled coil-like structure with H3.3 α2 helix. Interestingly, the N-terminal half of the αB helix sterically prevents H3-H4 tetramerization, which otherwise forms within the MCM2 HBD-H3.3-H4 complex (Huang et al., 2015) and the nucleosome (Luger et al., 1997) (Figures S1D and S1E). The αB helix also forms a steric clash with ASF1A/B (English et al., 2006; Natsume et al., 2007), which implies that the binding of DNAJC9 and ASF1A/B to H3-H4 dimer are mutually exclusive (Figure 2E). The DNAJC9 αB helix would also obscure the binding site of the SPT2 αC1 helix, if it were to bind a H3-H4 dimer (Figure S1F). Note that the SPT2 HBD predominantly contacts a H3-H4 dimer in the context of an H3-H4 tetramer (Chen et al., 2015). Moreover, the C-terminal tail of H4 adopts a helical conformation following recognition by DNAJC9 HBD, distinct from the conformations observed in other chaperone-histone complexes (Elsässer et al., 2012; English et al., 2006) and at the nucleosome level (Luger et al., 1997) (Figure 2E; Figures S1D and S1E). This is consistent with the observations that the C-terminal tail of H4 can serve as a “handle” for recognition by histone chaperones (Elsässer et al., 2012; English et al., 2006).

DNAJC9 HBD has a few contacts with MCM2 HBD (Figure S1G) while having extensive interactions with H3.3 and H4. The residues Glu195 and Glu199 of the αA helix of DNAJC9 establish salt bridges with Arg45 and Lys44 in H4 L1 loop, respectively; Glu196 of αA forms hydrogen bonds to Val117 and Thr118 in H3.3 L2 loop; and Ala200 of αA situates in a shallow hydrophobic pocket lined by Lys115 and Val117 in H3.3 L2 loop and Ile112 in H3.3 α2 helix (Figures 3A and 3B). The residues Ala197 and Ser203 of αA, as well as Leu207 and Leu209 of the Lb loop of DNAJC9 further contribute hydrophobic interactions with H3.3 (Figure 3B). Moreover, the residues Gly212, Val213, Leu216, Ile220, and Gln224 of αB are bound in a consecutive channel constituted of Asp106, Leu109, Cys110, and His113 in H3.3 α2 helix; Ala127, Ile130, and Arg131 in H3.3 α3 helix; as well as Tyr98 in the H4 C terminus (Figure 3C). Notably, Arg223 and Arg227 of αB establish prominent polar interactions with Glu105 and Asp106 in H3.3 α2 helix (Figure 3C). Furthermore, the residues Met231, Phe234, Leu235, Met238, and Tyr242 of αB are bound into another consecutive channel constituted of Ala87, Ala91, Glu94, and Ala95 of H3.3 α2 helix, as well as Ala83, Met84, Val87, Leu90, and Lys91 of H4 α3 helix (Figure 3D). The residues Glu239 and Ser243 of αB also contribute some interactions with H4 α3 helix (Figure 3D). Notably, most of the interacting residues on DNAJC9 HBD are highly similar across eukaryotes (Figure 3A), implying conservation of DNAJC9 function as a histone chaperone.

Consistent with our structural observations, pull-down assays showed that a multiple mutant of αA (4A1: E195A E196A E199A

Figure 1. Identification of DNAJC9 as a dual histone chaperone and heat shock co-chaperone

(A) Schematic representations of the histone H3-H4 (red and cyan, respectively) binding mode of MCM2 (dark green) and TONSL (yellow) highlighting histone binding mutants (Huang et al., 2015; Saredi et al., 2016) and the experimental strategy for the identification of histone-dependent interactors. Hypothetical histone-dependent interactors (colored light green and orange for MCM2 and TONSL, respectively) and histone-independent interactors (gray) are depicted.

(B and C) Mass spectrometry analysis of SILAC labeled pull-downs of wild-type (WT) and histone binding mutant (HBM) forms of MCM2 (B) and TONSL (C) from soluble cell extracts; n = 2 biological replicates. Proteins referred to by human UniProt protein identification code. See also Table S1.

(D) Pull-downs of full-length GST-DNAJC9 mixed with pre-assembled H3.3-H4 (top) and MCM2 HBD-H3.3-H4 complexes (bottom).

(E) Pull-downs of FLAGHA-tagged histone H3 variants induced by doxycycline (Dox+) compared with control purifications (Dox−) from soluble cell extracts. Western blots representative of n = 2 biological replicates.

(F) Plasmid supercoiling assay showing histone chaperone activity of DNAJC9 compared with ASF1A and NASP HBD as positive controls. R, relaxed DNA; S, supercoiled DNA.

(G) Schematics depicting the ability of the DNAJC9 N-terminal J domain to stimulate HSP70 catalysis (Han et al., 2007) and the histone-dependent interactions formed between DNAJC9, MCM2, and TONSL.

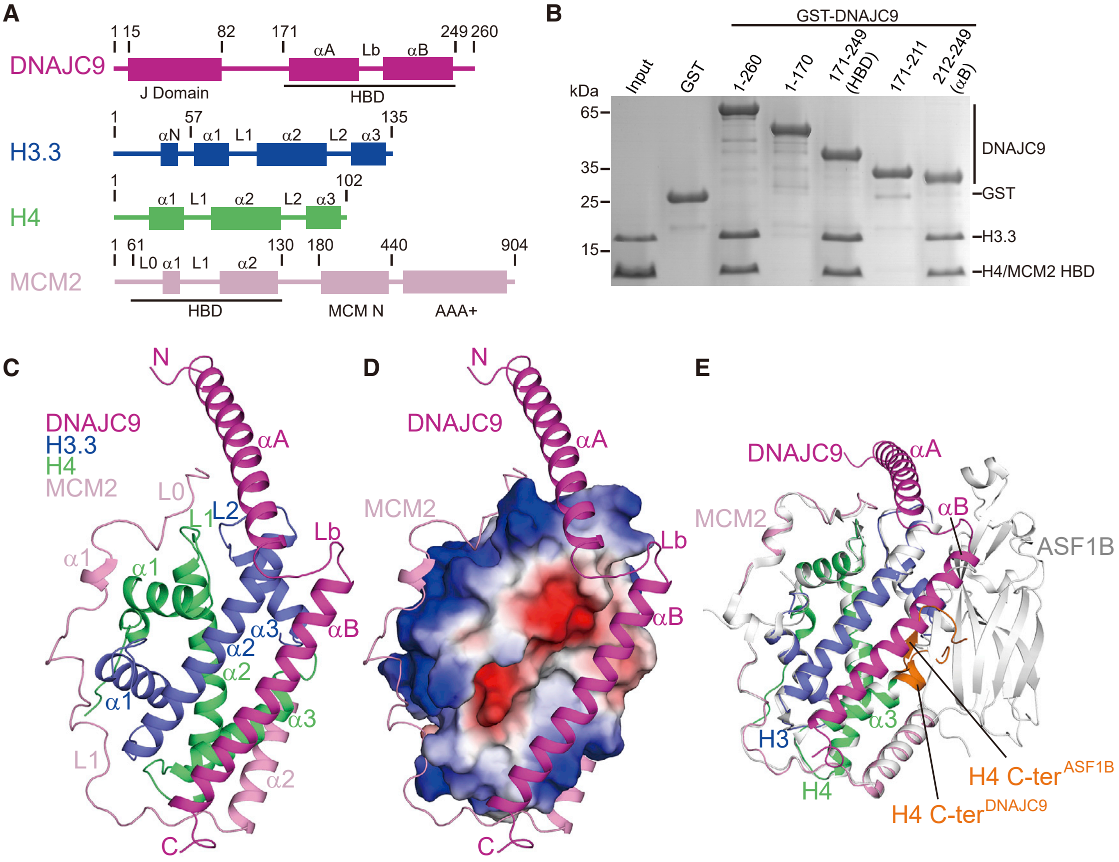

Figure 2. Structure of DNAJC9 and MCM2 bound simultaneously to an H3.3-H4 dimer

(A) Schematic domain architectures of DNAJC9, H3, H4, and MCM2.

(B) Pull-downs of GST-DNAJC9 constructs truncated to map the domain of interaction with pre-assembled MCM2 HBD-H3.3-H4 complexes. See Figure S1A for analogous GST-DNAJC9 pull-downs of H3.3-H4 complexes.

(C) Structure of the DNAJC9 HBD-H3.3-H4-MCM2 HBD quaternary complex, with DNAJC9 HBD colored in magenta, H3.3 in blue, H4 in green, and MCM2 HBD in pink. See also Table 1 and Figure S1.

(D) DNAJC9 HBD (magenta) and MCM2 HBD (pink) wrapping around the H3.3-H4 dimer in surface view colored according to electrostatic potential (red, negatively charged; blue, positively charged).

(E) Structural comparison between DNAJC9 HBD-H3.3-H4-MCM2 HBD (colored as in C) and MCM2 HBD-H3.3-H4-ASF1B (silver; PDB: 5BNX). The αB helix of DNAJC9 HBD forms a steric clash with ASF1B. The H4 C terminus ("C-ter"; orange) adopts a helical conformation upon DNAJC9 HBD binding, while it forms a β strand with ASF1B.

See also Figure S1.

A200E) and three separate double mutants of αB (Q224A R227A; F234A L235A; M238A Y242A) reduced the binding of DNAJC9 HBD to the MCM2 HBD-H3.3-H4 complex, while the αB double mutant L216A I220A and the multiple "4A" mutant (Q224A R227A M238A Y242A) almost totally disrupted the interactions (Figure 3E). Our isothermal titration calorimetry (ITC) results confirmed that the 4A mutant of DNAJC9 HBD disrupted binding to H3.3-H4 and further showed that DNAJC9 HBD had similar binding affinities for variant H3.3-H4 and canonical H3.1-H4 dimers (K_d = 55.0 ± 19.7 and 39.5 ± 4.9 nM, respectively; Figure 3F). Thus, the contact of DNAJC9 HBD with H3.3 Ala87 (Ser87 in H3.1 and H3.2) does not distinguish these H3 variants (Figure 3D).

Both the structural and biochemical results support the concept that the αB helix of DNAJC9 HBD is the main determinant of H3.3-H4 recognition (Figure 3). Indeed, the isolated DNAJC9 αB helix (C243S), observed in a separate 1.80 Å resolution crystal structure (Figure S1H; X-ray statistics in Table 1), occupied the same binding site as in the DNAJC9 HBD-H3.3-H4-MCM2 HBD structure (Figure 2C). Interestingly, DNAJC9 HBD αB has a similar structural configuration to the αA helix of CENPA-specific chaperone HJURP (Hu et al., 2011) (Figure S1I), though they share no significant sequence similarity. Taken together, we have unraveled the molecular basis for recognition of a histone H3-H4 dimer by the novel histone chaperone DNAJC9.

DNAJC9 recruits HSP70 catalysis to fold H3-H4 substrates

To place DNAJC9 in the histone supply pathway, we determined the histone-dependence of DNAJC9's interactome using our SILAC label swap co-immunoprecipitation strategy. Consistent

Table 1. Data collection and refinement statistics

	DNAJC9 HBD-H3.3-H4-MCM2 HBD (7CJ0)	DNAJC9 αB-H3.3-H4-MCM2 HBD (7CIZ)
Data collection		
Space group	$P3_121$	$C222_1$
Cell dimensions		
a, *b*, *c* (Å)	47.5, 47.5, 616.3	47.6, 176.8, 202.9
α, β, γ (°)	90, 90, 120	90, 90, 90
Resolution (Å)	41.08–2.24 (2.30–2.24)[a]	40–1.80 (1.83–1.80)[a]
No. reflections (total/unique)	716,208/41,413	781,980/78,310
R_{pim} (%)	2.2 (51.9)	2.4 (40.0)
$I/\sigma I$	17.7 (1.9)	31.3 (2.2)
Completeness (%)	99.9 (99.1)	97.7 (91.2)
Redundancy	17.3 (16.8)	10.0 (9.5)
Refinement		
Resolution (Å)	41.08–2.50	36.88–1.80
No. reflections (unique)	29,934	77,571
R_{work}/R_{free} (%)	25.8/29.5[b]	17.1/21.4
No. atoms		
Protein	4,621	5,956
GOL	6	
SO_4		10
Water	69	741
B-factors		
Protein	79.4	24.0
GOL	82.1	
SO_4		62.7
Water	69.0	38.9
RMSDs		
Bond lengths (Å)	0.002	0.006
Bond angles (°)	0.45	0.79

Related to Figures 2 and S1.
[a]Values in parentheses are for highest resolution shell. One crystal was used for each dataset.
[b]The dataset of 7CJ0 was twined, and a merohedral twin law (-h, -k, l) was applied only for the final round of refinement in PHENIX.

with our *in vitro* analysis (Figure 3E), the DNAJC9 mutants (Q224A R227A and M238A Y242A) showed partial loss of histone binding and, as a consequence, reduced MCM2 binding in cell extracts (Figure S2A). We thus used the stronger combined mutant DNAJC9 4A (Q224A R227A M238A Y242A) for further analysis (Figure 3E). Comparison of DNAJC9 WT and 4A mutant interactomes confirmed the loss of histones, MCM2 and TONSL in the 4A mutant (Figure 4A; Figure S2B). In addition, we identified specific interactions with proteins of the heat shock molecular chaperone machinery (HSP7C, HS71B, and BAG2), and intriguingly, part of these were histone dependent (HSP7C, BAG2) (Figure 4A). To understand the role of DNAJC9 in heat shock factor recruitment to histone substrates, we determined the effect of DNAJC9, HSP7C and BAG2 depletion on the interactomes of soluble H3.1 and H4 (Figure 4B; Figure S3). Strikingly, multiple HSP70 enzymes (HSP7C, HS71B, HSP72, and HSP74) and nucleotide exchange factors (BAG2 and HS105) were dependent on DNAJC9 for H3.1 and H4 binding (Figure 4B; Figures S3A–S3C, S3H, and S3I). Loss of HSP7C was accompanied by a gain of histone binding to other HSP70-type enzymes, HS71B and HSP72 (Figure 4B; Figures S3D, S3E, S3H, and S3I), probably reflecting compensation for HSP7C loss, in line with previous studies (Powers et al., 2008). Meanwhile, DNAJC9 showed no dependency on HSP7C or BAG2 for histone binding (Figure 4B; Figures S3D–S3I). Collectively, this supports a role of DNAJC9 in the recruitment of HSP70-type molecular chaperone machinery to histone H3-H4 substrates.

Depletion of DNAJC9 also significantly reduced the histone load of histone chaperones SPT2, TONSL, and MCM2 (Figure 4B). Meanwhile, histones H3.1-H4 accumulated with the FACT complex (SPT16 and SSRP1) and, surprisingly, the histone H3.3-specific chaperone DAXX (Elsässer et al., 2012; Lewis et al., 2010) (Figure 4B; Figure S3B). The latter was not accompanied by a gain of H3.3-specific peptides (Figure S3J). This suggests that failure to recruit the heat shock molecular chaperone machinery to H3.1-H4 perturbs the histone supply chain and leads to aberrant accumulation of H3.1-H4 with DAXX, likely in a misfolded state, thus

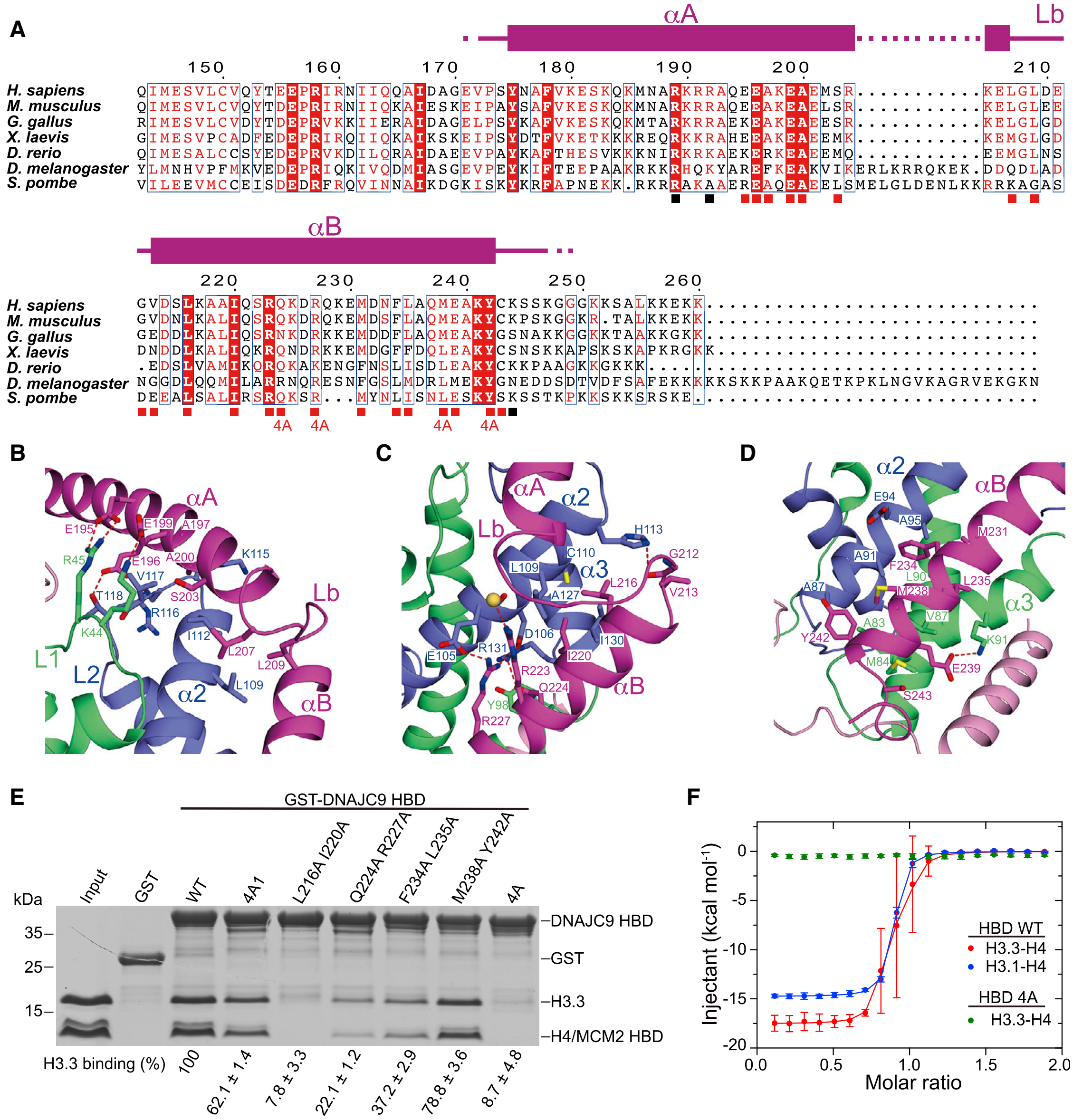

Figure 3. Molecular basis for recognition of H3.3-H4 by DNAJC9

(A) Multiple sequence alignment of DNAJC9 HBD: *H. sapiens* (NP_056005), *M. musculus* (NP_598842), *G. gallus* (NP_001186454), *X. laevis* (NP_001089275), *D. rerio* (NP_001002433), *D. melanogaster* (NP_001262473), and *S. pombe* (NP_594359). Under the alignment, red squares indicate residues of DNAJC9 interacting with H3.3-H4; "4A" highlights the multiple mutant disrupting interaction with H3.3-H4; black squares indicate residues of DNAJC9 HBD interacting with MCM2 HBD.

(B–D) Enlarged views showing the interaction details between DNAJC9 HBD (magenta) and H3.3-H4 (blue and green, respectively).

(E) Effects of DNAJC9 HBD mutants on histone binding using GST pull-downs. 4A1 and 4A corresponding to mutants "E195A E196A E199A A200E" and "Q224A R227A M238A Y242A," respectively. Quantifications on H3.3 based on replicate experiments (n = 3), expressed as mean ± SEM percentage. The signal of H3.3 in WT lane from the same gel was set to 100%.

(F) ITC results of DNAJC9 HBD WT and 4A mutant with histones (n = 3 independent experiments, error bars represent mean ± SD). DNAJC9 HBD WT binds H3.3-H4 with a K_d of 55.0 ± 19.7 nM and H3.1-H4 with a K_d of 39.5 ± 4.9 nM. K_d values represent the mean ± SD of independent measurements (n = 3). No binding was observed between DNAJC9 HBD 4A mutant and H3.3-H4 (n = 3).

accounting for the loss of histone variant specificity (DeNizio et al., 2014). To test whether DNAJC9 function is required for histone entry into the supply chain, we introduced two mutations in H3.1, E105A D106A (ED105AA), at sites conserved in H3.2 and H3.3 and structurally predicted to disrupt binding to DNAJC9 (Figure 3C). Proteomic comparison of soluble histone H3.1 interactomes showed a loss of DNAJC9 in H3.1 ED105AA along with several histone chaperones key to H3.1-H4 provision, including NASP, ASF1A/B, MCM2, and CAF-1 (Figure 4C), consistent with a defect upstream in the H3.1 supply pathway. Surprisingly, the

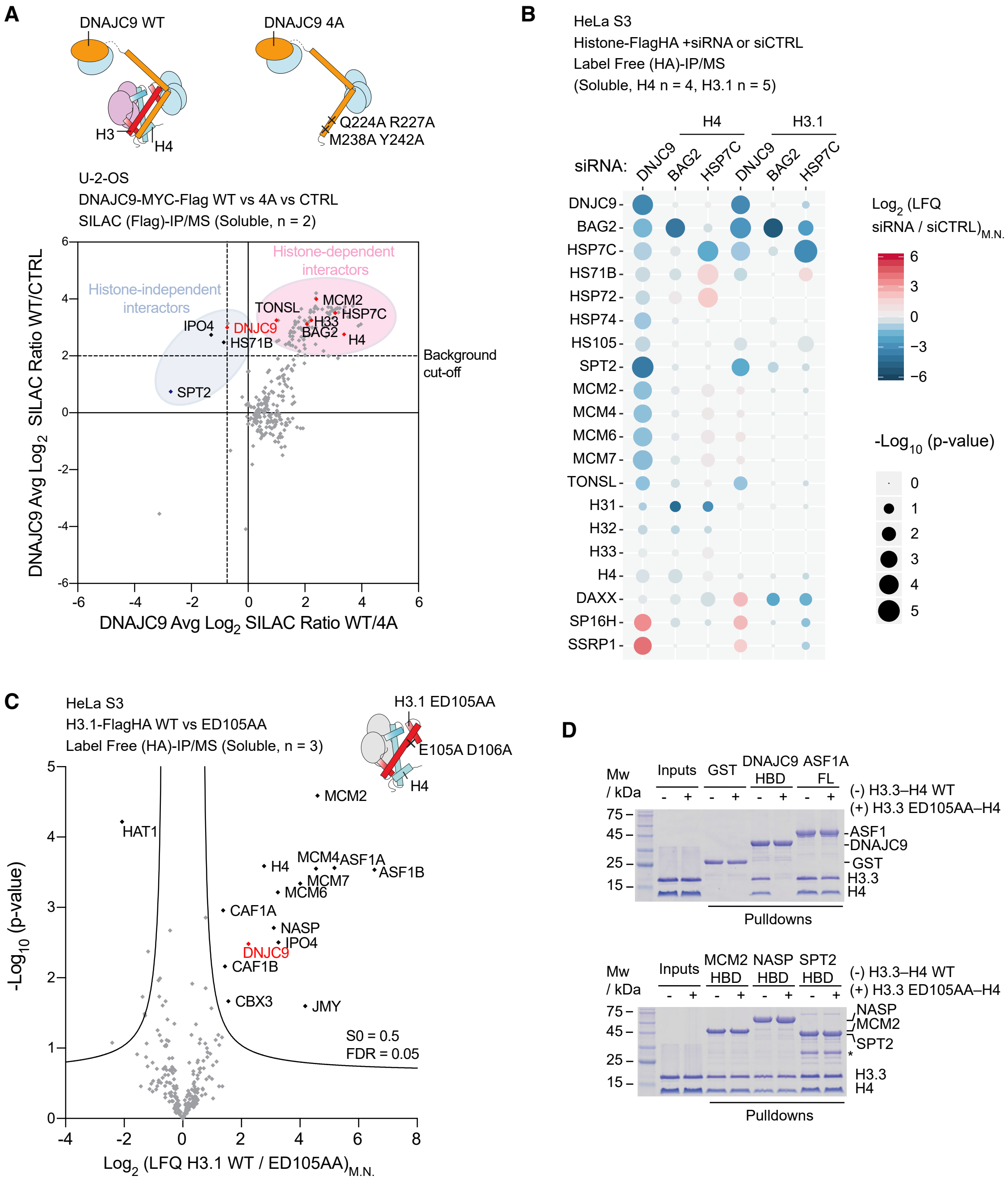

Figure 4. DNAJC9 recruits the heat shock molecular chaperone machinery to fold histone H3-H4 substrates

(A) DNAJC9 WT, 4A mutant, and control purifications subjected to triple SILAC-based mass spectrometry analysis. Ratios averaged from n = 2 biological replicates.

(B) Histone purifications from soluble extracts of cells small interfering RNA (siRNA) depleted for DNAJC9, BAG2, or HSC7C compared with control (CTRL) siRNA and analyzed using label-free mass spectrometry (s0 = 0.5, false discovery rate [FDR] = 0.05, H3.1 n = 5 and H4 n = 4 biological replicates). Bubble plots colors represent Log_2 ratios of median-normalized LFQ intensities $(siRNA/siCTRL)_{M.N.}$, and radii represent significance of changes (s0 = 0.5, FDR = 0.05); no imputed values shown.

(C) Histone purifications from soluble extracts subjected to label-free mass spectrometry analysis (n = 3 biological replicates, s0 = 0.5, FDR = 0.05). Volcano plots represent differences in median-normalized LFQ intensities ($LFQ_{M.N.}$) with missing values imputed for factors observed three times in either replicate.

(D) GST pull-down assays showing H3.3 WT- or H3.3 ED105AA-H4 binding to selected histone chaperones.

In (A)–(C), proteins are referred to by human UniProt protein identification code.

See also Figures S2 and S3 and Table S1.

CellPress
OPEN ACCESS

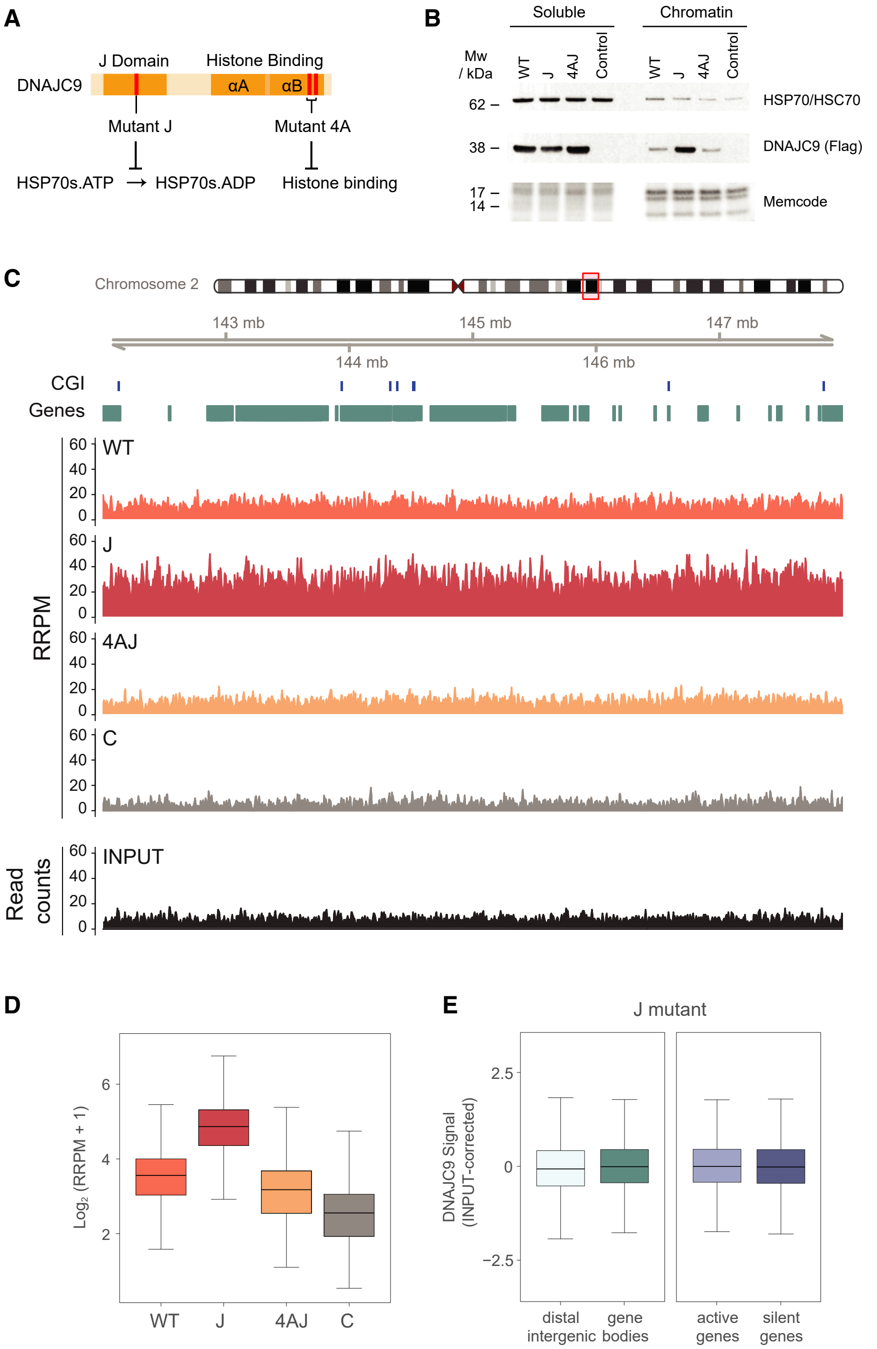

Figure 5. J domain mutation traps DNAJC9 on chromatin genome-wide in a histone-dependent manner

(A) DNAJC9 domain map with relevant mutations.

(B) Western blots of soluble and chromatin extracts from cells expressing DNAJC9-MYC-FLAG WT, J, or 4AJ mutants compared with control cells. See also Figure S4.

(C–E) Quantitative ChIP-seq of cells expressing DNAJC9-MYC-FLAG WT, J, or 4AJ mutants compared with control cells. ChIP-seq reads were quantitated in 10 kb windows with a 5 kb step. Plots represent data averaged from n = 2 biological replicates.

(C) Visualization of spike-in normalized ChIP-seq signal in DNAJC9 WT, J, 4AJ, and control samples, quantitated with reference-adjusted reads per million (RRPM), and raw input reads over the region depicted.

(D) Boxplots of spike-in normalized DNAJC9 ChIP-seq signal across the genome quantitated with reference-adjusted reads per million (RRPM), $Log_2(n + 1)$. Black line, median; whiskers, 1.5 × interquartile range.

(E) Boxplots of input corrected signal for DNAJC9 J mutant over gene bodies and intergenic regions (left) or gene bodies parsed to active and inactive genes (right). Black line, median; whiskers, 1.5 × interquartile range.

H3.1 ED105AA mutant also showed impaired association with histone H4 (Figure 4C). The histone heterodimerization defect of the mutant was not intrinsic, as we were able to reconstitute the H3.3 ED105AA-H4 complex *in vitro* (Figure 4D). Consistent with our structural data, the H3.3 ED105AA mutation abrogated binding to DNAJC9 *in vitro*, but this mutation did not compromise interactions with the other histone chaperones ASF1A, MCM2, NASP, and SPT2 in direct binding assays (Figure 4D). Therefore, the cellular defect in H3.1 ED105AA-H4 heterodimerization is attributed to the loss of DNAJC9 binding, which prevents histones from entering the histone chaperone supply pathway. Accordingly, failure of the H3.1T variant to bind DNAJC9 (Figure 1E) was also accompanied by a loss of histone H4, ASF1A/B, and CAF-1 as well as heat shock factors (Figure S2C). Together, this argues that DNAJC9-dependent recruitment of the heat shock molecular chaperone machinery is a requirement for H3-H4 heterodimer formation *in vivo*.

Loss of J domain function traps histone-bound DNAJC9 on chromatin genome-wide

To further dissect the requirement for DNAJC9-directed HSP70 catalysis (Han et al., 2007), we targeted the HPD motif of DNAJC9, a conserved motif in J domain proteins known to bind and stimulate ATP hydrolysis of HSP70s (Mayer and Gierasch, 2019). We introduced mutations that abolish these activities (H43Q and D45N) (Suh et al., 1998; Wall et al., 1994) in an otherwise WT or HBM (4A) background, forming mutants J and 4AJ, respectively (Figure 5A). Because of the catalytic dead nature of the DNAJC9 J mutant, we predicted that DNAJC9 would accumulate potentially misfolded histones if HSP70 activity is required for their re-folding and handover to other histone chaperones. Through cellular fractionation experiments, we found that a proportion of the DNAJC9 J

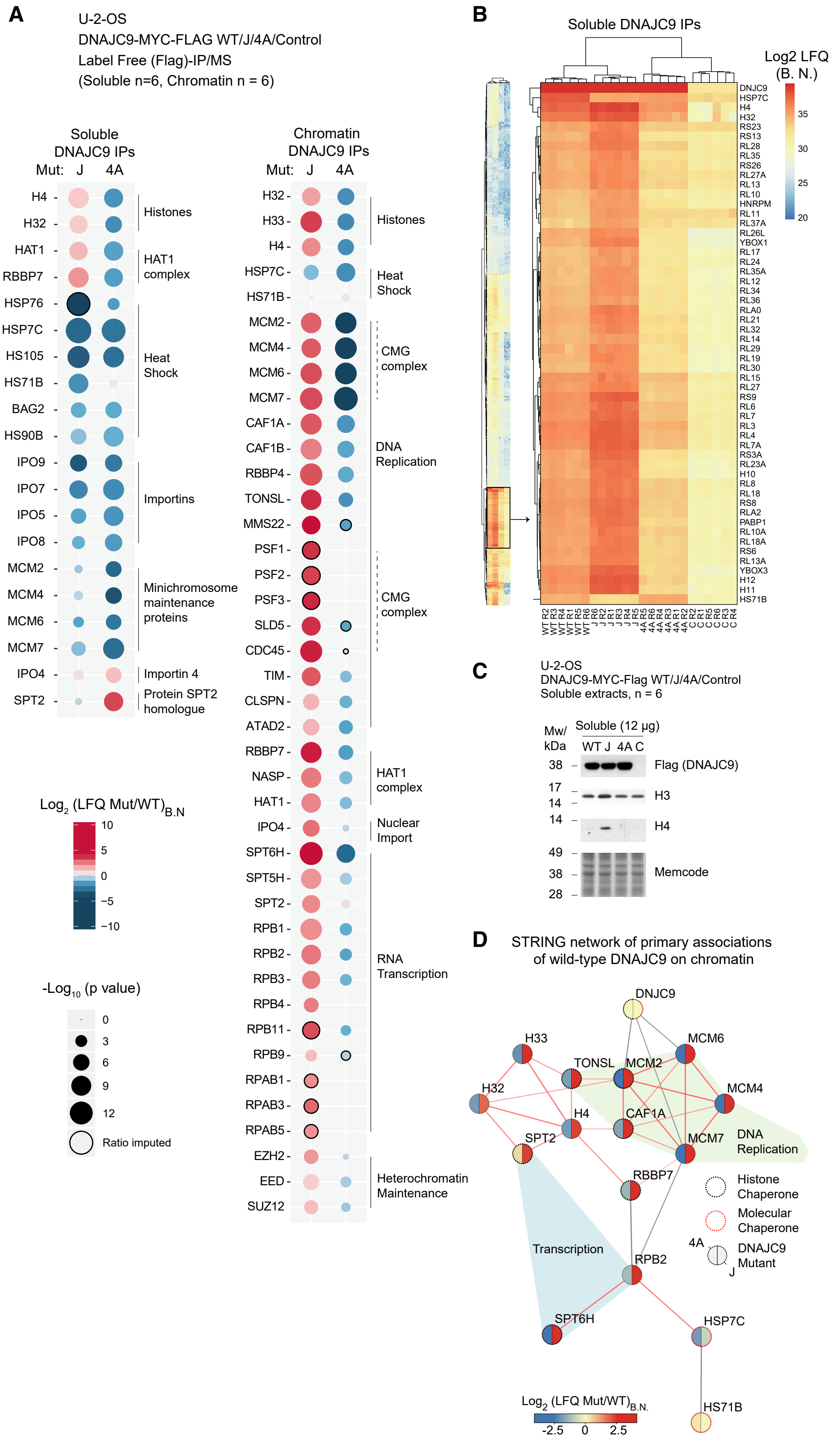

(legend on next page)

CellPress
OPEN ACCESS

mutant becomes trapped on chromatin in a manner dependent on histone binding (Figure 5B). This was confirmed by immunofluorescence analysis in cells pre-extracted to remove soluble non-chromatin-bound proteins (Figures S4A–S4C). The J mutant was bound to chromatin throughout the cell cycle (Figure S4D), moderately increasing as cells progress from G1 to G2/M phase (Figure S4E). However, none of the DNAJC9 mutants showed dominant effects on DNA replication or cell cycle progression (Figures S4F and S4G). To identify potential hotspots for DNAJC9 function, we performed quantitative chromatin immunoprecipitation sequencing (ChIP-seq) to localize the genomic occupancy of the DNAJC9 WT, J, and 4AJ mutants (Figures 5C–5E). Spike-in normalized read counts confirmed histone-dependent trapping of the DNAJC9 J mutant (Figures 5C and 5D). However, the J mutant showed a genome-wide occupancy pattern with similarity to pan histone H3 (Reverón-Gómez et al., 2018), lacking enrichment of specific genomic features (Figures 5C and 5E). This argues that when DNAJC9 cannot direct HSP70 molecular chaperone activity toward its histone H3-H4 cargo, it becomes trapped on chromatin through widespread spurious interactions of misfolded histones with DNA.

DNAJC9 safeguards histones during supply to and transactions within chromatin

To address the contexts in which histone substrates require DNAJC9-mediated HSP70 folding activities, we compared DNAJC9 WT, J, and 4A interactomes in both soluble and chromatin fractions (Figure 6A; Figures S5 and S6). The J mutant lost interaction with the heat shock molecular chaperone machinery as expected, and concomitantly histone H3-H4 accumulated with the mutant in both fractions (Figure 6A). This demonstrates that HSP70 catalysis is required for the release of histones from DNAJC9. In the soluble fraction, the J mutant trapped a subset of histone-dependent interactors, including the HAT1 complex (HAT1-RbAp46/RBBP7) and an abundance of ribosomal and RNA binding proteins (Figures 6A and 6B). Similarly, HAT1 accumulated with the histone H3.1 ED105AA mutant defective in DNAJC9 binding and H3-H4 heterodimerization (Figure 4C). However, we did not detect trapping of other downstream histone chaperones with the J mutant in the soluble fraction (e.g., MCM2, ASF1A/B, NASP, and FACT). This is consistent with misfolded histones failing to enter the assembly line in the absence of proper DNAJC9 function; instead, they become stalled in complex with HAT1 and interact with soluble RNAs non-specifically. In accordance with this conclusion, expression of the J mutant triggered accumulation of histones H3 and H4 in the soluble fraction (Figure 6C).

In the chromatin fraction, abrogation of HSP70 catalysis caused a dramatic increase in DNAJC9 interactors (34 WT versus 404 J mutant), consistent with widespread spurious interactions with chromatin (Figure S6B; Table S1). Histone deposition factors linked to DNA replication (including MCM2, TONSL, and the CAF-1 complex) and transcription (including SPT2 and SPT6) were among the most significantly trapped factors by the J mutant (Figure 6A; Figures S6E and S6G). Moreover, the active CMG helicase and RNA polymerase components also co-purified with the J mutant (Figure 6A), arguing that DNAJC9 directs HSP70 catalysis to aid folding/release of histones from chaperones engaged in active replication and transcription. Importantly, the same histone chaperones (MCM2, TONSL, CAF-1, SPT2, and SPT6) in addition to heat shock factors (HSP7C and HS71B) were among the most highly enriched factors in DNAJC9 WT chromatin purifications (Figure 6D). Collectively, this argues that DNAJC9 facilitates histone H3-H4 deposition through the recruitment of HSP70s to release histones trapped in aberrant intermediates with histone deposition factors on chromatin (CAF-1, MCM2, SPT2, and SPT6). This would allow misfolded histones to be scavenged and recycled into the deposition pathways (Figure 7).

DISCUSSION

We show that DNAJC9 integrates heat shock molecular chaperone activity into the network of histone chaperones, promoting the entry/re-entry of misfolded and trapped histones into productive histone supply chains and deposition intermediates. Our data support the notion that DNAJC9 acts in parallel to classical histone chaperones, recruiting heat shock factors to protect the structural integrity of histone H3-H4 dimers during the most challenging stages of their supply and deposition. This appears to be particularly important shortly after histone synthesis and also later at sites of deposition in chromatin, presumably to release histones from non-specific interactions with ribosomal RNA and DNA, respectively (Figure 7). Histones folded/re-folded through DNAJC9 re-enter the histone supply chain upstream of HAT1 or at sites of active histone deposition linked to replication (CAF-1, MCM2, and TONSL) and transcription (SPT2 and SPT6) (Figure 7).

Figure 6. DNAJC9-directed HSP70 activity facilitates histone supply to and transactions within chromatin

(A–D) DNAJC9 WT, mutant, and control purifications from soluble and chromatin extracts subjected to label-free mass spectrometry analysis (n = 6 biological replicates). Proteins are referred to by human UniProt protein identification code. See also Figures S5 and S6 and Table S1.

(A) Bubble plots from (left) soluble and (right) chromatin fraction purifications showing enrichment (red) and depletion (blue) in J domain and histone binding mutants (J and 4A, respectively) compared with WT. Ratios calculated from bait-normalized LFQ intensities ($LFQ_{B.N.}$). Data analysis steps are detailed in Figures S5 and S6.

(B) Left: Euclidean clustering analysis of soluble fraction $LFQ_{B.N.}$ intensities for factors identified in at least six of six experiments for WT, J, or 4A mutants and additionally significantly enriched in at least one set over the control purifications (S0 = 2, FDR = 0.01). Region of interest highlighted (black box) and magnified (right) to show trends of enrichment and depletion of histones, ribosomal proteins, HSP70s and other factors.

(C) Western blots of soluble extracts from cells expressing DNAJC9 WT, J, or 4A mutants and control cells showing the accumulation of soluble histones in cells expressing the DNAJC9 J mutant.

(D) STRING-db network of factors most significantly enriched in DNAJC9 WT purifications and overlaid ratios of enrichment/depletion in J and 4A mutants. Sixteen of 22 nodes with $Log_2 (LFQ\ WT/C)_{B.N.} > 1.8$ were connected to each other at a STRING confidence level of 0.6; red edges represent nodes connected with experimental evidence.

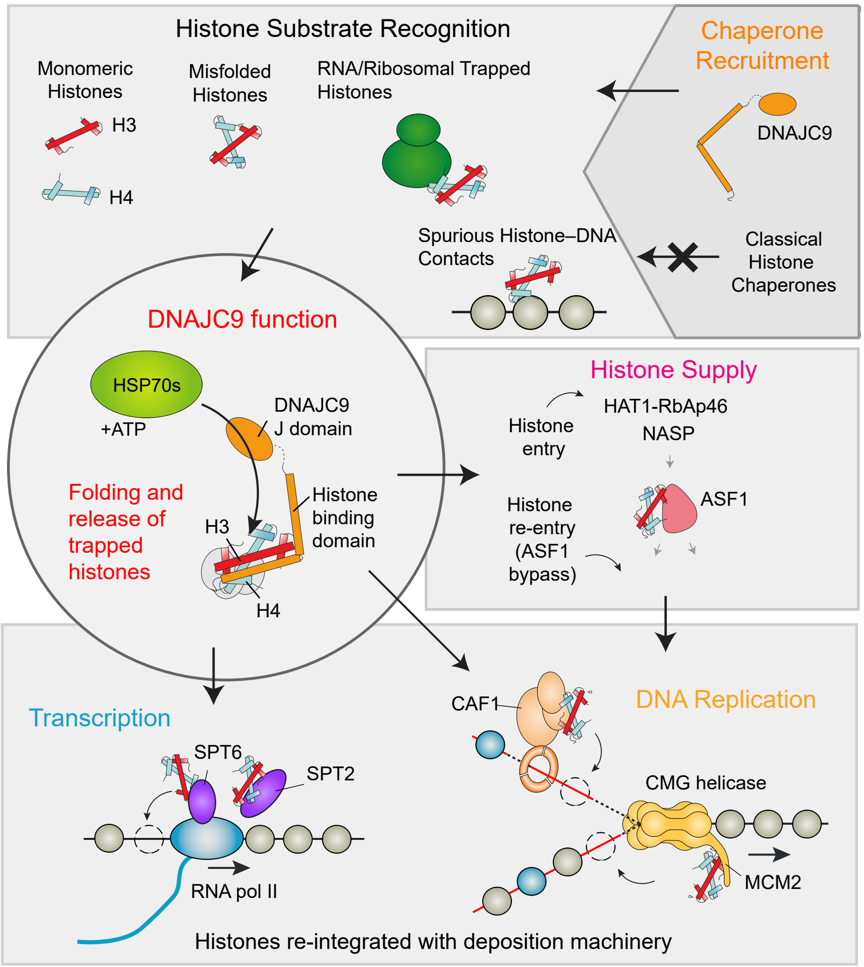

Figure 7. DNAJC9 links heat shock biology to the histone chaperone network

DNAJC9 binds histone substrates that cannot engage other histone chaperones because of being monomeric, misfolded, or engaged in spurious interactions with RNA/DNA. DNAJC9 recruits HSP70-type enzymes through its J domain to fold and release of histones substrates with ATP-derived energy. DNAJC9-bound histones can enter the histone chaperone supply chain upstream of HAT1 for their eventual delivery to chromatin by ASF1. Alternatively, DNAJC9-bound histone dimers bypass ASF1 and engage with histone deposition chaperones during DNA replication and transcription.

Our structure demonstrates DNAJC9 bound to a fully folded H3-H4 dimer, which is likely a snapshot of the catalytic reaction and foreseeably the endpoint of the HSP70 catalytic cycle captured with pre-folded histones. In this conformation, histones would be able to re-engage other histone chaperones, as also demonstrated by the co-crystallization of DNAJC9 bound histones with MCM2. We show through structural analysis that DNAJC9 has a competitive binding mode with ASF1 and SPT2, yet we see an intriguing interplay with both histone chaperones. DNAJC9 supplies histones to SPT2, and it forms a histone-independent interaction with SPT2 that can be trapped on chromatin by the J domain mutant. These observations point toward a histone handover event between DNAJC9 and SPT2 that requires HSP70 catalysis. In contrast, our functional proteomics data support a mutually exclusive relationship between DNAJC9 with ASF1, which is intriguing given the overlap of their histone co-chaperone partners, including MCM2, TONSL, and CAF-1. This suggests that DNAJC9 provides cells with a route to bypass ASF1 centered histone H3-H4 supply pathways to ultimately engage with same deposition machinery. This may be particularly useful for retaining histones that would otherwise drop out of the supply chain because of misfolding or non-specific associations with RNA/DNA.

Our data would support two DNAJC9-dependent entry points for histones into the HSP70 catalytic cycle (Figure 7), whereby either monomeric histones or trapped/misfolded H3-H4 dimers enter. Dependent on the entry point and cellular context, DNAJC9 may then engage one or more HSP70-type enzymes to release fully folded histone substrates into productive supply and deposition complexes. We find that DNAJC9 promotes the heterodimerization of H3-H4 prior to engaging NASP and other histone chaperones. Consistent with this, DNAJC9 forms histone-dependent and histone-independent interactions with HSC70 and HSP70 (HSP7C, HS71B), which have been observed in separate complexes with monomeric histones H3.1 and H4, respectively (Alvarez et al., 2011; Campos et al., 2010). Thus, DNAJC9 has the combined attributes required to catalyze *de novo* H3-H4 heterodimerization.

Perhaps our most surprising observation is that the histone folding functionality of DNAJC9 is used not only at the beginning of their soluble life but also at the end, when histones are deposited on chromatin. This demonstrates that a histone chaperone's function is not necessarily staged in the backdrop of a linear assembly pathway. Rather, histone chaperones opportunistically bind histones based on compatibilities with histone conformations and histone co-chaperone partners that can occur throughout supply if the cargo and subcellular localization are right. Indeed, DNAJC9 probably facilitates many histone transactions that place a particular strain on the stability of the H3-H4 fold. This would include during the release of histones from non-productive high-affinity intermediates with histone chaperones and nucleic acids (Figure 7). In this capacity, DNAJC9 provides a safeguard mechanism ensuring the structural integrity of histone H3-H4 dimers. The fact that the histone heterodimer is somewhat malleable in certain cellular contexts provides interesting possibilities to mediate histone handover events on the basis of histone conformational dynamics, as alluded to previously (Zhang et al., 2013). Thus, chaperone availability and

CellPress
OPEN ACCESS

histone conformation have great potential to affect chromatin states through the selective channeling of histones into competing deposition pathways, as demonstrated by the re-wiring of DAXX to bind H3.1 in the absence of DNAJC9.

Together, our work identifies a continuous requirement for heat shock molecular chaperoning of histones, including during biosynthesis, replication, and transcription, met by the dual functionality of DNAJC9. This adds another layer to the multifaceted controls ensuring efficient histone supply, which also include elaborate transcriptional and translational regulation (Marzluff and Koreski, 2017; Mendiratta et al., 2019). Although histone co-chaperone complexes provide the means to shield multiple histone functional surfaces (Hammond et al., 2017), it appears that passive shielding capacity alone is insufficient to fully protect histones H3-H4. Rather, cells direct ATP-derived energy and HSP70 enzymes to refold histones to facilitate their supply to and transactions within chromatin (Figure 7). The term "molecular chaperone" was first used to describe a protein with the ability to prevent the precipitation of histones when mixed with DNA under physiological salt conditions (Laskey et al., 1978). Since this initial discovery, "molecular chaperone" terminology has been extended to heat shock protein functionality (Ellis, 1987), and the original definition is now used to define a "histone chaperone." DNAJC9 integrates both histone chaperone and molecular chaperone functionalities in the modern sense of these terms.

Limitations of study

Our structural analysis reports on the conformation by which DNAJC9 binds a folded histone H3-H4 dimer. We believe this to be a snapshot of a catalytic folding mechanism yet to be fully elucidated. Time-resolved biochemical and structural analysis of the DNAJC9-stimulated HSP70 catalytic folding mechanism of histones H3-H4 in a reconstituted system would provide exceptional mechanistic insights to DNAJC9's mode of action. However, whether DNAJC9 acts to fold or re-fold histones will depend largely on the biological context of the substrate and the molecular chaperones engaged. In a cellular context, if a histone substrate engages DNAJC9 but cannot complete the HSP70 catalysis cycle of choice, this might trigger a response to route histones toward degradation. We show that DNAJC9 engages histones at multiple stages of histone supply, including shortly after histone biosynthesis and during transactions with histone deposition chaperones. DNAJC9 can hereby support the H3-H4 supply chain allowing histones to enter and re-enter this pathway at multiple points, supporting the ASF1 centered H3-H4 supply chain. DNAJC9 has been identified by CRISPR screens as an essential gene across a large number of cancer cell lines as reported in the DepMap portal (https://depmap.org/portal) and elsewhere (Behan et al., 2019; Dwane et al., 2021; Hart et al., 2015; Zhang et al., 2020). In light of the multiple ways in which DNAJC9 links to histone metabolism, it will be important to resolve which aspects of DNAJC9 function are critically important for cell survival.

STAR★METHODS

Detailed methods are provided in the online version of this paper and include the following:

- KEY RESOURCES TABLE
- RESOURCE AVAILABILITY
  - Lead contact
  - Materials availability
  - Data and code availability
- EXPERIMENTAL MODEL AND SUBJECT DETAILS
  - Cell lines
- METHOD DETAILS
  - Plasmid construction
  - Cloning and protein preparation in bacteria
  - Crystallization, X-ray data collection and structure determination
  - GST pulldowns
  - Isothermal titration calorimetry (ITC)
  - Plasmid supercoiling assay
  - Cell extracts
  - Immunoprecipitation
  - Quantitative ChIP-seq and cell fractionation
  - DNA sequencing, data processing and analysis
  - Immunofluorescence microscopy
  - Antibodies
  - MS sample preparation
  - MS analysis
- QUANTIFICATION AND STATISTICAL ANALYSIS
  - Analysis of MS data
  - Statistical analysis of MS data
  - Data visualization

SUPPLEMENTAL INFORMATION

Supplemental information can be found online at https://doi.org/10.1016/j.molcel.2021.03.041.

ACKNOWLEDGMENTS

We thank Caroline B. Strømme for supervising A.G.-N., Simone Schopper for help with proteomic analysis, Manuel Garcia Albornoz for sharing R scripts, Alva Biran for R programming support, and all Groth lab members for discussions. We thank the staff from beamlines BL17U1, 18U1, and 19U1 at the Shanghai Synchrotron Radiation Facility (SSRF) for assistance during data collection. H.B. is partially supported by the National Key R&D Program of China (2018YFC1004500) and the Chinese National Natural Science Foundation (31800619). Research in the Rappsilber lab is funded by the Wellcome Trust through a Senior Research Fellowship to J.R. (103139). The Wellcome Centre for Cell Biology is supported by core funding from the Wellcome Trust (203149). Research in the Nielsen lab is supported by the Independent Research Fund Denmark (0135-00096B and 8020-00220B), the European Union's Horizon 2020 research and innovation program under grant agreement EPIC-XS-823839, and the Danish Cancer Society (R146-A9159-16-S2). Research in the Patel lab is funded by a Leukemia and Lymphoma Society SCOR grant (7021-20) and by the Memorial Sloan Kettering Core Grant (P30CA008748). Research in the Huang lab is supported by the National Key R&D Program of China (2018YFC1004500); the Shenzhen Science and Technology Program (KQTD20190929173906742); the Key Laboratory of Molecular Design for Plant Cell Factory of Guangdong Higher Education Institutes (2019KSYS006); the Shenzhen Government Peacock Plan (Y01226136); and the Thousand Young Talents Program. Research in the Groth lab is supported by the Lundbeck Foundation (R198-2015-269 and R313-2019-448), the European Research Council (ERC CoG 724436), and the Independent Research Fund Denmark (7016-00042B and 4092-00404B). Research at CPR is supported by the Novo Nordisk Foundation (NNF14CC0001).

AUTHOR CONTRIBUTIONS

C.M.H., D.J.P., H.H., and A.G. conceived the study and designed experiments. C.M.H. led the *in vivo* characterization of DNAJC9, including generating all cell lines and plasmids, pull-downs, Western blots, label-free mass spectrometry, and immunofluorescence. C.M.H. analyzed proteomics data in consultation with I.A.H. H.B. performed protein expression and purification, crystallization and structure determination, X-ray data collection and processing, structural analysis, and *in vitro* biochemistry. I.A.H. acquired mass spectrometry data in consultation with M.L.N. M.C. performed DNAJC9 SILAC pull-downs. A.G.-N. performed MCM2 and TONSL SILAC pull-downs. Y.L. performed plasmid supercoiling assays with H.B. and together with L.C. helped with protein purification and crystallization. N.R.-G. performed ChIP-seq experiments and data analysis. C.S. acquired mass spectrometry data in consultation with J.R. (Figures 1B and 1C). C.M.H., H.H., and A.G. wrote the manuscript with comments from all authors.

DECLARATION OF INTERESTS

C.M.H., D.J.P., H.H., and A.G. are inventors on a filed patent application covering the therapeutic targeting of TONSL for cancer therapy. A.G. is a co-founder and chief scientific officer (CSO) of Ankrin Therapeutics. A.G. is a member of *Molecular Cell*'s scientific advisory board.

Received: November 30, 2020
Revised: March 17, 2021
Accepted: March 25, 2021
Published: April 14, 2021

SUPPORTING CITATIONS

The following references appear in the Supplemental information: Heberle et al. (2015), Huang et al. (2009a), Huang et al. (2009b), Tachiwana et al. (2011).

REFERENCES

Adams, P.D., Afonine, P.V., Bunkóczi, G., Chen, V.B., Davis, I.W., Echols, N., Headd, J.J., Hung, L.-W., Kapral, G.J., Grosse-Kunstleve, R.W., et al. (2010). PHENIX: a comprehensive Python-based system for macromolecular structure solution. Acta Crystallogr. D Biol. Crystallogr. *66*, 213–221.

Afgan, E., Baker, D., van den Beek, M., Blankenberg, D., Bouvier, D., Čech, M., Chilton, J., Clements, D., Coraor, N., Eberhard, C., et al. (2016). The Galaxy platform for accessible, reproducible and collaborative biomedical analyses: 2016 update. Nucleic Acids Res. *44* (W1), W3–W10.

Alabert, C., Barth, T.K., Reverón-Gómez, N., Sidoli, S., Schmidt, A., Jensen, O.N., Imhof, A., and Groth, A. (2015). Two distinct modes for propagation of histone PTMs across the cell cycle. Genes Dev. *29*, 585–590.

Alekseev, O.M., Widgren, E.E., Richardson, R.T., and O'Rand, M.G. (2005). Association of NASP with HSP90 in mouse spermatogenic cells: stimulation of ATPase activity and transport of linker histones into nuclei. J. Biol. Chem. *280*, 2904–2911.

Allis, C.D., and Jenuwein, T. (2016). The molecular hallmarks of epigenetic control. Nat. Rev. Genet. *17*, 487–500.

Alvarez, F., Muñoz, F., Schilcher, P., Imhof, A., Almouzni, G., and Loyola, A. (2011). Sequential establishment of marks on soluble histones H3 and H4. J. Biol. Chem. *286*, 17714–17721.

Andrews, A.J., Chen, X., Zevin, A., Stargell, L.A., and Luger, K. (2010). The histone chaperone Nap1 promotes nucleosome assembly by eliminating nonnucleosomal histone DNA interactions. Mol. Cell *37*, 834–842.

Behan, F.M., Iorio, F., Picco, G., Gonçalves, E., Beaver, C.M., Migliardi, G., Santos, R., Rao, Y., Sassi, F., Pinnelli, M., et al. (2019). Prioritization of cancer therapeutic targets using CRISPR-Cas9 screens. Nature *568*, 511–516.

Bowman, A., Koide, A., Goodman, J.S., Colling, M.E., Zinne, D., Koide, S., and Ladurner, A.G. (2017). sNASP and ASF1A function through both competitive and compatible modes of histone binding. Nucleic Acids Res. *45*, 643–656.

Campos, E.I., Fillingham, J., Li, G., Zheng, H., Voigt, P., Kuo, W.-H.W., Seepany, H., Gao, Z., Day, L.A., Greenblatt, J.F., and Reinberg, D. (2010). The program for processing newly synthesized histones H3.1 and H4. Nat. Struct. Mol. Biol. *17*, 1343–1351.

Campos, E.I., Smits, A.H., Kang, Y.H., Landry, S., Escobar, T.M., Nayak, S., Ueberheide, B.M., Durocher, D., Vermeulen, M., Hurwitz, J., and Reinberg, D. (2015). Analysis of the histone H3.1 interactome: a suitable chaperone for the right event. Mol. Cell *60*, 697–709.

Chen, S., Rufiange, A., Huang, H., Rajashankar, K.R., Nourani, A., and Patel, D.J. (2015). Structure-function studies of histone H3/H4 tetramer maintenance during transcription by chaperone Spt2. Genes Dev. *29*, 1326–1340.

Clerico, E.M., Meng, W., Pozhidaeva, A., Bhasne, K., Petridis, C., and Gierasch, L.M. (2019). Hsp70 molecular chaperones: multifunctional allosteric holding and unfolding machines. Biochem. J. *476*, 1653–1677.

Cook, A.J., Gurard-Levin, Z.A., Vassias, I., and Almouzni, G. (2011). A specific function for the histone chaperone NASP to fine-tune a reservoir of soluble H3-H4 in the histone supply chain. Mol. Cell *44*, 918–927.

Corpet, A., De Koning, L., Toedling, J., Savignoni, A., Berger, F., Lemaître, C., O'Sullivan, R.J., Karlseder, J., Barillot, E., Asselain, B., et al. (2011). Asf1b, the necessary Asf1 isoform for proliferation, is predictive of outcome in breast cancer. EMBO J. *30*, 480–493.

Cox, J., and Mann, M. (2008). MaxQuant enables high peptide identification rates, individualized p.p.b.-range mass accuracies and proteome-wide protein quantification. Nat. Biotechnol. *26*, 1367–1372.

Cox, J., Neuhauser, N., Michalski, A., Scheltema, R.A., Olsen, J.V., and Mann, M. (2011). Andromeda: a peptide search engine integrated into the MaxQuant environment. J. Proteome Res. *10*, 1794–1805.

Daganzo, S.M., Erzberger, J.P., Lam, W.M., Skordalakes, E., Zhang, R., Franco, A.A., Brill, S.J., Adams, P.D., Berger, J.M., and Kaufman, P.D. (2003). Structure and function of the conserved core of histone deposition protein Asf1. Curr. Biol. *13*, 2148–2158.

DeNizio, J.E., Elsässer, S.J., and Black, B.E. (2014). DAXX co-folds with H3.3/H4 using high local stability conferred by the H3.3 variant recognition residues. Nucleic Acids Res. *42*, 4318–4331.

Doncheva, N.T., Morris, J.H., Gorodkin, J., and Jensen, L.J. (2019). Cytoscape StringApp: network analysis and visualization of proteomics data. J. Proteome Res. *18*, 623–632.

Dwane, L., Behan, F.M., Gonçalves, E., Lightfoot, H., Yang, W., van der Meer, D., Shepherd, R., Pignatelli, M., Iorio, F., and Garnett, M.J. (2021). Project Score database: a resource for investigating cancer cell dependencies and prioritizing therapeutic targets. Nucleic Acids Res. *49* (D1), D1365–D1372.

Ellis, J. (1987). Proteins as molecular chaperones. Nature *328*, 378–379.

Elsässer, S.J., and D'Arcy, S. (2013). Towards a mechanism for histone chaperones. Biochim. Biophys. Acta *1819*, 211–221.

Elsässer, S.J., Huang, H., Lewis, P.W., Chin, J.W., Allis, C.D., and Patel, D.J. (2012). DAXX envelops a histone H3.3-H4 dimer for H3.3-specific recognition. Nature *491*, 560–565.

Emsley, P., Lohkamp, B., Scott, W.G., and Cowtan, K. (2010). Features and development of Coot. Acta Crystallogr. D Biol. Crystallogr. *66*, 486–501.

ENCODE Project Consortium (2012). An integrated encyclopedia of DNA elements in the human genome. Nature *489*, 57–74.

English, C.M., Adkins, M.W., Carson, J.J., Churchill, M.E., and Tyler, J.K. (2006). Structural basis for the histone chaperone activity of Asf1. Cell *127*, 495–508.

Foltman, M., Evrin, C., De Piccoli, G., Jones, R.C., Edmondson, R.D., Katou, Y., Nakato, R., Shirahige, K., and Labib, K. (2013). Eukaryotic replisome components cooperate to process histones during chromosome replication. Cell Rep. *3*, 892–904.

Fujita, M., Kiyono, T., Hayashi, Y., and Ishibashi, M. (1997). In vivo interaction of human MCM heterohexameric complexes with chromatin. Possible involvement of ATP. J. Biol. Chem. *272*, 10928–10935.

CellPress
OPEN ACCESS

Fyodorov, D.V., and Kadonaga, J.T. (2003). Chromatin assembly in vitro with purified recombinant ACF and NAP-1. In Methods in Enzymology (Elsevier), pp. 499–515.

Gan, H., Serra-Cardona, A., Hua, X., Zhou, H., Labib, K., Yu, C., and Zhang, Z. (2018). The Mcm2-Ctf4-Polα axis facilitates parental histone H3-H4 transfer to lagging strands. Mol. Cell *72*, 140–151.e3.

Genest, O., Wickner, S., and Doyle, S.M. (2019). Hsp90 and Hsp70 chaperones: collaborators in protein remodeling. J. Biol. Chem. *294*, 2109–2120.

Groth, A., Ray-Gallet, D., Quivy, J.P., Lukas, J., Bartek, J., and Almouzni, G. (2005). Human Asf1 regulates the flow of S phase histones during replicational stress. Mol. Cell *17*, 301–311.

Groth, A., Corpet, A., Cook, A.J., Roche, D., Bartek, J., Lukas, J., and Almouzni, G. (2007). Regulation of replication fork progression through histone supply and demand. Science *318*, 1928–1931.

Gurard-Levin, Z.A., Quivy, J.P., and Almouzni, G. (2014). Histone chaperones: assisting histone traffic and nucleosome dynamics. Annu. Rev. Biochem. *83*, 487–517.

Hammond, C.M., Sundaramoorthy, R., Larance, M., Lamond, A., Stevens, M.A., El-Mkami, H., Norman, D.G., and Owen-Hughes, T. (2016). The histone chaperone Vps75 forms multiple oligomeric assemblies capable of mediating exchange between histone H3-H4 tetramers and Asf1-H3-H4 complexes. Nucleic Acids Res. *44*, 6157–6172.

Hammond, C.M., Strømme, C.B., Huang, H., Patel, D.J., and Groth, A. (2017). Histone chaperone networks shaping chromatin function. Nat. Rev. Mol. Cell Biol. *18*, 141–158.

Han, C., Chen, T., Li, N., Yang, M., Wan, T., and Cao, X. (2007). HDJC9, a novel human type C DnaJ/HSP40 member interacts with and cochaperones HSP70 through the J domain. Biochem. Biophys. Res. Commun. *353*, 280–285.

Hart, T., Chandrashekhar, M., Aregger, M., Steinhart, Z., Brown, K.R., MacLeod, G., Mis, M., Zimmermann, M., Fradet-Turcotte, A., Sun, S., et al. (2015). High-resolution CRISPR screens reveal fitness genes and genotype-specific cancer liabilities. Cell *163*, 1515–1526.

Heberle, H., Meirelles, G.V., da Silva, F.R., Telles, G.P., and Minghim, R. (2015). InteractiVenn: a web-based tool for the analysis of sets through Venn diagrams. BMC Bioinformatics *16*, 169.

Hu, H., Liu, Y., Wang, M., Fang, J., Huang, H., Yang, N., Li, Y., Wang, J., Yao, X., Shi, Y., et al. (2011). Structure of a CENP-A-histone H4 heterodimer in complex with chaperone HJURP. Genes Dev. *25*, 901–906.

Huang, D.W., Sherman, B.T., and Lempicki, R.A. (2009a). Bioinformatics enrichment tools: paths toward the comprehensive functional analysis of large gene lists. Nucleic Acids Res. *37*, 1–13.

Huang, D.W., Sherman, B.T., and Lempicki, R.A. (2009b). Systematic and integrative analysis of large gene lists using DAVID bioinformatics resources. Nat. Protoc. *4*, 44–57.

Huang, H., Strømme, C.B., Saredi, G., Hödl, M., Strandsby, A., González-Aguilera, C., Chen, S., Groth, A., and Patel, D.J. (2015). A unique binding mode enables MCM2 to chaperone histones H3-H4 at replication forks. Nat. Struct. Mol. Biol. *22*, 618–626.

Jarnuczak, A.F., Albornoz, M.G., Eyers, C.E., Grant, C.M., and Hubbard, S.J. (2018). A quantitative and temporal map of proteostasis during heat shock in Saccharomyces cerevisiae. Mol. Omics *14*, 37–52.

Jasencakova, Z., Scharf, A.N., Ask, K., Corpet, A., Imhof, A., Almouzni, G., and Groth, A. (2010). Replication stress interferes with histone recycling and predeposition marking of new histones. Mol. Cell *37*, 736–743.

Kaaij, L.J.T., Mohn, F., van der Weide, R.H., de Wit, E., and Bühler, M. (2019). The ChAHP complex counteracts chromatin looping at CTCF sites that emerged from SINE expansions in mouse. Cell *178*, 1437–1451.e14.

Lai, W.K.M., and Pugh, B.F. (2017). Understanding nucleosome dynamics and their links to gene expression and DNA replication. Nat. Rev. Mol. Cell Biol. *18*, 548–562.

Lambert, J.-P.P., Tucholska, M., Go, C., Knight, J.D., and Gingras, A.-C.C. (2015). Proximity biotinylation and affinity purification are complementary approaches for the interactome mapping of chromatin-associated protein complexes. J. Proteomics *118*, 81–94.

Langmead, B., and Salzberg, S.L. (2012). Fast gapped-read alignment with Bowtie 2. Nat. Methods *9*, 357–359.

Laskey, R.A., Honda, B.M., Mills, A.D., and Finch, J.T. (1978). Nucleosomes are assembled by an acidic protein which binds histones and transfers them to DNA. Nature *275*, 416–420.

Legeay, M., Doncheva, N.T., Morris, J.H., and Jensen, L.J. (2020). Visualize omics data on networks with Omics Visualizer, a Cytoscape app. F1000Res. *9*, 157.

Lewis, P.W., Elsaesser, S.J., Noh, K.M., Stadler, S.C., and Allis, C.D. (2010). Daxx is an H3.3-specific histone chaperone and cooperates with ATRX in replication-independent chromatin assembly at telomeres. Proc. Natl. Acad. Sci. U S A *107*, 14075–14080.

Luger, K., Mäder, A.W., Richmond, R.K., Sargent, D.F., and Richmond, T.J. (1997). Crystal structure of the nucleosome core particle at 2.8 A resolution. Nature *389*, 251–260.

Malay, A.D., Umehara, T., Matsubara-Malay, K., Padmanabhan, B., and Yokoyama, S. (2008). Crystal structures of fission yeast histone chaperone Asf1 complexed with the Hip1 B-domain or the Cac2 C terminus. J. Biol. Chem. *283*, 14022–14031.

Malecki, M.J., Sanchez-Irizarry, C., Mitchell, J.L., Histen, G., Xu, M.L., Aster, J.C., and Blacklow, S.C. (2006). Leukemia-associated mutations within the NOTCH1 heterodimerization domain fall into at least two distinct mechanistic classes. Mol. Cell. Biol. *26*, 4642–4651.

Marzluff, W.F., and Koreski, K.P. (2017). Birth and death of histone mRNAs. Trends Genet. *33*, 745–759.

Mattiroli, F., D'Arcy, S., and Luger, K. (2015). The right place at the right time: chaperoning core histone variants. EMBO Rep. *16*, 1454–1466.

Mayer, M.P., and Gierasch, L.M. (2019). Recent advances in the structural and mechanistic aspects of Hsp70 molecular chaperones. J. Biol. Chem. *294*, 2085–2097.

McCoy, A.J., Grosse-Kunstleve, R.W., Adams, P.D., Winn, M.D., Storoni, L.C., and Read, R.J. (2007). Phaser crystallographic software. J. Appl. Cryst. *40*, 658–674.

Mello, J.A., Sillje, H.H., Roche, D.M., Kirschner, D.B., Nigg, E.A., and Almouzni, G. (2002). Human Asf1 and CAF-1 interact and synergize in a repair-coupled nucleosome assembly pathway. EMBO Rep. *3*, 329–334.

Mendiratta, S., Gatto, A., and Almouzni, G. (2019). Histone supply: multitiered regulation ensures chromatin dynamics throughout the cell cycle. J. Cell Biol. *218*, 39–54.

Minor, W., Cymborowski, M., Otwinowski, Z., and Chruszcz, M. (2006). HKL-3000: the integration of data reduction and structure solution–from diffraction images to an initial model in minutes. Acta Crystallogr. D Biol. Crystallogr. *62*, 859–866.

Natsume, R., Eitoku, M., Akai, Y., Sano, N., Horikoshi, M., and Senda, T. (2007). Structure and function of the histone chaperone CIA/ASF1 complexed with histones H3 and H4. Nature *446*, 338–341.

Nicetto, D., and Zaret, K.S. (2019). Role of H3K9me3 heterochromatin in cell identity establishment and maintenance. Curr. Opin. Genet. Dev. *55*, 1–10.

O'Connell, B.C., Adamson, B., Lydeard, J.R., Sowa, M.E., Ciccia, A., Bredemeyer, A.L., Schlabach, M., Gygi, S.P., Elledge, S.J., and Harper, J.W. (2010). A genome-wide camptothecin sensitivity screen identifies a mammalian MMS22L-NFKBIL2 complex required for genomic stability. Mol Cell *40*, 645–57.

Pardal, A.J., Fernandes-Duarte, F., and Bowman, A.J. (2019). The histone chaperoning pathway: from ribosome to nucleosome. Essays Biochem. *63*, 29–43.

Perez-Riverol, Y., Csordas, A., Bai, J., Bernal-Llinares, M., Hewapathirana, S., Kundu, D.J., Inuganti, A., Griss, J., Mayer, G., Eisenacher, M., et al. (2019). The PRIDE database and related tools and resources in 2019: improving support for quantification data. Nucleic Acids Res. *47* (D1), D442–D450.

Petryk, N., Dalby, M., Wenger, A., Stromme, C.B., Strandsby, A., Andersson, R., and Groth, A. (2018). MCM2 promotes symmetric inheritance of modified histones during DNA replication. Science *361*, 1389–1392.

Powers, M.V., Clarke, P.A., and Workman, P. (2008). Dual targeting of HSC70 and HSP72 inhibits HSP90 function and induces tumor-specific apoptosis. Cancer Cell *14*, 250–262.

Quivy, J.P., Roche, D., Kirschner, D., Tagami, H., Nakatani, Y., and Almouzni, G. (2004). A CAF-1 dependent pool of HP1 during heterochromatin duplication. EMBO J. *23*, 3516–3526.

Rappsilber, J., Mann, M., and Ishihama, Y. (2007). Protocol for micro-purification, enrichment, pre-fractionation and storage of peptides for proteomics using StageTips. Nat. Protoc. *2*, 1896–1906.

Reverón-Gómez, N., González-Aguilera, C., Stewart-Morgan, K.R., Petryk, N., Flury, V., Graziano, S., Johansen, J.V., Jakobsen, J.S., Alabert, C., and Groth, A. (2018). Accurate recycling of parental histones reproduces the histone modification landscape during DNA replication. Mol. Cell *72*, 239–249.e5.

Richet, N., Liu, D., Legrand, P., Velours, C., Corpet, A., Gaubert, A., Bakail, M., Moal-Raisin, G., Guerois, R., Compper, C., et al. (2015). Structural insight into how the human helicase subunit MCM2 may act as a histone chaperone together with ASF1 at the replication fork. Nucleic Acids Res. *43*, 1905–1917.

Rosenzweig, R., Nillegoda, N.B., Mayer, M.P., and Bukau, B. (2019). The Hsp70 chaperone network. Nat. Rev. Mol. Cell Biol. *20*, 665–680.

Saredi, G., Huang, H., Hammond, C.M., Alabert, C., Bekker-Jensen, S., Forne, I., Reverón-Gómez, N., Foster, B.M., Mlejnkova, L., Bartke, T., et al. (2016). H4K20me0 marks post-replicative chromatin and recruits the TONSL–MMS22L DNA repair complex. Nature *534*, 714–718.

Schulte, D., Richter, A., Burkhart, R., Musahl, C., and Knippers, R. (1996). Properties of the human nuclear protein p85Mcm. Expression, nuclear localization and interaction with other Mcm proteins. Eur. J. Biochem. *235*, 144–151.

Senapati, P., Sudarshan, D., Gadad, S.S., Shandilya, J., Swaminathan, V., and Kundu, T.K. (2015). Methods to study histone chaperone function in nucleosome assembly and chromatin transcription. Methods Mol. Biol. *1288*, 375–394.

Shannon, P., Markiel, A., Ozier, O., Baliga, N.S., Wang, J.T., Ramage, D., Amin, N., Schwikowski, B., and Ideker, T. (2003). Cytoscape: a software environment for integrated models of biomolecular interaction networks. Genome Res. *13*, 2498–2504.

Suh, W.C., Burkholder, W.F., Lu, C.Z., Zhao, X., Gottesman, M.E., and Gross, C.A. (1998). Interaction of the Hsp70 molecular chaperone, DnaK, with its co-chaperone DnaJ. Proc. Natl. Acad. Sci. U S A *95*, 15223–15228.

Tachiwana, H., Osakabe, A., Shiga, T., Miya, Y., Kimura, H., Kagawa, W., and Kurumizaka, H. (2011). Structures of human nucleosomes containing major histone H3 variants. Acta Crystallogr. D Biol. Crystallogr. *67*, 578–583.

Tagami, H., Ray-Gallet, D., Almouzni, G., and Nakatani, Y. (2004). Histone H3.1 and H3.3 complexes mediate nucleosome assembly pathways dependent or independent of DNA synthesis. Cell *116*, 51–61.

Tang, Y., Poustovoitov, M.V., Zhao, K., Garfinkel, M., Canutescu, A., Dunbrack, R., Adams, P.D., and Marmorstein, R. (2006). Structure of a human ASF1a-HIRA complex and insights into specificity of histone chaperone complex assembly. Nat. Struct. Mol. Biol. *13*, 921–929.

Tyanova, S., Temu, T., Sinitcyn, P., Carlson, A., Hein, M.Y., Geiger, T., Mann, M., and Cox, J. (2016). The Perseus computational platform for comprehensive analysis of (prote)omics data. Nat. Methods *13*, 731–740.

Tyler, J.K., Collins, K.A., Prasad-Sinha, J., Amiott, E., Bulger, M., Harte, P.J., Kobayashi, R., and Kadonaga, J.T. (2001). Interaction between the Drosophila CAF-1 and ASF1 chromatin assembly factors. Mol. Cell. Biol. *21*, 6574–6584.

Wall, D., Zylicz, M., and Georgopoulos, C. (1994). The NH2-terminal 108 amino acids of the Escherichia coli DnaJ protein stimulate the ATPase activity of DnaK and are sufficient for lambda replication. J. Biol. Chem. *269*, 5446–5451.

Wang, H., Wang, M., Yang, N., and Xu, R.-M.M. (2015). Structure of the quaternary complex of histone H3-H4 heterodimer with chaperone ASF1 and the replicative helicase subunit MCM2. Protein Cell *6*, 693–697.

Wang, Q.-S., Zhang, K.-H., Cui, Y., Wang, Z.-J., Pan, Q.-Y., Liu, K., Sun, B., Zhou, H., Li, M.-J., Xu, Q., et al. (2018). Upgrade of macromolecular crystallography beamline BL17U1 at SSRF. Nucl. Sci. Tech. *29*, 68.

Winter, G. (2010). xia2: an expert system for macromolecular crystallography data reduction. J. Appl. Cryst. *43*, 186–190.

Yadav, T., Quivy, J.P., and Almouzni, G. (2018). Chromatin plasticity: a versatile landscape that underlies cell fate and identity. Science *361*, 1332–1336.

Zhang, R., Poustovoitov, M.V., Ye, X., Santos, H.A., Chen, W., Daganzo, S.M., Erzberger, J.P., Serebriiskii, I.G., Canutescu, A.A., Dunbrack, R.L., et al. (2005). Formation of MacroH2A-containing senescence-associated heterochromatin foci and senescence driven by ASF1a and HIRA. Dev. Cell *8*, 19–30.

Zhang, Y., Liu, T., Meyer, C.A., Eeckhoute, J., Johnson, D.S., Bernstein, B.E., Nusbaum, C., Myers, R.M., Brown, M., Li, W., and Liu, X.S. (2008). Model-based analysis of ChIP-Seq (MACS). Genome Biol. *9*, R137.

Zhang, W., Tyl, M., Ward, R., Sobott, F., Maman, J., Murthy, A.S., Watson, A.A., Fedorov, O., Bowman, A., Owen-Hughes, T., et al. (2013). Structural plasticity of histones H3-H4 facilitates their allosteric exchange between RbAp48 and ASF1. Nat. Struct. Mol. Biol. *20*, 29–35.

Zhang, Z., Jing, J., Ye, Y., Chen, Z., Jing, Y., Li, S., Hong, W., Ruan, H., Liu, Y., Hu, Q., et al. (2020). Characterization of the dual functional effects of heat shock proteins (HSPs) in cancer hallmarks to aid development of HSP inhibitors. Genome Med. *12*, 101.

CellPress
OPEN ACCESS

STAR★METHODS

KEY RESOURCES TABLE

REAGENT or RESOURCE	SOURCE	IDENTIFIER
**Antibodies**		
TONSL	Sigma	Cat no. HPA046494; RRID: AB_2679673
CAF1A/p150	Quivy et al., 2004	N/A
MCM2	Bethyl Laboratories	Cat no. A300-122A; RRID: AB_155897
CAF1B/p60	Quivy et al., 2004	N/A
DNAJC9	AbCam	Cat no. ab150394; RRID: AB_2890229
HA	Cell Signaling Technology	Cat no. C29F4 #3724; RRID: AB_1549585
DAXX	Sigma	Cat no. HPA008736; RRID: AB_1078625
HJURP	Sigma	Cat no. HPA008436; RRID: AB_1850757
Flag	Sigma	Cat no. F7425; RRID: AB_439687
H3	Abcam	Cat no. ab1791; RRID: AB_302613
H4	Millipore	Cat no. 05-858; RRID: AB_390138
Tubulin	Abcam	Cat no. ab6160; RRID: AB_305328
BAG2	Abcam	Cat no. ab79406; RRID: AB_1603761
HSC/HSP70	Santa Cruz Biotechnology	Cat no. sc-24; RRID: AB_627760
HSC70	Santa Cruz Biotechnology	Cat no. sc-7298; RRID: AB_627761
Flag	Sigma	Cat no. F3165; RRID: AB_259529
Goat Anti-Mouse-Alexa488	Thermo Scientific	Cat no. A-11029; RRID: AB_138404
**Bacterial and virus strains**		
E.coli: BL21 (DE3)-RIL	Stratagene	Cat no. 230245
**Chemicals, peptides, and recombinant proteins**		
Doxycycline	Clontech	Cat no. 631311
ULP1 protease	Homemade	N/A
TEV protease	Homemade	N/A
3C protease	Homemade	N/A
Drosophila topoisomerase I (dTopo I)	Homemade	N/A
Micrococcal Nuclease Solution	Thermo Scientific	Cat no. 88216
Benzonase Nuclease	Millipore	Cat no. 70746
RNase A	Sigma	Cat no. R4875
Sequencing Grade Modified Trypsin	Promega	Cat no. V5113
Trypsin, Proteomics Grade	Sigma	Cat no. T6567
**Critical commercial assays**		
MinElute PCR Purification Kit	QIAGEN	Cat no. 28004
MinElute Reaction Cleanup Kit	QIAGEN	Cat no. 28204
truChIP Chromatin Shearing Kit	Covaris	Cat no. 520127
KAPA Hyperprep Kit	Kappa Biosystems, Roche	Cat no. KK8504
**Deposited data**		
Structure of the DNAJC9 αB–H3.3–H4–MCM2 HBD quaternary complex	This study	PDB: 7CIZ
Structure of the DNAJC9 HBΔ–H3.3–H4–MCM2 HBD quaternary complex	This study	PDB: 7CJ0
ChIP-seq Data	This study	GEO: GSE154445
Raw Mass Spectrometry Data Sets	This study	PRIDE: PXD020268

(*Continued on next page*)

Continued		
REAGENT or RESOURCE	SOURCE	IDENTIFIER
Raw Data for Western Blots, Gel-based Assays and ITC	This study	Mendeley Data: https://doi.org/10.17632/y9mgyw9r59.1
Experimental models: Cell lines		
U-2-OS FRT/TO Flp-In T-REX	Malecki et al., 2006	N/A
U-2-OS FRT/TO Flp-In T-REX FlagHA-MCM2-WT	Huang et al., 2015	N/A
U-2-OS FRT/TO Flp-In T-REX FlagHA-MCM2-Y81A-Y90A	Huang et al., 2015	N/A
U-2-OS FRT/TO Flp-In T-REX U-2-OS eGFP-TONSL-WT	Saredi et al., 2016	N/A
U-2-OS FRT/TO Flp-In T-REX U-2-OS eGFP-TONSL-N571A	Saredi et al., 2016	N/A
HeLa S3	ATCC	Cat no. CCL-2.2; RRID: CVCL_0058
HeLa S3 pTetOne Puro H3.1-FlagHA	This study	N/A
HeLa S3 pTetOne Puro H3.2-FlagHA	This study	N/A
HeLa S3 pTetOne Puro H3.3-FlagHA	This study	N/A
HeLa S3 pTetOne Puro H4-FlagHA	This study	N/A
HeLa S3 pTetOne Puro CENPA-FlagHA	This study	N/A
HeLa S3 pTetOne Puro H3.1T-FlagHA	This study	N/A
HeLa S3 pTetOne Puro H3.1-E105A-D106A-FlagHA	This study	N/A
U-2-OS FRT/TO Flp-In T-REX DNAJC9-MYC-Flag (WT)	This study	N/A
U-2-OS FRT/TO Flp-In T-REX DNAJC9-H43Q-D45N-MYC-Flag (J)	This study	N/A
U-2-OS FRT/TO Flp-In T-REX DNAJC9-Q224A-R227A-M238A-Y242A-MYC-Flag (4A)	This study	N/A
U-2-OS FRT/TO Flp-In T-REX DNAJC9-H43Q-D45N-Q224A-R227A-M238A-Y242A-MYC-Flag (4AJ)	This study	N/A
293FT	Thermo Scientific	Cat no. R70007; RRID: CVCL_6911
Drosophila S2-DRSC cells	Drosophila Genomics Resource Center; Stock No. 181	RRID: CVCL_Z992
Oligonucleotides		
NGS indexed PentAdapters	PentaBase	Cat no. SKU 310
Silencer® Select siDNAJC9	Thermo Scientific	Cat no. s23354
Silencer® Select siBAG2	Thermo Scientific	Cat no. s18295
Silencer® Select siHSPA8 (siHSP7C)	Thermo Scientific	Cat no. s6987
Silencer® Select Negative Control No. 1	Thermo Scientific	Cat no. 4390843
Recombinant DNA		
ΦX174 RF I DNA	NEB	Cat no. N3021S
pTetOne Puro H3.1-FlagHA	This study	N/A
pTetOne Puro H3.2-FlagHA	This study	N/A
pTetOne Puro H3.3-FlagHA	This study	N/A
pTetOne Puro H4-FlagHA	This study	N/A
pTetOne Puro CENPA-FlagHA	This study	N/A
pTetOne Puro H3.1T-FlagHA	This study	N/A
pTetOne Puro H3.1-E105A-D106A-FlagHA	This study	N/A

(*Continued on next page*)

Continued

REAGENT or RESOURCE	SOURCE	IDENTIFIER
pCMV6-DNAJC9-siRNA-resistent-Myc-Flag (WT)	This study	N/A
pCMV6-DNAJC9-siRNA-resistent-L216A-I220A-Myc-Flag	This study	N/A
pCMV6-DNAJC9-siRNA-resistent-Q224A-R227A-Myc-Flag	This study	N/A
pCMV6-DNAJC9-siRNA-resistent-M238A-Y242A-MycFlag	This study	N/A
pCMV6-DNAJC9-siRNA-resistent-E195A-E199A- MycFlag	This study	N/A
pcDNA5-DNAJC9-siRNA-resistent-Myc-Flag (WT)	This study	N/A
pcDNA5-DNAJC9-siRNA-resistent-Q224A-R227A-M238A-Y242A-Myc-Flag (4A)	This study	N/A
pcDNA5-DNAJC9-siRNA-resistent-H43Q-D45N-Myc-Flag (J)	This study	N/A
pcDNA5-DNAJC9-siRNA-resistent-H43Q-D45N-Q224A-R227A-M238A-Y242A-Myc-Flag (4AJ)	This study	N/A
Software and algorithms		
HKL-3000	Minor et al., 2006	https://www.hkl-xray.com/hkl-3000
Xia2	Winter, 2010	https://xia2.github.io/
Phenix	Adams et al., 2010	https://www.phenix-online.org/
PyMOL	The PyMOL Molecular Graphics System, Version 1.7, Schrodinger, LLC	https://www.pymol.org/2/
MaxQuant 1.6.3.4	Cox and Mann, 2008; Cox et al., 2011	https://maxquant.net/maxquant/
Perseus 1.6.2.3	Tyanova et al., 2016	https://maxquant.net/perseus/
GraphPad Prism v8.4	GraphPad Software	https://www.graphpad.com/scientific-software/prism/
R 3.6.2	The R Foundation	https://cran.r-project.org/
Cytoscape 3.8.0	Shannon et al., 2003	https://cytoscape.org/
stringApp 1.5.1	Doncheva et al., 2019	http://apps.cytoscape.org/
Omics Visualizer app 1.3.0	Legeay et al., 2020	http://apps.cytoscape.org/
Trim Galore!	Babraham Bioinformatics	https://www.bioinformatics.babraham.ac.uk/projects/trim_galore/
Bowtie2	Langmead and Salzberg, 2012	https://github.com/BenLangmead/bowtie2
SeqMonk	Babraham Bioinformatics	https://www.bioinformatics.babraham.ac.uk/projects/seqmonk/
Galaxy	Afgan et al., 2016	RRID: SCR_006281; https://galaxyproject.org/
MACS	Zhang et al., 2008	https://github.com/macs3-project/MACS
Other		
Glutathione Sepharose 4B beads	GE Healthcare	Cat no. 17-0756-04
IMAC Sepharose 6 Fast Flow	GE Healthcare	Cat no. 17-0921-09
HiTrap Heparin HP (5 mL)	GE Healthcare	Cat no. 17-0407-03
HiLoad 16/600 Superdex 200 pg	GE Healthcare	Cat no. 28-9893-35
GFP-Trap® Magnetic Agarose	Chromatek	Cat no. GTMA-20
ANTI-FLAG M2 Affinity Gel	Sigma	Cat no. A2220
Pierce Anti-HA Agarose	Thermo Scientific	Cat no. 26181
Pierce Anti-HA Magnetic Beads	Thermo Scientific	Cat no. 88836

(*Continued on next page*)

Continued		
REAGENT or RESOURCE	SOURCE	IDENTIFIER
Dynabeads Protein G for Immunoprecipitation	Thermo Scientific	Cat no. 10003D
Agencourt AMPure XP beads	Beckman Coulter	Cat no. A63881

RESOURCE AVAILABILITY

Lead contact

Further information and requests for resources and reagents should be directed to and will be fulfilled by the Lead Contact, Anja Groth (anja.groth@cpr.ku.dk).

Materials availability

All stable and unique reagents generated in this study are available from the Lead Contact subject to a Materials Transfer Agreement.

Data and code availability

Mass spectrometry datasets have been deposited to the ProteomeXchange Consortium via the PRIDE (Perez-Riverol et al., 2019) partner repository with the dataset identifier PXD020268 and processed data is presented in Table S1. Coordinates and structure factors have been deposited in the Protein Data Bank under accession codes: 7CIZ (the DNAJC9 αB–H3.3–H4–MCM2 HBD quaternary complex) and 7CJ0 (the DNAJC9 HBD–H3.3–H4–MCM2 HBD MCM2 quaternary complex). High-throughput sequencing data were submitted to NCBI Gene Expression Omnibus (GEO) under the accession identifier GSE154445. Raw data for western blots, gel-based assays and ITC have been deposited at Mendeley Data (https://doi.org/10.17632/y9mgyw9r59.2). Source data for other experiments are provided in Table S2. R scripts for data visualization are available upon request. Bubble plot R scripts were adapted from scripts written by Manuel Garcia Albornoz (Jarnuczak et al., 2018). All other data supporting the findings of this study are available from the corresponding authors on reasonable request.

EXPERIMENTAL MODEL AND SUBJECT DETAILS

Cell lines

Cell line generation and transfection

MCM2 (U-2-OS-FlpIn-Flag-HA-MCM2 WT and Y81A-Y90A) and TONSL (U-2-OS-FlpIn-eGFP-TONSL WT and N571A) cell lines were published previously (Huang et al., 2015; Saredi et al., 2016). Cell lines expressing DNAJC9 or histones from pLVX-TetOne-Puro constructs were created via lentiviral transduction of HeLa S3 suspension cells and Puromycin selection (2 μg/ml) 20-24 hours post-transduction. Lentivirus containing media supernatants were harvested and 0.45 μm syringe filtered 40-60 hours after transfection of 293FT cells with calcium phosphate precipitates of pLVX-TetOne-Puro, pCMV-VSV and pAX8 plasmids. Cell lines expressing DNAJC9 in the U-2-OS Flp-In T-REx system (Malecki et al., 2006) were created by co-transfection of pcDNA5-FRT-TO-DNAJC9 constructs and pOG44-Flp-recombinase using standard Lipofectamine 2000 transfection protocols (Thermo Scientific) and selection after 54-64 hours with Hygromycin B (200 μg/ml) and Blasticidin (5 μg/ml). All cell lines tested negative for Mycoplasma contamination, but H3.2-, H3.3- and CENPA-FlagHA cell lines were not tested separately but derived from a parental HeLa S3 cell line that tested negative. Transient transfections with pCMV6-DNAJC9 constructs were performed using Lipofectamine 2000 (Thermo Scientific). U-2-OS, HeLa S3 and 293FT cell lines originate from female subjects.

Cell culture

Unless stated otherwise cells were grown in DMEM with Glutamax (GIBCO), 10% FBS (Hyclone) and 1% penicillin/streptomycin (GIBCO) at 37°C with 5% CO_2. HeLa S3 pLVX-TetOne-Puro cell lines were grown under Puromycin selection (1 μg/ml, P8833, Sigma) and protein expression induced with Doxycycline (100 ng/ml, 36-48 hours). U-2-OS Flp-In T-REx cells were grown under Blasticidin (5 μg/ml) selection and additionally Hygromycin B (100-200 μg/ml) for cell lines with pcDNA5-FRT-TO integration, protein expression was induced with Tetracycline (1-2 μg/ml, 24-48 hours). For SILAC experiments cells were grown in RPMI 1640 Medium for SILAC supplemented with dialyzed FBS (Thermo Scientific), MEM non-essential amino acid mix (Thermo Scientific), Glutamax (Thermo Scientific), and isotopically labeled arginine (316 μM) and lysine (547 μM). Double SILAC experimental conditions employed heavy Lys8-Arg6 or light Lys0-Arg0 amino acid pairs and triple SILAC employed heavy Lys8-Arg10, medium Lys4-Arg6, or light Lys0-Arg0. Amino acid pairs were swapped between biological replicates of SILAC experiments, except the U-2-OS Flp-In T-REx control cell line for triple SILAC experiments which was cultured with light amino acids in both biological replicates. Isotope labeled amino acids sourced as follows: Arg0 and Lys0 (A6969 and L8662, Sigma); Arg6, Arg10, Lys4 and Lys8 (CNLM-2265-H1, CNLM-539-H1, DLM-2640-1 and CNLM-291-H-1, Cambridge Isotope Laboratories). *Drosophila* S2 cells (male) were grown in M3+BPYE media: Shields and Sang M3 Insect Medium (Sigma, S-8398), KHCO3 (Sigma, 12602), yeast extract (Sigma, Y-1000), bactopeptone (BD, 211705), 10% heat-inactivated FBS (Hyclone) and 1X penicillin/streptomycin (GIBCO) at 25°C with 5% CO2.

METHOD DETAILS

Plasmid construction

Coding sequences for histones H3.1 (HIST1H3A), H3.2 (HIST2H3C), H3.3 (H3F3A), H3.1T (HIST3H3) and CENPA, with silent point mutations inactivating internal BamHI (CENPA) and AgeI (H3.3) restriction sites and C-terminal Flag-HA tags (GGTG DYKDDDDKLDGGYPYDVPDYA), were synthesized and cloned (by Genscript) between 5′ EcoRI and 3′ BamHI sites of the pLVX-TetOne-Puro vector (631849, Clontech). DNAJC9 siRNA resistance cassettes designed against Silencer Select siRNAs (s23352, s23353, s23354, Thermo Scientific) were synthesized (by Genscript) and PCR amplified, to use as “mega-primer” pairs, to insert siRNA resistance cassettes into the pCMV6-DNAJC9-Myc-Flag vector (RC215630, Origene) by site-directed mutagenesis. Double alanine mutants in the DNAJC9 histone binding interface were incorporated during siRNA resistance cassette synthesis (L216A-I220A, Q224A-R227A and M238A-Y242A) or by site-directed mutagenesis (E195A-E199A). DNAJC9-Myc-Flag expressing constructs were sub-cloned into pLVX-TetOne-puro and pcDNA5-FRT-TO using native or PCR introduced 5′ EcoRI and 3′ BamHI restriction sites. DNAJC9 J domain (H43Q-D45N), 4A (Q224A-R227A-M238A-Y242A) and 4AJ mutants (H43Q-D45N-Q224A-R227A-M238A-Y242A) were generated by site-directed mutagenesis of the aforementioned pcDNA5-FRT-TO-DNAJC9-Myc-Flag constructs. H3.1 E105A-D106A was generated by site-directed mutagenesis of pLVX-TetOne-Puro-H3.1-Flag-HA. Site-directed mutagenesis was performed using established QuickChange mutagenesis protocols (Stratagene) or Infusion HD-directed mutagenesis (Takara). For Infusion HD-directed mutagenesis parental plasmids were amplified with Phusion HF (F530S, Thermo Scientific) using mutagenic primers that also create homologous arms which, after PCR purification (QIAgen) and Dpn1 digest (NEB), were recombined through Infusion HD cloning (Takara).

Cloning and protein preparation in bacteria

The human DNAJC9 gene was codon-optimized and synthesized (GENEWIZ). The full-length DNAJC9 (amino acids, aa 1–260) and truncated fragments including a.a 1–170, 171–249, 171–211, and 212–249 were cloned into the pGEX-6P-1 vector, respectively. The mutants of the histone binding domain (HBD; 171–249) of DNAJC9, including the double mutants L216A-I220A, Q224A-R227A, F234A-L235A and M238A-Y242A, and the multiple mutants E195A-E196A-E199A-A200E (4A1) and Q224A-R227A-M238A-Y242A (4A), were introduced by standard PCR procedure, respectively. The cDNA of full-length ASF1A, MCM2 HBD (43–160), NASP HBD (30–340), NASP HBD (1-340) and SPT2 HBD (571–685) were also cloned into the pGEX-6P-1 vector, respectively. For expression of these GST-tagged proteins, the corresponding plasmids were transformed into BL21 (DE3)-RIL cell strain (Stratagene), and cultured using Luria-Bertani (LB) medium supplemented with ampicillin (100 μg/L) and chloramphenicol (34 μg/L) at 37°C to an OD600 of ∼1.0-1.2. Then, protein expression was induced with 0.5 mM isopropyl β-D-1-thiogalactopyranoside (IPTG) and cells were further incubated overnight at 20°C. These expressed GST-tagged proteins were first purified with Glutathione Sepharose 4B beads (GE Healthcare), and were further purified by a HiLoad 16/600 Superdex 200 column (GE Healthcare) using a buffer of 20 mM Tris, pH 7.5, 500 mM NaCl. For some experiments, the GST-tags of GST-DNAJC9, DNAJC9 HBD, DNAJC9 HBD 4A, ASF1A and NASP HBD (1–340) were removed by 3C protease before the gel-filtration step.

The human histones H3.1–H4, H3.3–H4 and H3.3 (E105A-D106A)–H4 tetramers were co-expressed and purified in a similar way to our previous study (Huang et al., 2015) with small modifications. The previous purification step with the ceramic hydroxyapatite column was replaced with a HiLoad 16/600 Superdex 200 column purification using a buffer of 20 mM Tris, pH 7.5, 2 M NaCl. The H2A–H2B dimer was also co-expressed with a 6xHis-tag on H2A. The H2A–H2B dimer was first captured by Ni Sepharose 6 Fast Flow beads (GE Healthcare) in a buffer of 20 mM Tris, pH 7.5, 2 M NaCl, and eluted with a buffer of 20 mM Tris, pH 7.5, 1 M NaCl containing 500 mM imidazole, then the 6xHis-tags were removed with TEV protease and further purified by a HiLoad 16/600 Superdex 200 column with a buffer containing 2 M NaCl. The histones octamer was reconstituted by mixing H3.1–H4 tetramer and H2A–H2B dimer at a molar ratio of 1:2.4 in a buffer of 20 mM Tris, pH 7.5, 2 M NaCl, and further purified by a gel-filtration step. The complexes of MCM2 HBD (61–130)–H3.3–H4 and MCM2 HBD (61–130)–H3.3 (57–135)–H4 were purified as described previously (Huang et al., 2015).

For crystallization, the Cys243 was mutated to serine in DNAJC9 HBD (171–249) and DNAJC9 αB (204–249) constructs. GST-tagged DNAJC9 HBD C243S and DNAJC9 αB C243S were purified as above, but the GST-tags were removed by 3C protease before the final gel-filtration step. The purified DNAJC9 HBD C243S and DNAJC9 αB C243S were respectively mixed and incubated with the purified MCM2 HBD (61–130)–H3.3 (57–135)–H4 complex at a molar ratio of 1:1, and further purified on a HiLoad 16/600 Superdex 200 column using buffers of 20 mM Tris, pH 7.5, 1 M NaCl and 20 mM Tris, pH 7.5, 500 mM NaCl, respectively. The resulting quaternary complexes of DNAJC9 HBD–H3.3–H4–MCM2 HBD and DNAJC9 αB–H3.3–H4–MCM2 HBD were concentrated and stored at −80°C for further usage.

Crystallization, X-ray data collection and structure determination

All our crystals in this study were obtained with the sitting-drop vapor-diffusion method at 20°C. The DNAJC9 αB–H3.3–H4–MCM2 HBD quaternary complex at a concentration of 20.8 mg/ml was crystallized in 0.05 M lithium sulfate, 0.05 M sodium sulfate; 0.05 M Tris, pH 8.5, 30% (v/v) PEG400. The DNAJC9 HBD–H3.3–H4–MCM2 HBD MCM2 quaternary complex at a concentration of 20 mg/ml was crystallized in 0.17 M sodium acetate, 0.1 M Tris, pH 8.5, 25% (v/v) PEG 4000, 20% (v/v) Glycerol.

As the mother liquors contained high concentrations of glycerol and PEG400, respectively, which could serve as cryoprotectants, all crystals were directly flash-frozen in liquid nitrogen. Diffraction data for the crystal of the DNAJC9 αB–H3.3–H4–MCM2 HBD com-

plex was collected at a wavelength of 0.9774 Å at beamline 18U (BL18U1), at Shanghai Synchrotron Radiation Facility (SSRF), China. X-ray dataset was processed with the program HKL3000 (Minor et al., 2006). Diffraction data for the crystal of the DNAJC9 HBD–H3.3–H4–MCM2 HBD complex was collected at a wavelength of 0.9792 Å at beamline 17U (BL17U1) at SSRF (Wang et al., 2018). X-ray dataset was processed in the Xia2 pipeline (Winter, 2010).

Both of the structures were determined by molecular replacement in PHASER (McCoy et al., 2007) with our previous structure of MCM2 HBD–H3.3–H4 (PDB 5BNV) (Huang et al., 2015) as the search model, and were manually modified using Coot (Emsley et al., 2010) and refined in PHENIX (Adams et al., 2010). The final structures 7CIZ (the DNAJC9 αB–H3.3–H4–MCM2 HBD complex; Ramachandran favored 98.5% and allowed 1.5%), and 7CJ0 (the DNAJC9 HBD–H3.3–H4–MCM2 HBD complex; Ramachandran favored 98.1% and allowed 1.9%), were refined to 1.80 and 2.50 Å, respectively. The dataset of 7CJ0 was partially twined (twin fraction about 0.13), and a merohedral twin law (-h, -k, l) was applied only for the final round of refinement in PHENIX. Data collection and refinement statistics are listed in Table 1. All the structural figures in this study were prepared with PyMOL (The PyMOL Molecular Graphics System, Schrödinger).

GST pulldowns

For truncation mapping, 50 μl of Glutathione Sepharose 4B beads was suspended with 200 μl of binding buffer (20 mM Tris, pH 7.5, 500 mM NaCl), and 1.5 nmol of GST-tagged DNAJC9 and truncation fragments were added and incubated at 4°C for 30 min. Then, 1.5 nmol of MCM2 HBD–H3.3–H4 complex or H3.3–H4 tetramer were added and incubated at 4°C for another 2 hours. The beads were washed four times with 1 mL of washing buffer (20 mM Tris, pH 7.5, 500 mM NaCl, 0.5% (v/v) Triton X-100) before adding 50 μl of sample loading buffer. GST pulldowns of GST-tagged DNAJC9 HBD and its mutants with MCM2 HBD–H3.3–H4 complex, and GST pulldowns of GST-tagged DNAJC9 HBD, full-length ASF1A, MCM2 HBD (43–160), NASP HBD (30–340) and SPT2 HBD (571–685) with H3.3–H4 and H3.3 (E105A-D106A)–H4 tetramers, were performed in the same manner. All samples were analyzed by 15% reducing SDS-PAGE.

Isothermal titration calorimetry (ITC)

All the ITC titrations were performed on a MicroCal PEAQ-ITC (Malvern Panalytical Ltd) at 20°C. Protein samples were buffer-exchanged to 20 mM Tris-HCl, pH 7.5, 500 mM NaCl. A 50 μl of DNAJC9 HBD or the 4A mutant at a concentration of 300 μM was loaded into the syringe, while a 250 μl of H3.3–H4 or H3.1–H4 at a concentration of 30 μM (dimers) was loaded into the cell. The titration protocol consisted of 18 successive injections of 2 μl, with a spacing time of 250 s between each injection. The datasets were processed with the Origin software package (OriginLab) and the curves were fit using the ‘one set of sites’ model. The average equilibrium dissociation constant, K_d, was determined from 3 independent experiments.

Plasmid supercoiling assay

DNA supercoiling assays were performed as described (Fyodorov and Kadonaga, 2003; Huang et al., 2015; Senapati et al., 2015), with the following modifications. The cDNA of ND423 fragment of *Drosophila* topoisomerase I (dTopo I) was synthesized by Sangon Biotech (Shanghai), and then cloned into a modified pRSFDuet-1 vector, with an N-terminal 6xHis-SUMO tag. The resulting ND423 plasmid was transformed into BL21 (DE3)-RIL cell strain for expression, and the expressed protein was purified by Ni Sepharose 6 Fast Flow beads. The elution after Ni-affinity purification was dialysed against 2 l of buffer containing 20 mM HEPES, pH 7.5, 0.2 mM EDTA, 200 mM NaCl, 0.02% (v/v) NP-40, 1 mM DTT, 0.2 mM PMSF at 4°C for 4 hours. Then, the protein sample was concentrated to 1.0 mg/ml, added 50% (v/v) glycerol, and stored at −20°C for further usage.

The ΦX174 RF I DNA (purchased from NEB) was relaxed with dTopo I (ND423; 25 ng enzyme per 100 ng DNA) in assembly buffer (10 mM Tris, pH 7.5, 125 mM NaCl, 2 mM MgCl2, 0.5 mM DTT and 0.1 mg/ml BSA) and incubated at 37°C for 60 min. At the same time, 160 ng histone octamer, were mixed with 174 ng ASF1A (2.5-fold to H3–H4 dimer), 280 ng NASP HBD (1–340; 2.5-fold to H3–H4 dimer), 230 ng DNAJC9 (2.5-fold to H3–H4 dimer), and 460 ng DNAJC9 (5-fold to H3–H4 dimer), respectively, in assembly buffer with a final volume of 20 μl. The mixtures were incubated at 37°C for 30 min. To initiate the assembly reaction, 5 μl of the relaxation mixture (containing 100 ng of relaxed DNA) was mixed with the 20 μl of chaperone-histone mixture and incubated for 120 min at 37°C. After that, 25 μl of stop buffer (20 mM Tris, pH 8.0, 20 mM EDTA, 1% SDS and 0.5 mg/ml proteinase K) was added to remove the proteins and incubated at 50°C for 20 min. Phenol/chloroform DNA extraction and ethanol precipitation were then performed. DNA samples were analyzed on a 1% agarose gel with 1 × TBE buffer (89 mM Tris borate, 2 mM EDTA) at 100 V for 4 hours. The gel was visualized by ethidium bromide (EB) staining.

Cell extracts

Soluble extracts were generally prepared by extracting cell pellets washed twice in PBS with ice-cold chromatin wash buffers (ChWB), and supernatants clarified by centrifugation (16,000 g, 5 mins) and filtration (0.45 μm). ChWB: NaCl (300 mM), Nonidet P40 (0.5%), HEPES.NaOH or Tris.HCl (50 mM, pH 7.9/7.6), EDTA (0.2 mM), glycerol (5%), NaF (5 mM) and β-Glycerolphosphate (10 mM), Phenylmethanesulfonyl fluoride (0.1 mM), Leupeptin (10 μg/ml), Pepstatin A (10 μg/ml), Trichostatin A (100 ng/ml), Na_3VO_4 (0.2 mM). For siRNA depleted histone pulldowns cells were extracted 96 hours after Silencer® Select siRNA depletion (Thermo Scientific: siDNAJC9, s23354; siBAG2, s18295; siHSPA8 (siHSP7C), s6987; Negative Control No. 1) with 48 hours of H3.1 or H4-FlagHA expression (100 ng/ml Doxycycline). For MCM2 and TONSL, U-2-OS cells were washed twice in PBS and ex-

CellPress
OPEN ACCESS

tracted by scraping in ChWB buffered with Tris.HCl (50 mM, pH 7.5). Resultant extracts were clarified by centrifugation (15 mins, 15,000 g) and 30 mins of pre-clearing with chromatin wash buffer equilibrated Superflow 6 beads (IBA). When comparing DNAJC9 interactomes in soluble and chromatin fractions the following changes were made: adherent U-2-OS cells were harvested at 4°C by scraping in PBS/EDTA (10 mM), washed twice in PBS and extracted twice with ChWB combining supernatants after centrifugation (2,800 g, 3 mins, 4°C) to form one soluble extract. Pellets after soluble extraction were digested with MNase (3.8 U/μl, 88216, Thermo Scientific) in 1 volume of digestion buffer (1.5 hours, 30°C, 1250 rpm). Digestion buffer (DB): ChWB (49 ml) with 10 mM $CaCl_2$ (1 ml, 0.5 mM). MNase digests were quenched by the addition of EDTA (0.05 volumes, 0.5 M) and EGTA (0.05 volumes, 0.5 M) and filtered (0.45 μm) and with an additional 0.5 volumes of ChWB to wash through the filter.

Immunoprecipitation

Protein concentrations measured using Pierce 660nm Protein Assay Reagent (Thermo Scientific) or the Bradford protein assay (Bio-Rad) were equalized with their equivalent final extraction buffer. Flag-HA-MCM2, eGFP-TONSL, histones-Flag-HA and DNAJC9-Myc-Flag extracts were incubated with the following beads: anti-HA (88836, Thermo Scientific), GFP-trap (GTMA-20, Chromotek), anti-HA (26181, Thermo Scientific) and anti-Flag M2 (A2220, Sigma), respectively. IPs were washed in ice-cold chromatin wash buffer, except where stated otherwise, and additional ice-cold wash buffers dependent on their downstream analysis. DNAJC9 SILAC IPs were additionally washed in ChWB without Nonidet P40, Glycerol or EDTA, prior to LSB elution. H3.1 versus H3.1T and H3.1 versus H3.1 ED105AA IPs for mass spectrometry analysis were additionally washed 3 times with minimal wash buffer (MWB: 300 mM NaCl, 50 mM Tris pH 7.6) prior to guanidinium chloride-based elution and in-solution tryptic digestion. Label-free DNAJC9 IPs and histone IPs from siRNA treated cell extracts were additionally washed with MWB and NH_4HCO_3 (50 mM) prior to on-bead tryptic digestion. SILAC MCM2 and TONSL IPs were washed exclusively in ChWB with reduced NaCl (150 mM), Nonidet P40 (0.2%) and Tris.HCl (50 mM, pH 7.5) prior to LSB elution. SILAC samples were subjected to in-gel tryptic digestion. Pulldowns probed by western blot were eluted in LSB.

Quantitative ChIP-seq and cell fractionation

U-2-OS Flp-In T-REx DNAJC9-Myc-Flag WT, J and 4AJ mutants were compared to the parental U-2-OS Flp-In T-REx cell line in ChIP-seq experiments and cell fractionation control experiments. Cells were cultured, induced (2 μg/ml Tetracycline, 48 hr), and processed in parallel. Soluble and chromatin fractionation experiments were processed without cross-linking as follows. Cells washed in PBS (37°C) were released with Trypsin-EDTA (0.25%, 37°C, 25200056, Thermo Scientific), quenched with DMEM + 10% FBS, and cell pellets after centrifugation (300 g, 5 mins) washed in warm PBS, and ice-cold PBS and stored at −80°C. Cell pellets were extracted with 1 volume of ice-cold ChWB, and soluble supernatants transferred to fresh tubes after centrifugation (2800 g, 3 mins, 4°C) and filtration (0.45 μm). Chromatin pellets were washed with 1 volume of ChWB, spun down (2800 g, 3 mins, 4°C) and digested with Benzonase (0.015 volumes, 25 U/μl, Millipore, 70746, 1 hour, 37°C) in 1 volume ChWB supplemented with $MgCl_2$ (0.01 volume, 1 M). Resultant chromatin extracts were spun down (16,000 g, 3 mins, 4°C) and supernatants transferred to fresh tubes. Protein concentrations measured using Pierce 660nm Protein Assay Reagent (Thermo Scientific) were equalized prior to western blot analysis.

For quantitative ChIP-seq, U-2-OS cells were processed along with *Drosophila* S2 cells following the truChIP Chromatin Shearing Kit protocol (Covaris, 520127). Cells fixed (1% formaldehyde, 10 mins, RT) and quenched (Quenching Buffer E, 5 mins, RT) were washed twice in PBS and harvested by scraping. Cell pellets were snap-frozen in liquid nitrogen and stored at −80°C until lysis. Cell lysis and nuclei isolation was performed as per manufacturer's instructions, then 10-20 million nuclei were resuspended in Covaris Shearing Buffer D3 (1 ml) and sonicated (Covaris M220) in AFA milliTUBEs (Covaris, 520130). Sonication parameters: duty cycle intensity = 10%, cycles/burst = 200, processing time = 25 mins, bath temp = 7°C, and water level = full. Chromatin extracts for ChIP were clarified by centrifugation (10 min, 10,000 g at 4°C). ChIP inputs (25 μg total DNA) including spike-in control *Drosophila* S2 chromatin (1.5%) were adjusted to 500 μl with dilution buffer (4% glycerol, 10 mM Tris.HCl pH 8.0, 1 mM EDTA, 0.5 mM EGTA), and further diluted with 400 μl of incubation buffer (2.5% Triton X-100, 0.25% sodium deoxycholate, 0.25% SDS, 0.35 M NaCl, 10 mM Tris.HCl pH 8 with 1 μg/ml Leupeptin, 1 μg/ml Aprotinin, 1 μg/ml Pepstatin A, and 1 mM PMSF). Samples from ChIP inputs (1%, 10 μl) were taken for controls and stored at −20°C, the remainder was applied to 40 μl Protein-G Dynabeads (Thermo Scientific), pre-blocked (PBS, 0.5% Tween and 0.5% BSA) and pre-coupled with antibodies against Flag (5 μg, Sigma, F7425) and H2Av (2ug, Active Motif, 39715), and incubated with rotation over-night (4°C). ChIP washes, elution and decrosslinking were performed as previously reported (Kaaij et al., 2019). DNA was purified using AMPure XP beads (Beckman Coulter) and quantified with Qubit dsDNA high-sensitivity assay (Thermo Scientific). Finally, immunoprecipitated DNA was subjected to end repair, A-tailing and amplification using the KAPA Hyperprep kit protocol (Roche). Before amplification, DNA was size-selected with Agencourt AMPure XP beads (Beckman Coulter) to obtain fragments between 200-700 bp. For amplification, 8 PCR cycles were used followed by clean-up with Agencourt AMPure XP beads.

DNA sequencing, data processing and analysis

ChIP-seq libraries were sequenced 75 bp single-end on an Illumina NextSeq 500. Trimming, mapping and peak calling were performed in Galaxy (Afgan et al., 2016) as follows: adaptor sequences were trimmed using Trim Galore (Babraham Institute), reads were mapped to the hg38 assembly human genome using Bowtie2 (Langmead and Salzberg, 2012) and peak calling was performed with MACS ''broad domain'' parameters (Zhang et al., 2008) using INPUT as control. Subsequent analysis was performed using Seq-

monk (version 1.42.1). Reads with MAPQ < 20, PCR duplicates, and reads that overlapped with the Broad Institute sequencing blacklist (ENCODE Project Consortium, 2012) were discarded. For downstream analysis, the remaining reads were extended by 250 bp. To calculate reference-adjusted reads per million (RRPM) normalization factors for ChIP-seq libraries with spike-in, reads were mapped to the dm3 assembly *Drosophila* genome using Bowtie2 (Langmead and Salzberg, 2012) in Galaxy (Afgan et al., 2016). The number of uniquely mapped reads was used to calculate RRPM as in Reverón-Gómez et al. (2018). INPUT corrected DNAJC9 signal for Figure 5E was calculated as the Log_2 fold change of DNAJC9 ChIP signal over INPUT signal, in reads per million (RPM). Bedgraphs for screenshots generated in Seqmonk (version 1.42.1) were visualized with custom R scripts. Boxplots were generated in R using custom scripts.

Immunofluorescence microscopy

U-2-OS Flp-In T-REx DNAJC9-Myc-Flag WT, J, 4A and 4AJ cells were seeded at a density of 9,400 cells per well in 96 well plates (Zell-Kontakt, 5241-20), relevant wells induced with Tetracycline (1 μg/ml, 48 hours) and DNA synthesis labeled with EdU (20 mins, 40 μM). Cells washed in ice-cold PBS were either fixed directly (4% paraformaldehyde, 15 mins, 4°C) or after pre-extraction, with cytoskeleton buffer CSK/0.5% Triton X-100 (5 mins on ice), and washes in ice-cold CSK and PBS. EdU staining followed the Click-iT plus Alexa647-picolyl azide protocol (Thermo Scientific), proteins and DNA detected by immunofluorescence and DAPI (4′,6-diamidino-2-phenylindole) staining respectively. Images were acquired on an Olympus ScanR high-content microscope and analyzed with ScanR analysis software. Cell cycle gates were defined using mean EdU and total DAPI intensities. Over 2000 cells were analyzed per condition per biological replicate.

Antibodies

Western blots were performed with the following antibodies: TONSL (1:1000, HPA046494, Sigma), CAF1A/p150 (1:1000, (Quivy et al., 2004)), MCM2 (1:1000, A300-122A, Bethyl Laboratories), CAF1B/p60 (1:1000, (Quivy et al., 2004)), DNAJC9 (1:1000, ab150394, Abcam), HA (1:3000-5000, C29F4 #3724, Cell Signaling Technology), DAXX (1:250, HPA008736, Sigma), HJURP (1:1000-2000, HPA008436, Sigma), Flag (1:3000-20000, F7425, Sigma), H3 (1:1000-, ab1791, Abcam), H4 (05-858, Millipore), Tubulin (1:10000, ab6160, Abcam), BAG2 (1:1000, ab79406, Abcam), HSC/HSP70 (1:200, sc-24, Santa Cruz Biotechnology) and HSC70 (1:500-1000, sc-7298, Santa Cruz Biotechnology). Immunofluorescence was performed with the following antibodies: Flag (1:500, F3165, Sigma), Goat Anti-Mouse-Alexa488 (1:1000, A-11029, Thermo Scientific).

MS sample preparation

Samples were digested using sequencing-grade modified trypsin, either in-gel, in-solution, or on-beads, according to standard procedures. Peptides were desalted and purified at low-pH or high-pH on StageTips (Rappsilber et al., 2007), assembled using four layers of C18 (punch-outs from 47mm C18 3M extraction discs, Empore). All buffers and samples were passed over StageTips by centrifugation at 1,500 g. StageTips were activated using 100 μl methanol, followed by 100 μl of 80% ACN in 0.1% formic acid (low pH) or 200 mM ammonium (high pH). StageTips were equilibrated using 2 × 100 μl of 0.1% formic acid (low pH) or 50 mM ammonium (high pH), after which samples were loaded, washed twice with 150 μl 0.1% formic acid or 50 mM ammonium, and eluted with 40 μl of 40% ACN in 0.1% formic acid (low pH) or 80 μl of 25% ACN in 50 mM ammonium (high pH). All samples were vacuum-dried to completion in LoBind tubes, using a SpeedVac at 60°C for 2 h, dissolved by the addition of 10 μl 0.1% formic acid, and stored at −20°C until mass spectrometric measurement. Details per experiment are listed in the experimental design table available on ProteomeXchange via identifier PXD020268.

MS analysis

The majority of samples were analyzed on EASY-nLC 1200 system (Thermo), coupled to a Q Exactive HF-X Hybrid Quadrupole-Orbitrap mass spectrometer (Thermo). Exceptions to instrumentation and settings used are listed in the experimental design table available on ProteomeXchange via identifier PXD020268. Separation of peptides was performed using 15-cm columns (75 μm internal diameter) packed in-house with ReproSil-Pur 120 C18-AQ 1.9 μm beads (Dr. Maisch). Elution of peptides from the column was achieved using a gradient ranging from buffer A (0.1% formic acid) to buffer B (80% acetonitrile in 0.1% formic acid), at a flow of 250 nl/min. Gradient length was 100 min per sample, including ramp-up and wash-out, with an analytical gradient of 79 min with a buffer B ramp from 7% to 38% buffer B. The column was heated to 40°C using a column oven, and ionization was achieved using a Nanospray Flex Ion Source (Thermo). Spray voltage was set to 2 kV, ion transfer tube temperature to 275°C, funnel RF level to 40%, full scan range to 300-1,750 *m/z*, MS1 resolution to 60,000, MS1 AGC target to 3,000,000, and MS1 maximum injection time to 60 ms. Precursors with charges 2-6 were selected for fragmentation using an isolation width of 1.3 *m/z*, and fragmented using higher-energy collision disassociation (HCD) using a normalized collision energy of 25. Precursors were excluded from re-sequencing by setting a dynamic exclusion of 100 s. MS2 resolution was set to 30,000, MS2 AGC target to 200,000, minimum MS2 AGC target to 20,000, MS2 maximum injection time to 58 ms, and loop count to 14.

QUANTIFICATION AND STATISTICAL ANALYSIS

Analysis of MS data

All MS RAW data was analyzed using the freely available MaxQuant software (Cox and Mann, 2008; Cox et al., 2011), version 1.6.3.4. Default MaxQuant settings were used, with exceptions specified below. For the generation of the theoretical spectral library, the human FASTA database was downloaded from UniProt on the 13th of May, 2019. Label-free quantification was enabled. Stringent MaxQuant 1% FDR filtering was applied at all levels (default). Matching between runs and second peptide search were enabled. For SILAC samples, multiplicity was set to 2 (exp. 1 and 2) or 3 (exp. 3), with SILAC labels set to Arg0;Lys0 (light) and Arg6;Lys8 (heavy) or Arg0;Lys0 (light) and Arg6;Lys4 and Arg10;Lys8 (heavy), respectively.

Statistical analysis of MS data

MaxQuant text output (proteinGroups.txt) was further analyzed using the freely available Perseus software (Tyanova et al., 2016), version 1.6.2.3. Details regarding statistical handling of data are outlined in each figure legend. In general, the proteomics data was filtered to exclude contaminant hits, reverse-database hits, and proteins only identified by site, Log_2 transformed, and filtered for detection in all replicates for at least one condition. Where stated, missing values were imputed with a down shift of 1.8 and a width of 0.3. Student's two-sample t testing was performed with permutation-based FDR control, with s0 values stated for each experiment, to derive *p-value*s corrected for multiple-hypothesis testing (i.e., *q*-values).

Data visualization

Volcano and scatterplots were visualized in GraphPad Prism (v8.4). Bubble plots and heatmaps were visualized with R version 3.6.2 using the libraries ggplot2 version v3.2.1, scales version 1.1.0, RColorBrewer version 1.1.2, and pheatmap version 1.0.12. Network analysis was performed in Cytoscape (Shannon et al., 2003) version 3.8.0 with the stringApp (Doncheva et al., 2019) version 1.5.1 and the Omics Visualizer app (Legeay et al., 2020) version 1.3.0. All other statistical analysis was performed in GraphPad Prism and test details are referred to in figure legends.

Molecular Cell, Volume *81*

Supplemental information

DNAJC9 integrates heat shock molecular chaperones into the histone chaperone network

Colin M. Hammond, Hongyu Bao, Ivo A. Hendriks, Massimo Carraro, Alberto García-Nieto, Yanhong Liu, Nazaret Reverón-Gómez, Christos Spanos, Liu Chen, Juri Rappsilber, Michael L. Nielsen, Dinshaw J. Patel, Hongda Huang, and Anja Groth

Figure S1

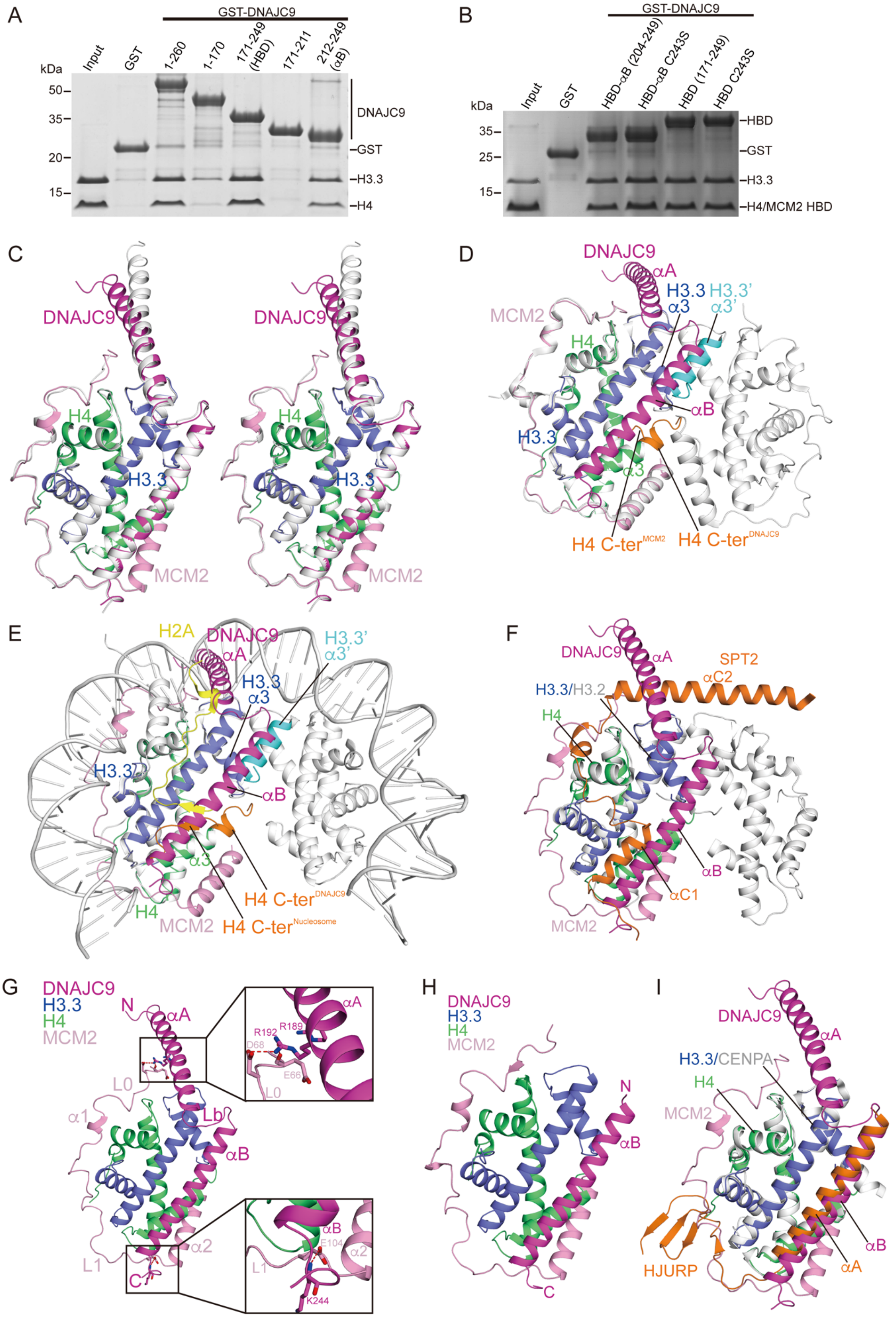

A
GST-DNAJC9
Input
GST
1-260
1-170
171-249 (HBD)
171-211
212-249 (αB)
kDa
50
35
20
15
DNAJC9
GST
H3.3
H4
B
GST-DNAJC9
Input
GST
HBD-αB (204-249)
HBD-αB C243S
HBD (171-249)
HBD C243S
kDa
35
25
15
HBD
GST
H3.3
H4/MCM2 HBD
C
DNAJC9
H4
H3.3
MCM2
D
DNAJC9
αA
H3.3 H3.3'
α3 α3'
MCM2
H4
αB
H3.3
α3
H4 C-ter^MCM2
H4 C-ter^DNAJC9
E
H2A
DNAJC9
αA
H3.3'
α3'
H3.3
α3
H3.3
αB
α3
H4
MCM2
H4 C-ter^DNAJC9
H4 C-ter^Nucleosome
F
DNAJC9
αA
SPT2
αC2
H3.3/H3.2
H4
αB
αC1
MCM2
G
DNAJC9
H3.3
H4
MCM2
N
αA
L0
α1
Lb
αB
α2
L1
C
R192 R189
D68
E66
E104
K244
H
DNAJC9
H3.3
H4
MCM2
N
αB
C
I
DNAJC9
αA
H3.3/CENPA
H4
MCM2
αB
HJURP
αA

Figure S1. Biochemical and Structural Characterisation of the DNAJC9 HBD – Related to Figure 2 and Table 1

(A) Truncation mapping of DNAJC9 histone binding domain (HBD) using GST-pulldown. The pulldowns were between GST-DNAJC9 fragments and histone H3.3–H4, similar to Figure 2B but without the MCM2 HBD.

(B) Pulldowns showing that C243S mutation on DNAJC9 HBD or αB did not influence histone binding.

(C) Stereo view showing structural comparison of the two copies of the DNAJC9 HBD–H3.3–H4–MCM2 HBD quaternary complexes in the crystallographic asymmetric unit. One complex is color-coded DNAJC9 HBD (magenta), H3.3 (blue), H4 (green) and MCM2 HBD (pink), while the other is coloured silver. The color-coded complex is the form presented in Figure 2C. The two complex structures superimposed well with a small root-mean-square deviation (rmsd) of 0.47 Å.

(D) Superimposition of the DNAJC9 HBD–H3.3–H4–MCM2 HBD complex (colored in C) with the MCM2 HBD–H3.3–H4 complex (silver; PDB 5BNV (Huang et al., 2015)). The αB helix of DNAJC9 HBD sterically occludes the H3.3' α3' helix (cyan) to block H3.3–H4 tetramerisation seen in the MCM2 HBD–H3.3–H4 complex. The H4 C-terminus ('C-ter'; orange) adopts a helical conformation upon DNAJC9 HBD binding, while it is partially disordered in the MCM2 HBD–H3.3–H4 complex.

(**E**) Superimposition of the DNAJC9 HBD–H3.3–H4–MCM2 HBD complex (colored in C) with the H3.3–H4 tetramer from a partial representation of the nucleosome (silver; PDB 3AV2 (Tachiwana et al., 2011)). The αB helix of DNAJC9 HBD sterically occludes the H3.3' α3' helix (cyan) to block H3–H4 tetramerisation observed in the nucleosome. The H4 C-terminus ('C-ter'; orange) adopts a helical conformation upon DNAJC9 HBD binding, while it forms a β-strand with the H2A docking domain (yellow) in the nucleosome.

(**F**) Superimposition of the DNAJC9 HBD–H3.3–H4–MCM2 HBD complex (colored in C) and the SPT2–H3.2–H4 complex (H3.2–H4 tetramer, silver; SPT2, orange; PDB 5BS7 (Chen et al., 2015)), showing that observed binding modes of DNAJC9 HBD and SPT2 HBD with histone H3–H4 are mutually incompatible.

(G) DNAJC9 HBD contacts MCM2 HBD in two points with interacting residues highlighted (see magnified inserts; colored in C).

(H) Overall structure of the DNAJC9 αB–H3.3–H4–MCM2 HBD quaternary complex in cartoon representation (DNAJC9 αB, magenta; H3.3, blue; H4, green; MCM2 HBD, pink). This structure is almost identical to its counterpart containing the DNAJC9 HBD (rmsd 0.38 Å, Figure 2C). Data collection and refinement statistics presented in Table 1.

(I) Superimposition of the DNAJC9 HBD–H3.3–H4–MCM2 HBD complex (colored in C) and the HJURP–CENPA–H4 complex (CENPA–H4 dimer, silver; HJURP, orange; PDB 3R45 (Hu et al., 2011)), revealing that the αB helix of DNAJC9 HBD has a similar structural configuration to the αA helix of HJURP.

Figure S2

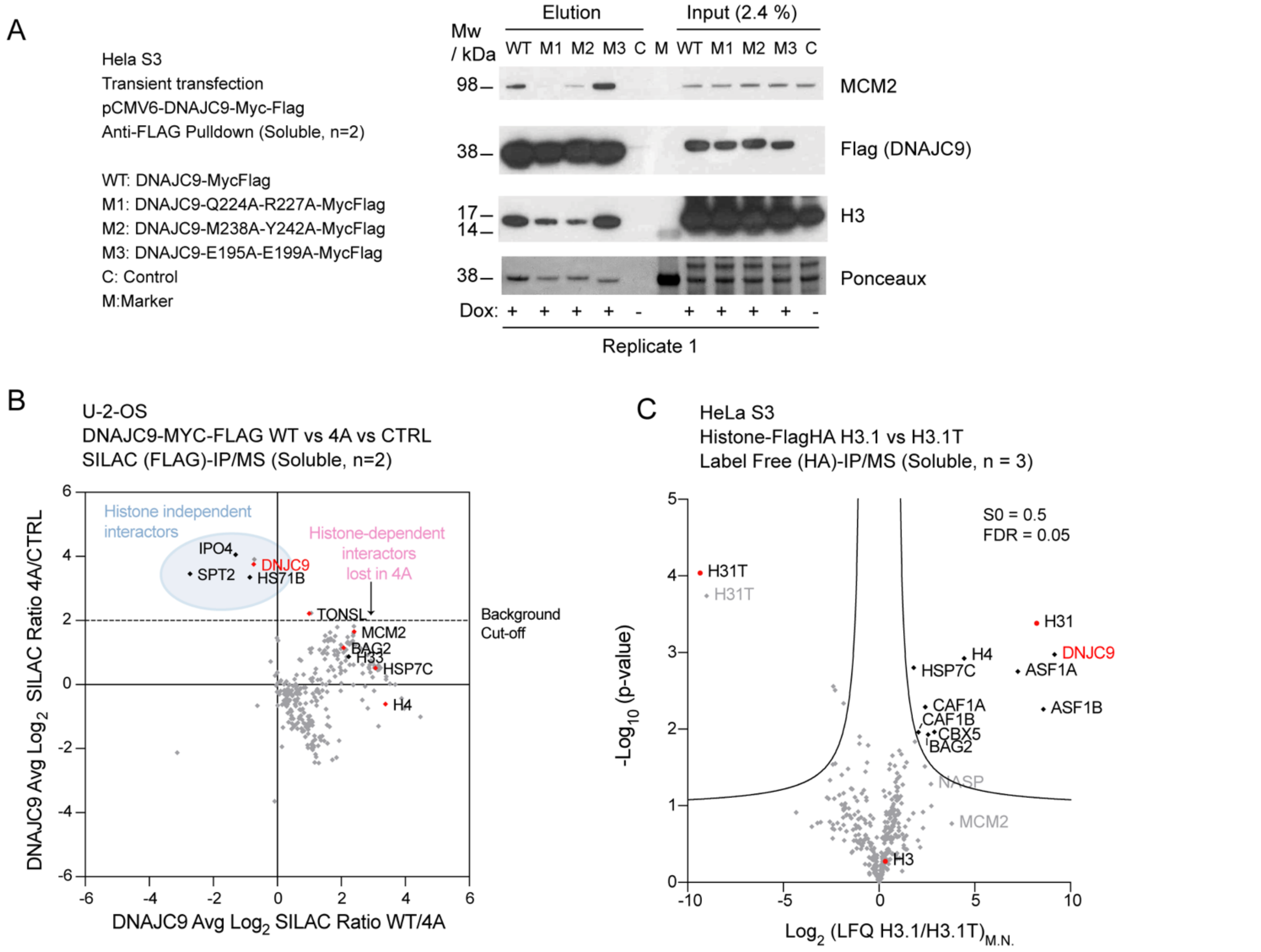

Figure S2. Analysis of DNAJC9 Histone Binding Mutants and H3.1T Interactome - Related to Figure 4

(A) Pull-downs from soluble extracts of DNAJC9 WT and mutant proteins expressed by transient transfection and analysed by Western blotting (representative of n=2 biological replicates).

(B) DNAJC9 WT, 4A mutant and control purifications subjected to triple SILAC based mass spectrometry analysis. Data from the same experiment is shown with WT/C on the x-axis in Figure 4A. This plot highlights a histone-independent interaction between DNAJC9 and SPT2. Ratios averaged from n=2 biological replicates.

(C) Analysis of the H3.1T interactome. Histone purifications from soluble extracts subjected to label-free mass spectrometry analysis (n=3 biological replicates, s0 = 0.5, FDR = 0.05). Volcano plots represent differences in median normalised LFQ intensities ($LFQ_{M.N.}$) with missing values imputed for factors observed 3 times in either condition. Diamonds, protein level LFQ quantification; Red dots, median ratios and p values from peptide level LFQ intensities for peptides specific to either H3.1 (H31) or H3.1T (H31T), or shared H3 peptides (H3).

(B and C) Proteins referred to by human UniProt protein identification code. See also Table S1.

Figure S3

A

HeLa S3 H3.1 or H4-FlagHA
+siRNA or siCTRL - Label Free (HA)-IP/MS
(Soluble, H4 n = 4, H3.1 n = 5)

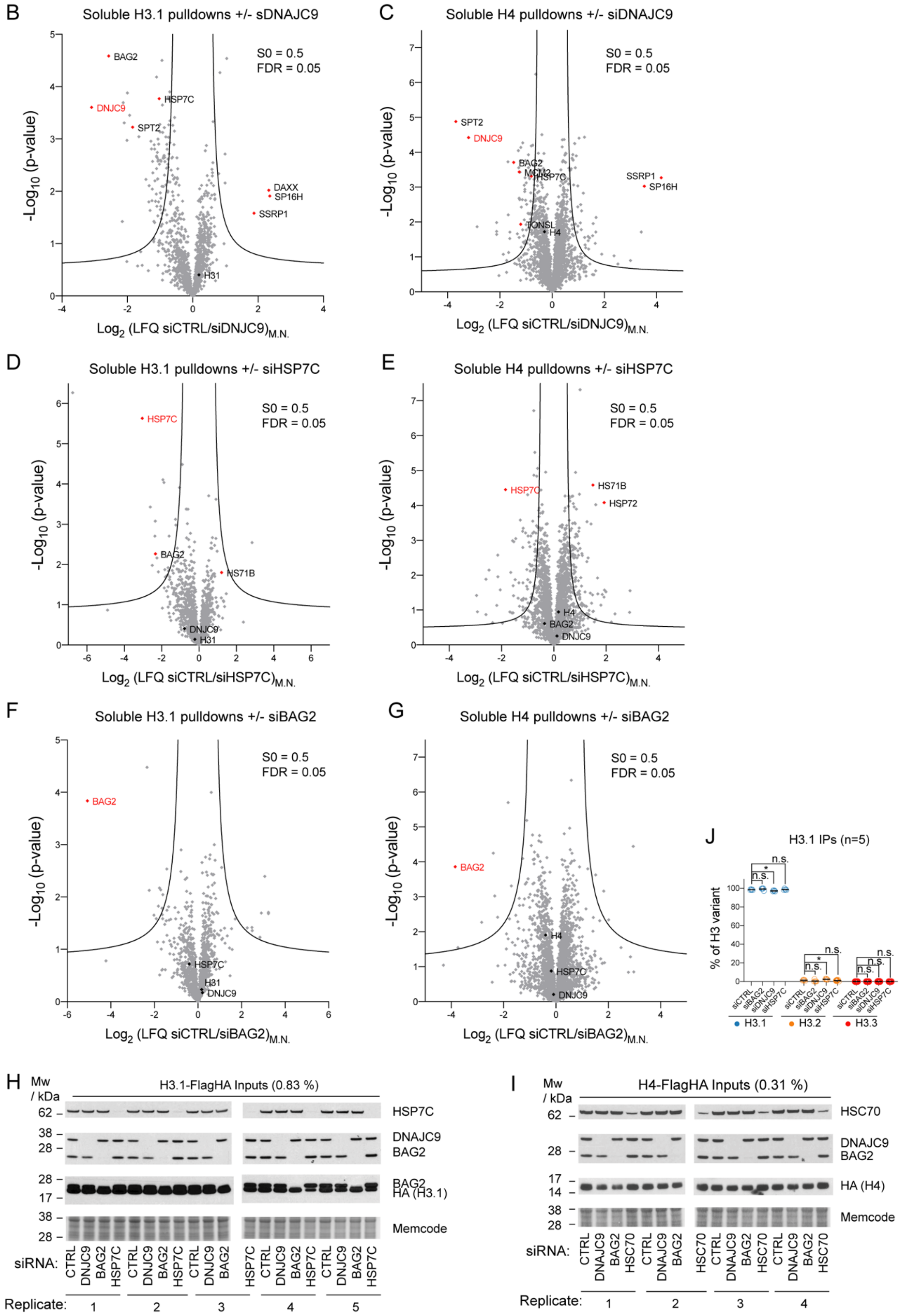

Figure S3. Functional Proteomic Analysis of Histone H3.1 and H4 Complexes Using siRNA Depletion of DNAJC9 and the Heat Shock Machinery – Related to Figure 4

(A-G) (A) Histone H3.1 and H4 purifications from soluble extracts siRNA depleted of DNAJC9 (**B-C**), BAG2 (**D-E**) or HSC7C (**F**-**G)** compared to control siRNA depletions and subjected to label-free mass spectrometry analysis (H3.1 n=5 and H4 n=4 biological replicates, s0 = 0.5, FDR = 0.05). Volcano plots represent differences in median-normalised LFQ intensities ($LFQ_{M.N.}$) with missing values imputed for factors observed in all replicates of either condition for each volcano plot comparison.

(**H and I**)**,** Western blot controls for siRNA depletion in extracts used for (**H**) H3.1 and (**I)** H4 pulldowns.

(**J**) Quantitation of the variant specific peptides showing a small but significant increase in H3.2 (P = 0.039) but not H3.3 peptides (P = 0.969) in H3.1 pulldowns depleted for DNAJC9 with no significant variant peptide gains in H3.1 pulldowns depleted for HSP7C (H3.2 P = 0.969, H3.3 P >0.999) and BAG2 (H3.2 P = 0.215, H3.3 P >0.999). P values represent unpaired t-tests Holm-Šídák corrected, alpha = 0.05, assuming sampling from populations with constant standard deviation. See also Table S2.

(**B-G**) Proteins referred to by human UniProt protein identification code. See also Table S1.

Figure S4

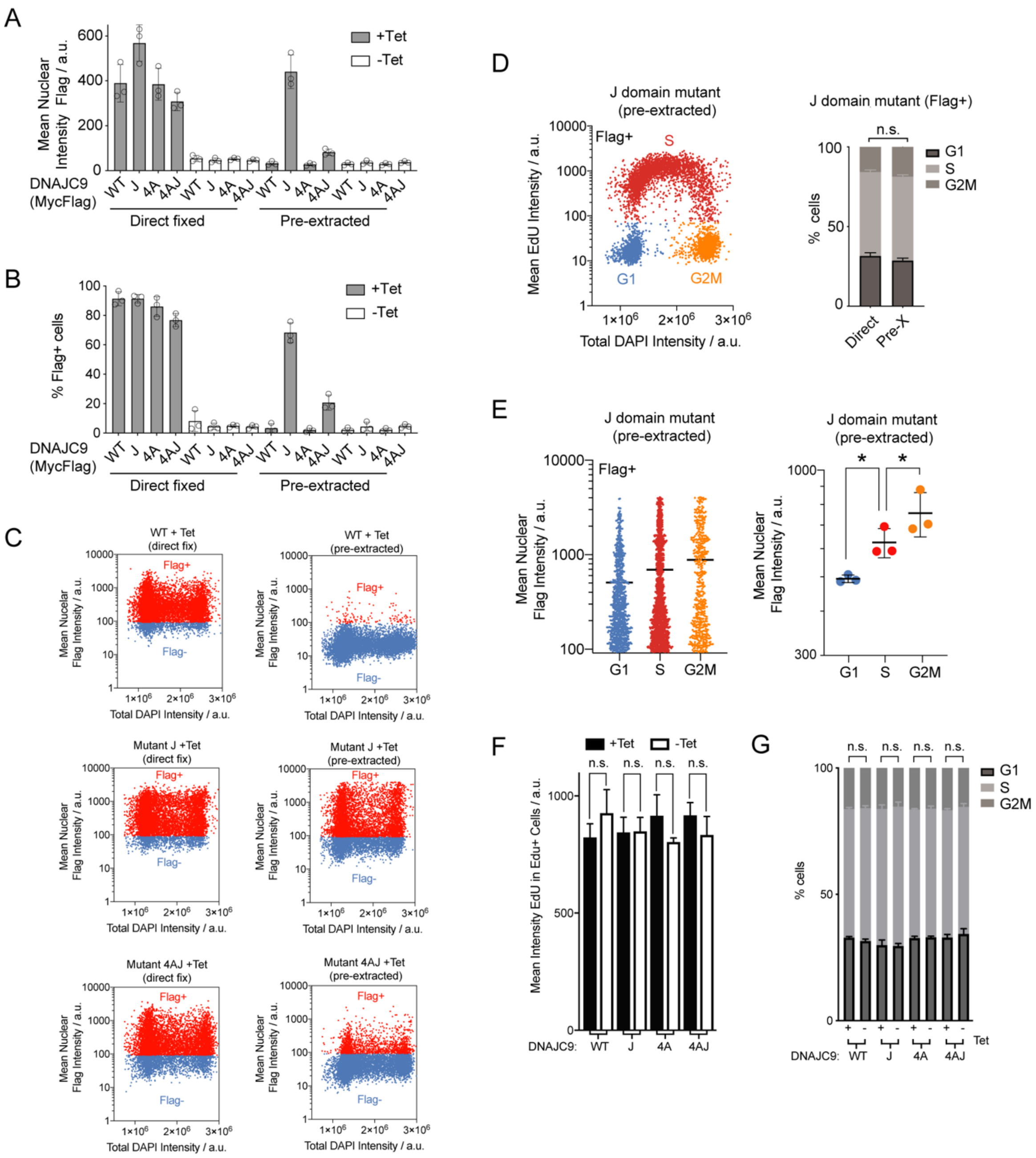

A
Mean Nuclear Intensity Flag / a.u.
+Tet
-Tet
DNAJC9 (MycFlag)
WT J 4A 4AJ WT J 4A 4AJ WT J 4A 4AJ WT J 4A 4AJ
Direct fixed
Pre-extracted
B
% Flag+ cells
+Tet
-Tet
DNAJC9 (MycFlag)
Direct fixed
Pre-extracted
C
WT + Tet (direct fix)
WT + Tet (pre-extracted)
Mutant J +Tet (direct fix)
Mutant J +Tet (pre-extracted)
Mutant 4AJ +Tet (direct fix)
Mutant 4AJ +Tet (pre-extracted)
Flag+
Flag-
Mean Nuclear Flag Intensity / a.u.
Total DAPI Intensity / a.u.
D
J domain mutant (pre-extracted)
Flag+
S
G1
G2M
Mean EdU Intensity / a.u.
Total DAPI Intensity / a.u.
J domain mutant (Flag+)
n.s.
G1
S
G2M
% cells
Direct
Pre-X
E
J domain mutant (pre-extracted)
Flag+
Mean Nuclear Flag Intensity / a.u.
G1 S G2M
J domain mutant (pre-extracted)
*
*
Mean Nuclear Flag Intensity / a.u.
G1 S G2M
F
+Tet
-Tet
n.s.
Mean Intensity EdU in Edu+ Cells / a.u.
DNAJC9: WT J 4A 4AJ
G
n.s.
G1
S
G2M
% cells
Tet
DNAJC9: WT J 4A 4AJ

Figure S4. The DNAJC9 J Mutant is Trapped on Chromatin in a Histone Dependent Manner Throughout the Cell Cycle – Related to Figure 5

(A-G) High-content microscopy of DNAJC9-MYC-Flag WT and mutants induced by tetracycline (+Tet) in direct fixed (total) or pre-extracted (chromatin-bound) U-2-OS cells pulsed with EdU (n=3 biological replicates). See also Table S2.

(A) Mean flag intensity in nuclei defined by DAPI (error bars represent mean ± s.d).

(B) Percentage of Flag positive cells (error bars represent mean ± s.d).

(C) Gating strategy for identification of Flag positive cells in **(B)**

(D) Left, Representative cell cycle gating showing G1, S and G2/M populations of pre-extracted Flag positive DNAJC9 J mutant cells. Right, Mean cell cycle populations ± s.d. of Flag positive DNAJC9 J mutant cells comparing direct or pre-extracted cells (for G1, S and G2M comparisons, adjusted P = 0.092, >0.999, 0.092, unpaired t-tests Holm-Šídák corrected, alpha = 0.05, assuming sampling from populations with constant standard deviation).

(E) Mean nuclear Flag intensities in cell cycle gated populations for pre-extracted DNAJC9 J mutant cells (Flag positive) (Left; representative example, means indicated) and experimental means shown (Right; from left, P = 0.0404 and 0.0456, paired two-sided t-tests, error bars represent mean ± s.d.).

(F) Mean EdU intensities ± s.d. in EdU positive cells comparing intensities with and without the expression (+/- Tet) of DNAJC9 WT and mutants (from left, adjusted P = 0.7127, 0.9872, 0.6763, 0.7736, unpaired t-tests Holm-Šídák corrected, alpha = 0.05, assuming sampling from populations with constant standard deviation).

(G) Mean cell cycle populations ± s.d. in direct fixed cells, comparing population percentages with and without expression (+/- Tet) of DNAJC9 WT and mutants (from left for G1, adjusted P = 0.9470, 0.9998, 0.9998 and 0.9439; from left for S, adjusted P = 0.7540, 0.9608, >0.9999 and 0.9998; from left for G2M, adjusted P = 0.9998, 0.9962, 0.9998 and 0.9594, unpaired t-tests Holm-Šídák corrected, alpha = 0.05, assuming sampling from populations with constant standard deviation)

Figure S5

A

MS Analysis Workflow

(1) Raw data (3224 proteins)

(2) Filtering reverse hits, only identified by site and contaminants (3076 proteins)

(3) Filtering for 6/6 observations in WT or J or 4A (1654 proteins)

(4) Filtering for enrichment over beads in any condition (464 proteins combined) - panel B

(5) Bait normalisation - panel C

(6) WT vs 4A and WT vs J volcano plots - no imputed values - panels D & E

(7) For factors not identified in 6/6 control samples WT/J and WT/4A ratios were imputed, filtered and normalised similarly (177 proteins)

B

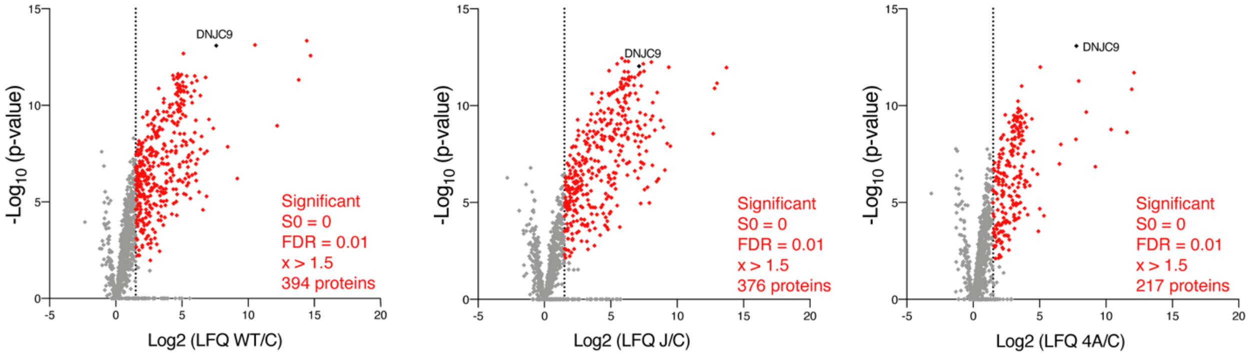

C

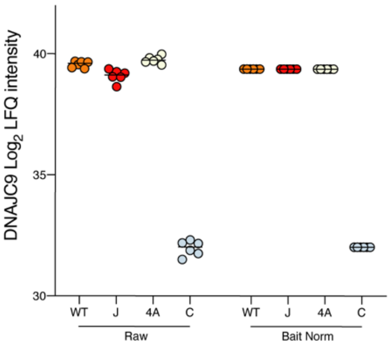

D

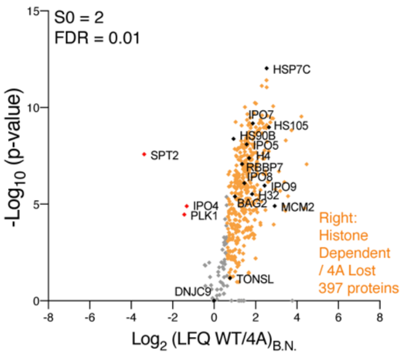

E

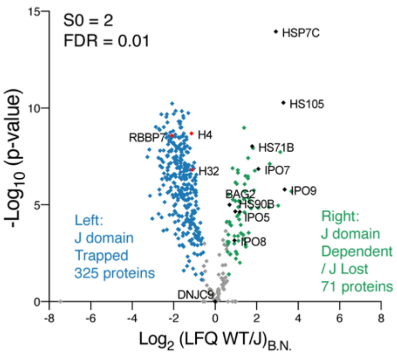

F

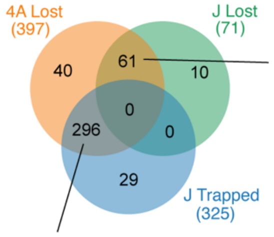

GO Biological Process
Top 5 Terms (count, fold enrichment)
1) Protein import into nucleus (6, 12.5)
2) Viral process (10, 3.9)
3) Regulation of cellular response to heat (6, 8.0)
4) Intracellular protein transport (7, 6.0)
5) Chaperone mediated protein folding requiring cofactor (3, 36.1)

GO Biological Process
Top 5 Terms (count, fold enrichment)
1) Translation (96, 5.7)
2) Nuclear-transcribed mRNA catabolic process, nonsense mediated decay (69, 6.9)
3) SRP-dependent cotranslational protein targetting to membrane (63, 7.3)
4) Translational initiation (72, 6.1)
5) rRNA processing (87, 4.9)

Figure S5. Mass Spectrometry Analysis of DNAJC9 WT, J and 4A vs Control Purifications from Soluble Extracts – Related to Figure 6

(A) Overview of data analysis workflow. Data from n=6 biological replicates.

(B) Volcano plots of showing enrichment of factors associated with soluble DNAJC9 WT and mutants over beads. Factors highlighted in red are significant (s0 = 0, FDR = 0.01) and have a Log2 fold change of >1.5 in their ratio of LFQ intensity (DNAJC9/control). LFQ intensities of factors that met these filtering criteria for WT, J or 4A purifications were subsequently bait normalised.

(C) DNAJC9 LFQ intensity in purifications before and after bait normalization. See also Table S2.

(D and E) Volcano plots of showing enrichment of factors with DNAJC9 WT compared to mutants 4A **(D)** and J **(E)**, ratios represent bait normalized LFQ intensities. Colours were used to highlight factors that show statistical differences in DNAJC9 WT and mutant comparisons (s0 = 2, FDR = 0.01), with the exception of DNAJC9 proteins labelled show significant differences.

(F) Venn diagrams (InteractiVenn (Heberle et al., 2015)) colour coded as in **(D-E)**, showing the overlap of factors classified as statistically changing between WT vs mutant. The top 5 GO biological process direct terms from indicated regions of Venn diagram from DAVID (Huang da et al., 2009a, b) version 6.8 analysis with the number of GO term associated proteins and fold change indicated compared to all factors identified (after filtering reverse hits, only identified by site and contaminants - step 2 in **(A)**); EASE = 0.01. See also Table S2.

(B, D and E) Proteins referred to by human UniProt protein identification code. See also Table S1.

Figure S6

A

MS Analysis Workflow

(1) Raw data (2693 proteins)

(2) Filtering reverse hits, only identified by site and contaminants (2562 proteins)

(3) Filtering for 6/6 observations in WT or J or 4A (1613 proteins)

(4) Filtering for enrichment over beads in any condition - panel B (422 proteins combined)

(5) Bait normalisation - panel C

(6) WT vs 4A and WT vs J volcano plots - no imputed values - panels D & E

(7) For factors not identified in 6/6 control samples WT/J and WT/4A ratios were imputed, filtered and normalised similarly (26 extra proteins) - panel G

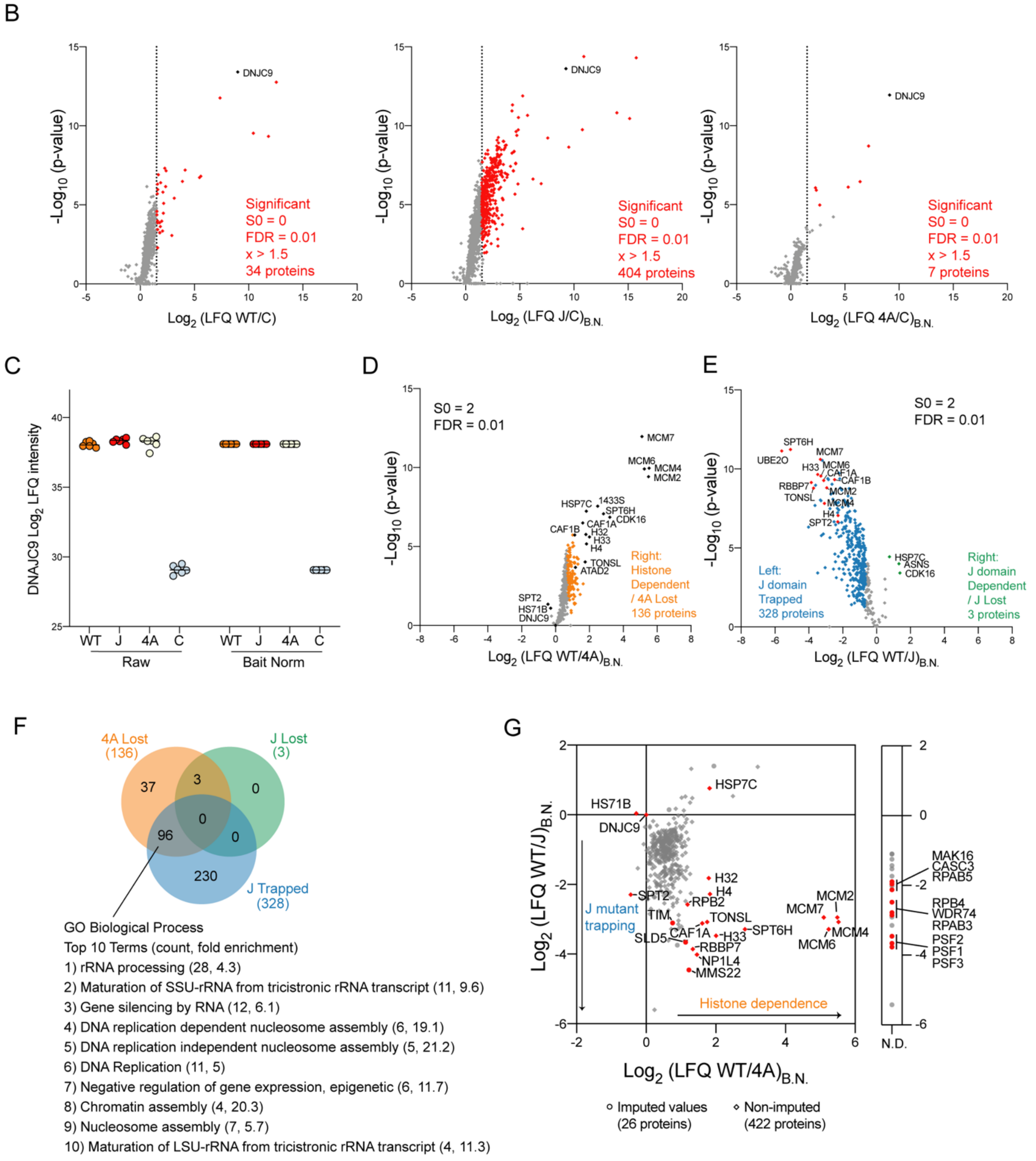

Figure S6. Mass Spectrometry Analysis of DNAJC9 WT, J and 4A vs Control Purifications from Chromatin Extracts – Related to Figure 6

(A) Overview of data analysis workflow. Data from n=6 biological replicates.

(B) Volcano plots of showing enrichment of factors associated with chromatin bound DNAJC9 WT and mutants over beads. Factors highlighted in red are significant (s0 = 0, FDR = 0.01) and have a Log2 fold change of >1.5 in their ratio of LFQ intensity (DNAJC9/control). LFQ intensities of factors that met these filtering criteria for WT, J or 4A purifications were subsequently bait normalised.

(C) DNAJC9 LFQ intensity in purifications before and after bait normalization. See also Table S2.

(D and E) Volcano plots of showing enrichment of factors with DNAJC9 WT compared to mutants 4A **(D)** and J **(E)**, ratios represent bait normalized LFQ intensities. Colours were used to highlight factors that show statistical differences in DNAJC9 WT and mutant comparisons (s0 = 2, FDR = 0.01), with the exception of DNAJC9 proteins labelled show significant differences.

(F) Venn diagrams (InteractiVenn (Heberle et al., 2015)) colour coded as in **(D-E)**, showing the overlap of factors classified as statistically changing between WT vs mutant. The top 5 GO biological process direct terms from indicated regions of Venn diagram from DAVID (Huang da et al., 2009a, b) version 6.8 analysis with the number of GO term associated proteins and fold change indicated compared to all factors identified (after filtering reverse hits, only identified by site and contaminants - step 2 in **(A)**); EASE = 0.01. See also Table S2.

(G) Scatter plot of DNAJC9 WT vs 4A and WT vs J ratios; all factors that met the statistical filtering without imputation (diamonds) and additionally factors imputed (circles) that were not identified in 6/6 control samples but otherwise met filtering requirements set out in (**A**).

(B, D, E and G) Proteins referred to by human UniProt protein identification code. See also Table S1.
